# The N-terminal Helix-Turn-Helix Motif of Transcription Factors MarA and Rob Drives DNA Recognition

**DOI:** 10.1021/acs.jpcb.1c00771

**Published:** 2021-06-17

**Authors:** Marina Corbella, Qinghua Liao, Cátia Moreira, Antonietta Parracino, Peter M. Kasson, Shina Caroline Lynn Kamerlin

**Affiliations:** † Science for Life Laboratory, Department of Chemistry—BMC, Uppsala University, Uppsala, S-751 23, Sweden; ‡ Science for Life Laboratory, Department of Cell and Molecular Biology, Uppsala University, Uppsala, S-65124, Sweden; § Departments of Molecular Physiology and Biomedical Engineering, University of Virginia, Charlottesville, Virginia 22908, United States

## Abstract

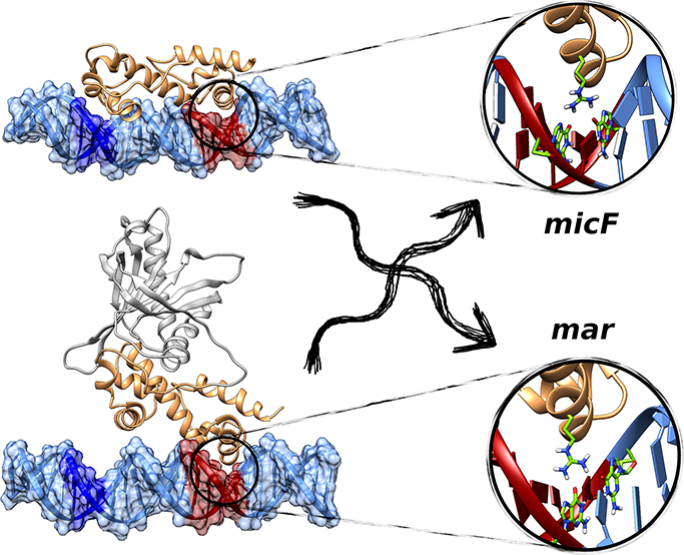

DNA-binding proteins play an important role in gene regulation and cellular function. The transcription factors MarA and Rob are two homologous members of the AraC/XylS family that regulate multidrug resistance. They share a common DNA-binding domain, and Rob possesses an additional C-terminal domain that permits binding of low-molecular weight effectors. Both proteins possess two helix-turn-helix (HTH) motifs capable of binding DNA; however, while MarA interacts with its promoter through both HTH-motifs, prior studies indicate that Rob binding to DNA via a single HTH-motif is sufficient for tight binding. In the present work, we perform microsecond time scale all-atom simulations of the binding of both transcription factors to different DNA sequences to understand the determinants of DNA recognition and binding. Our simulations characterize sequence-dependent changes in dynamical behavior upon DNA binding, showcasing the role of Arg40 of the N-terminal HTH-motif in allowing for specific tight binding. Finally, our simulations demonstrate that an acidic C-terminal loop of Rob can control the DNA binding mode, facilitating interconversion between the distinct DNA binding modes observed in MarA and Rob. In doing so, we provide detailed molecular insight into DNA binding and recognition by these proteins, which in turn is an important step toward the efficient design of antivirulence agents that target these proteins.

## Introduction

The DNA-binding specificity of transcription factors (TFs) is key to gene regulation and thus cellular function.^1−4^ Regulation of this DNA binding is the end point of many signal-transduction pathways, linking extracellular stimuli to gene-expression responses.^5−7^ The molecular details of protein–DNA recognition and selectivity are thus of great biochemical interest and biological significance, as are the mechanisms by which DNA-binding proteins can rapidly identify their specific DNA binding sites from among a multitude of nonspecific DNA sites.^8^ There has been substantial experimental^9,10^ and computational^11−13^ progress toward understanding how transcription factors search DNA for their target sites. This has been aided by recent improvements in computational power that enable the study of protein–DNA recognition on the microsecond time scale using all-atom models.^11^

Here, we seek to understand how the transcription factors MarA and Rob discriminate between different DNA sequences, using long-time scale all-atom molecular dynamics (MD) simulations. *E. coli* MarA and Rob are two homologous members of the AraC/XylS family of proteins.^14^ MarA and Rob each regulate multiple genes (termed the MarA and Rob regulons^15,16^), and are involved in resistance to antibiotics, heavy metals, organic solvents, and oxidative stress.^17^ Their function is thus particularly important to understanding environmental and multidrug resistance.^18^

**Figure 1:**
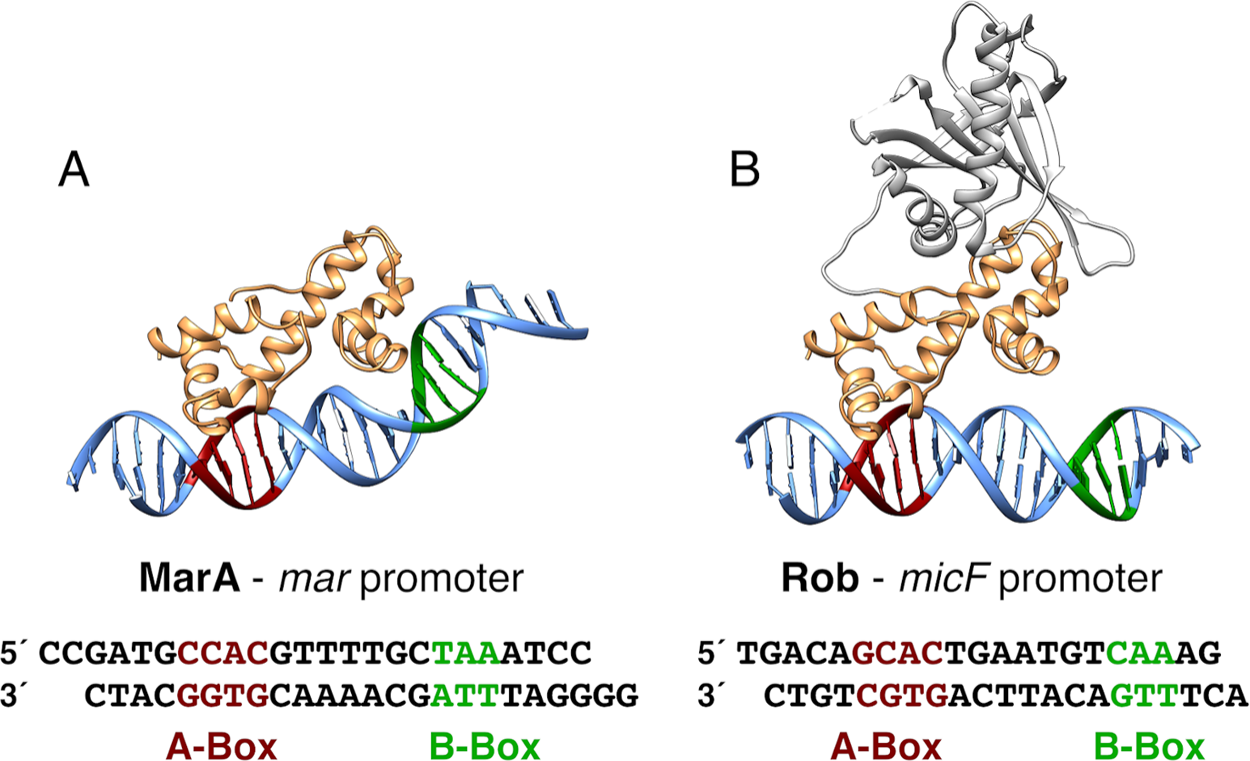
Illustrations of (A) MarA in complex with the *mar* promoter and (B) Rob in complex with the *micF* promoter (PDB IDs 1BL0(19,31) and 1D5Y,^23,31^ respectively). Shown here are also the sequences of the respective promoters for each protein, with the A- and B-box sequences (where the proteins bind) highlighted in red and green, respectively. Note that the sequences correspond to the sequences in the respective crystal structures, hence the offset between the strands; for the full aligned sequences for each promoter see Table S1. This figure was generated using Chimera.^32^

Structurally, both MarA and Rob have a ∼100 residue DNA-binding domain^19^ that is conserved among all AraC/XylS proteins.^19−22^ MarA consists only of this domain (Figure 1), but Rob also has a C-terminal domain (∼180 amino acids) believed to be involved in effector binding.^23^ The N-terminal DNA binding domain of Rob has 51% sequence identity and 71% sequence similarity with MarA.^23^ Due to the high level of sequence similarity between MarA and Rob, there is overlap between their regulons,^24−26^ although the two transcription factors have different activation efficiencies for individual genes.^27−30^

X-ray structures of MarA and Rob in complex with target DNA^19,23^ indicate that the DNA binding domains of both proteins contain two helix-turn-helix (HTH) motifs, connected by a long, rigid central helix that fixes the relative orientation of the two motifs (Figure 1). Such HTH motifs are common to DNA binding proteins in general.^33,34^ In the crystal structure, MarA bends the DNA by 35° to permit both HTH motifs to insert into the major groove simultaneously.^19^ In contrast, Rob inserts its N-terminal HTH motif into the major groove of an *unbent* DNA duplex, such that the C-terminal HTH motif lies on the surface of the duplex and interacts only with the phosphodiester groups of the DNA backbone and not with major-groove bases.^23^ It has been suggested that these might represent two alternate modes of DNA binding for AraC/XylS transcription factors.^23^ Prior work on the binding of MarA and Rob to the *micF* and *mar* promoters combined with mutagenesis studies of the DNA B-box (Table S1)^23^ explored the differences in DNA binding affinities (*K*_DNA_) between MarA and Rob. It was shown that, changes to the promoter sequence at the B-box have relatively minor impact on *K*_DNA_ (Table S2).^23^ Furthermore, while MarA binds DNA at both the A-box and the B-box of the DNA sequences shown in Table S1, Rob interacts primarily with only the A-box yet has a similar DNA-binding affinity to MarA (Figure 1 and Table S2). This suggests that DNA binding is mainly driven by interactions with the A-box,^23^ but the molecular details by which this is achieved have not yet been resolved.

We note that the lion’s share of work on understanding MarA and Rob binding specificity has been experimental, through either structural or biochemical characterization of these systems.^19,23,35−39^ Here, molecular simulations can also play an important role in enhancing our understanding of protein–DNA interactions, as reviewed in, for example, refs (37 and 40−42) among others. However, to the best of our knowledge, there exist no molecular simulation studies of the binding specificity of either MarA or Rob in the literature, and here, molecular dynamics simulations can be a useful tool to dissect the origin of the differences in DNA binding modes between the two proteins (Figure 1).

The present study builds upon recent work, in which we performed multimicrosecond all-atom simulations of LacI–DNA interactions, exploring the interactions between LacI and both specific and nonspecific DNA sequences.^11^ These simulations suggested, in agreement with experimental observations,^43^ that stable LacI binding occurs primarily to bent A-form DNA and helped explain the molecular interactions contributing to specific binding. Here, we extend our previous approach^11^ and apply it to MarA and Rob, leveraging the smaller size of these transcription factors to sample binding/unbinding time scales more extensively. This permits a better understanding of transcription factor binding modes and how these are affected by mutation.

We have performed extensive all-atom MD simulations with explicit water to study the conformational dynamics of both transcription factors and their targets in the *apo* and bound states. To connect with prior experimental studies, we use the promoter sequences *micF* (*micFU*, *micFP,* and *micFA*) and *mar* (*marU* and *marP*),^23,44^ in complex with both proteins, as well as manually created loop deletion and C-terminal deletion variants of Rob in complex with both *mar* and *micF*. We have performed a cumulative 307.5 μs of MD simulations, permitting detailed insight into the recognition mechanisms and interaction differences between the two complex systems. We are able to explain why the A-box is the primary contributor to DNA-binding affinity, elucidate the molecular interactions that control this, and introduce changes to Rob that convert it between A-box-only and A-and-B-box binding. Taken cumulatively, these findings thus establish the molecular mechanism for differential binding affinity and common specificity between these two important transcription factors.

## Methods

### System Setup

Our starting point for all simulations in this work is a 2.7 Å crystal structure of Rob in complex with promoter *micF* (5′-TGACA**GCAC**TGAATGT**CAA**AG-3′) (PDB ID: 1D5Y(23,31)). In the crystal structure, two Rob monomers are associated with a single *micF* sequence in two independent complexes: one monomer forms a specific complex with the DNA at the major groove and backbone, while the other monomer is bound nonspecifically on the opposite side of the DNA, one-half turn (5–6 base pairs) away from the specific binding sites. Only the specific complex (chain **A** for Rob, chains **M**, **N** for *micF*) was chosen for the present work, to be able to start from a productive binding mode of the DNA. To prevent fraying close to the binding sites, the DNA sequence was extended from 21 base pairs to 26 base pairs at the two ends using 3DNA^45^ (5′- GTTGACA**GCAC**TGAATGT**CAA**AACAC-3′), to generate the starting structure of the Rob-*micF* complex. Subsequently, and based on the starting coordinates of the Rob-*micF* complex, the *micF* base pairs were mutated to *mar* base pairs (5′- GCCGATG**CCAC**GTTTTGC**TAA**ATCGG-3′) by using an in-house script, DNA Base Mutator, available for download from Zenodo (DOI: 10.5281/zenodo.1494296).

In this study, we focus on the *micF* and *mar* promoters, which MarA also binds. To compare the DNA binding dynamics of Rob and MarA, we built straight MarA complexes with the *mar* and *micF* promoters in their straight form (the DNA is bent in the MarA crystal structure, Figure 1A). To do so, we used protein coordinates of MarA from the crystal structure 1BL0(19,31) aligned onto the crystallographic Rob-*micF* and manually generated Rob-*mar* complexes to obtain analogous MarA-*micF* and MarA-*mar* complexes. Following from this, the complexes of Rob and MarA with each mutated promoter sequence were obtained by mutating the relevant base pairs in the wild-type complex to the relevant sequence shown in Table S1, using an in-house script, DNA base mutator, which is available for download from Zenodo at the following link: https://zenodo.org/record/1494296#.YMJcMkmSnDE. In this way, 10 mutated complexes (MarA-*micFU*, MarA-*micFP*, MarA-*micFA*, MarA-*marU*, MarA-*marP*, Rob-*micFU*, Rob-*micFP*, Rob-*micFA*, Rob-*marU*, and Rob-*marP*) were constructed. Additional simulations were performed of the MarA-*mar* complex starting from the available crystallographic conformation (PDB ID: 1BL0(19,31)), as well as two Rob-*micF* and Rob-*mar* complexes in which either (1) the acidic C-terminal domain loop was deleted, or (2) the entire C-terminal domain was deleted (thus mimicking the structure of MarA, which lacks the C-terminal domain). Finally, in the case of simulations of free MarA and free Rob, the starting conformations were directly obtained from the crystal structures by deleting the DNA, and in the case of simulations of free DNA, the starting coordinates were obtained from the protein–DNA complexes by removing the protein.

Each system was then placed into an octahedral box filled with TIP3P water molecules,^46^ with a distance of at least 8 Å from the solute to the surface of the box in each direction. The necessary number of Na^+^ and Cl^–^ counterions were then added to first neutralize the system, and then achieve a 0.15 M salt concentration, in a random scheme using addIonsRand from the LEaP module as implemented in AMBER 18^47^ (for simulation specifics per system, see Tables S3 and S4). All simulations were performed using the AMBER 18 simulation package,^47^ with the protein described using the AMBER ff14SB force field,^48^ and the DNA described using the Parmbsc1 force field.^49^ The LEaP module of AMBER 18 was used to produce the initial topology and coordinates for each system, and then the hydrogen mass repartition scheme^50^ (which involves altering the mass of hydrogen atoms to 3.024 amu) was applied using the PARMED module of AMBER 18 to generate modified topology files for subsequent MD simulations. This allows for a 4 fs step size to be used in the simulations.

The parameters used to model nonstandard nucleobases, such as **U** and **P**, were taken from the parameters for U in RNA, for Uracil and P (5-(1-propynyl-uracil)), while the missing parameters for the 1-propynyl moiety were obtained using the General Amber Force Field (GAFF).^51^ Charges for the U nucleotide were adjusted to reach neutrality, while partial charges for P were calculated at the HF/6-31G* level of theory, using Gaussian 09,^52^ and fitted using the standard restrained electrostatic potential (RESP) procedure.^53^ All force field parameters used to describe nonstandard nucleobases are provided in Table S5.

### Simulation Details

All molecular dynamics simulations were performed for each system using the same protocol, and using the CUDA version of the PMEMD module^54−56^ of the AMBER 18 simulation package.^47^ Each solvated system was first subjected to a 5000 step steepest descent minimization followed by another 5000 steps of conjugate gradient minimization, with harmonic positional restraints applied to all heavy atoms of the solute with a 5 kcal mol^–1^ Å^–2^ force constant. Subsequently, the minimized systems were gradually heated up from 5 to 300 K in 500 ps, and then equilibrated for another 500 ps under an NVT ensemble coupled by the Berendsen thermostat^57^ with a time constant of 0.5 ps, and 5 kcal mol^–1^ Å^–2^ harmonic positional restraints on all heavy atoms. The system was then further optimized for 1000 ps in an NPT ensemble (300 K, 1 atm), controlled again by the Berendsen thermostat and the Berendsen barostat^57^ using a 1 ps time constant. Finally, production runs of 2.5 μs were performed for each system, as summarized in Table S3, with configurations saved every 10 ps of each trajectory for further analysis. Production simulations were performed at constant temperature (300 K) and constant pressure (1 atm), coupled by the Langevin thermostat^58,59^ with a 2 ps coupling time, and the Monte Carlo barostat with a 1 ps time constant.^60,61^ Five independent production runs with different initial velocity assignments were performed for each system but the free DNA sequences, for which only three independent runs were performed (Table S3). Due to the use of the hydrogen mass repartition method,^50^ it was possible to use a time step of 4 fs for all the MD simulations. The SHAKE algorithm^62^ was applied to constrain all bonds involving hydrogen atoms. An 8 Å cutoff was applied to all nonbonded interactions, while the long-range electrostatic interactions is described using the particle mesh Ewald (PME) approach.^63^ A summary of all MD simulations performed in this work can be found in Tables S3 and S4.

### Distances

To better quantify the protein–DNA recognition, we calculated distances, between the protein and the DNA, calculated using PLUMED v2.5.^64^ The distances between the binding domains and the DNA major groove were simply calculated based on the center of mass of all backbone atoms of the helix of the binding domain (Lys35-Thr46 and Gln85-Phe96) and that of heavy atoms of the base pairs at the A and B box (nucleotide ID 9–10,43–44 for A box and 19–20, 33–34 for B box), using eq 1**:**
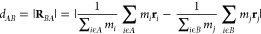
1Here, *A* and *B* are the two groups of atoms, **R***_BA_* is the vector of the center of mass of groups *A* and *B*, *m*_*i*_ and *m*_*j*_ are the masses of atoms *i* and *j* belonging to groups *A* and *B*, respectively, while **r***_i_* and **r***_j_* are the coordinates.

### Predictions of the Protein–DNA Binding Energies

Protein–DNA binding energies were estimated for six protein–DNA complexes, formed between the transcription factors MarA and Rob (both the complete Rob and the Rob C-terminal acidic-loop deletion construct studied in this work) and the promoters *mar* and *micF*. Modified topologies containing only the complex, the transcription factor or the DNA promoter were created through the PARMED module of AMBER 18.^47^ The estimation of the binding energies were then carried out using the Molecular Mechanics—Poisson–Boltzmann Surface Area (MM-PBSA) approach,^65^ using the script “mmpbsa.py”^66^ that is distributed though the AMBER 18 simulation package.^47^ The MM-PBSA calculations were carried out individually on every 50 ps of each of the five independent 2.5 μs production trajectories of each complex. For the MM-PBSA calculations, default settings were used, specifically: an ionic strength of 0.1 M, together with an internal dielectric constant of 10, in line with a previous benchmark study of protein–DNA binding^67^ (which suggested that an internal dielectric constant of as high as 10 should be used to best describe the interaction between charged protein residues and the protein-nucleic acid interface) and an external dielectric constant of 80. The resolution of the Poisson–Boltzmann grid spacing was 2.0 Å and the solvent probe radius was 1.4 Å. A maximum of 1000 interactions of the linear Poisson–Boltzmann equation was allowed.

### Principal Component Analysis

Principal component analysis (PCA) was performed using CPPTRAJ^68^ from AmberTools 18.^47^ The analysis was performed on the combined MD simulations of all MarA, Rob, and Rob-truncated complexes. This was done by first performing a rigid-body alignment to a conserved region in all complexes (residues 1–118, which share the same secondary structure) and then performing PCA on the C_α_-atoms of this region plus the phosphate atoms of the DNA backbone.

### Other Analysis

The GROMACS *do_dssp* interface to DSSP^69,70^ was used to monitor the secondary structure changes of the proteins. All the other analyses were performed using CPPTRAJ^68^ from AmberTools 18,^47^ the Visual Molecular Dynamics (VMD)^71^ package, and Bio3D.^72^ The most-populated structures were calculated by performing agglomerative root mean square deviation (RMSD) clustering using CPPTRAJ to yield 10 clusters, and taking the centroid of the most-populated cluster. All structural figures were prepared using Chimera.^32^

## Results and Discussion

### Exploring the Intrinsic Flexibility of MarA, Rob, and the DNA Sequences of Interest

**Figure 2:**
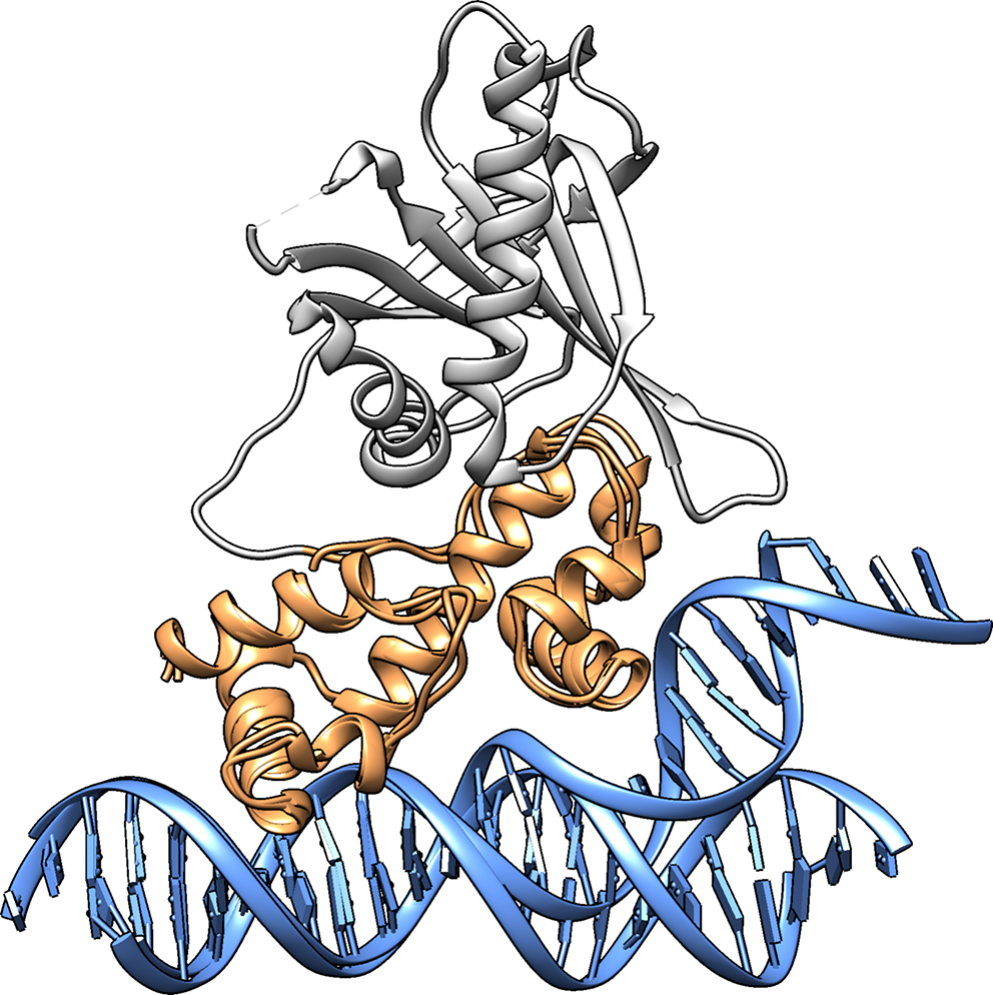
Overlay of the binding domains of MarA and Rob (PDB IDs 1BL0(19,31) and 1D5Y,^23,31^ respectively). Both binding domains are shown in tan while Rob’s extra C-terminal domain is shown in gray. As can be seen from this figure, the binding domains overlay nearly perfectly. The DNA promoters are shown in blue, with the MarA promoter (*mar*) shown in its crystallographic bent conformation toward MarA, and the Rob promoter (*micF*) shown in its crystallographic straight conformation.

The DNA-binding domains of MarA and Rob share 51% sequence identity and have an RMSD of 0.9 Å between crystallographic structures (Figure 2). Curiously, however, MarA interacts with the DNA at both the A-box and the B-box, whereas Rob only interacts with the DNA at the A-box (Figure 1), without significantly compromising the binding affinity of DNA to Rob (Table S2).^23^ We hypothesize that these two very different DNA binding modes result from differential dynamics at the binding interface, as such dynamics has been suggested to be important in other systems.^11,73,74^ These can result from differences in the intrinsic flexibility of the two proteins, sequence-dependent differences in the intrinsic flexibility of the DNA, or differences in both protein and DNA simultaneously.

It has, indeed, been shown that DNA-dynamics can play an important role in facilitating sequence-selective protein recognition,^75^ although this is not necessarily always the case, as we recently showed in the case of LacI where the intrinsic dynamics of both specific and nonspecific DNA sequences was considered to be very similar.^11^ However, the comparison to LacI may not be entirely appropriate because of differences in the bound complex. While LacI (like many DNA binding proteins^76^) bends the DNA to an upside-down “V” shape with an 36° angle upon DNA binding in a specific complex,^43^ it appears that MarA instead curves the DNA into a broad “U”-shape at a 35° angle as defined by ref (19), and therefore these proteins are interacting with the DNA differently than the interactions observed between the DNA and transcription factors such as LacI. To explore whether the intrinsic properties of either the DNA or the protein or both contribute to these difference in binding geometries and affinities, we performed microsecond time scale simulations of MarA and Rob in the absence of any DNA, as well as protein-free simulations of each of the DNA sequences of interest to this work individually, as described in the Methods and in Table S3.

**Figure 3:**
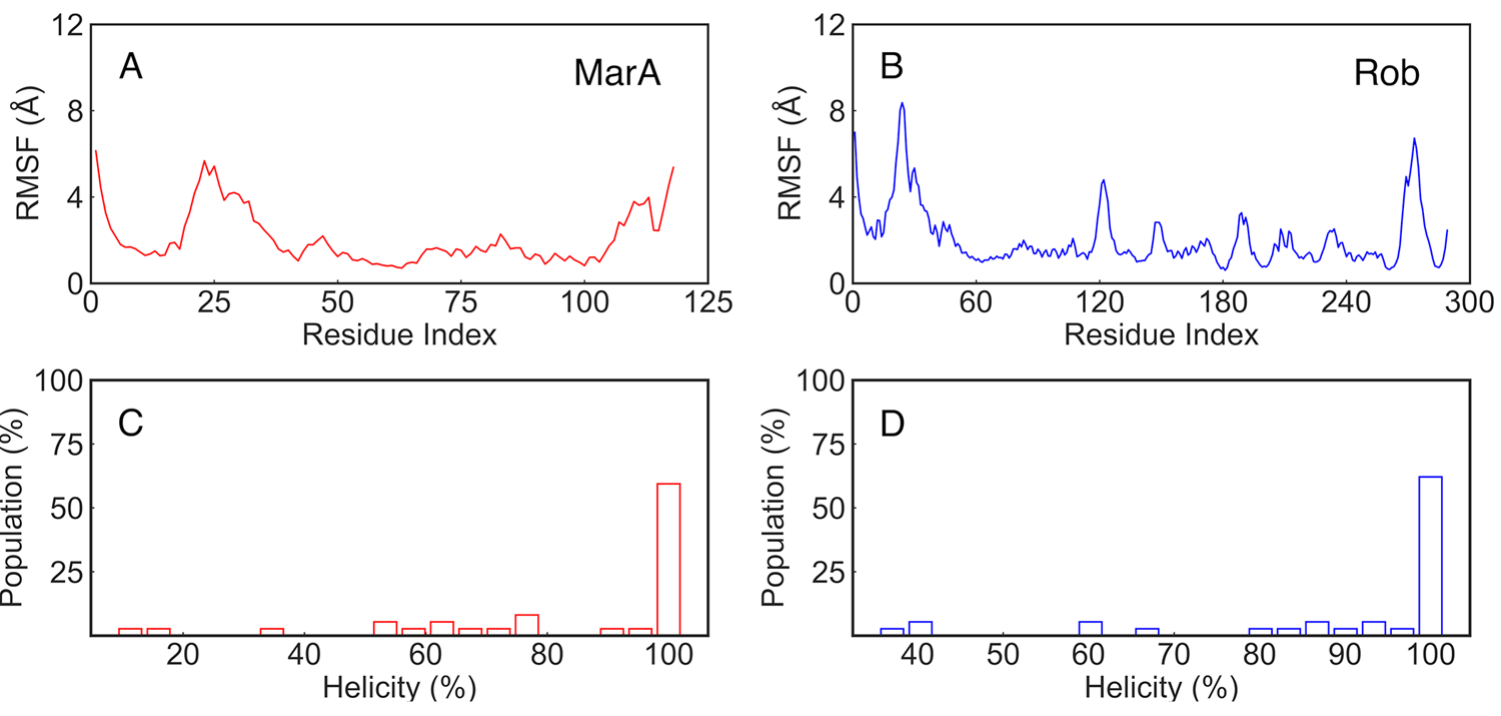
(A,B) Root mean square fluctuations (RMSF, Å) of the C_α_ atoms of the free MarA (red) and Rob (blue) proteins, respectively, calculated over five independent 2.5 μs MD simulations (Table S3). (C,D) Population distribution of the percent of helicity of the helices in the HTH motifs, calculated based on the number of residues involved in forming an α-helix or other forms of helices (π- and 3_10_-helix), over five independent 2.5 μs MD simulations of free MarA (red) and Rob (blue), respectively. A helicity of 100% is equivalent to 37 residues (Glu31-Arg36, Lys41-Thr52 in one HTH motif, and Gln91-Phe102, Pro105-Met111 in the second HTH motif) in helical form, as in the crystal structures of MarA and Rob (PDB IDs 1BL0(19,31) and 1D5Y,^23,31^ respectively). The secondary structure composition was calculated using DSSP.^69,70^

We first studied the intrinsic flexibility of both MarA and Rob (including the differences arising due to Rob’s additional C-terminal domain, Figure 1) by performing 5 × 2.5 μs MD simulations of each free protein in the absence of DNA, as described in the Methods section. The backbone root-mean square deviations (RMSD) from the reference structure range between 2 and 4 Å during the simulation time (Figure S1). From analysis of the corresponding root-mean-square fluctuations (RMSF) of the backbone C_α_-atoms of each protein, it can be seen that the flexibility of half of the N-terminal HTH motif (residues 20–35, corresponding to one helix + turn as can be seen in Figure 3) which binds to the A-box dominates the motions of both MarA and Rob (Figure 3), in agreement with the structural observation that Rob appears to preferentially bind the DNA exclusively through interactions between the N-terminal HTH motif and the A-box of the DNA (Figure 1).^23^ Additional fluctuations are observed in Rob due to the presence of the extra C-terminal domain, mainly located on loops and random coil regions. We also performed secondary structure analysis in order to evaluate the percent helicity of the DNA-binding helices in Rob and MarA in the absence of DNA, to see if these retain their structure upon DNA binding, observing that the helices remain relatively stable over the course of our simulations (Figure 3).

Following from this, we also performed 3 × 2.5 μs MD simulations of each of the *mar*, *marU*, *marP*, *micF*, *micFU*, and *micFP* DNA sequences (Tables S1 and S3). We then calculated the root-mean-square deviation (RMSD) and root-mean-square fluctuation (RMSF) of the backbone atoms (Figures S2 and S3), as well as the DNA base pair step parameters for each sequence by the procedure used in 3DNA,^77,78^ as shown in Figures S4–S11. Interestingly, Figure S11 indicates differences in the minor groove width between the *mar* and *micF* sequences (and their corresponding mutants) at a position that corresponds to the bend observed in the protein–DNA complex (Figure 1). Tying in with this, an overlay of the most-populated structures of each 500 ns time period along molecular dynamics simulations of the free *mar* and *micF* promoters (Figure S12) qualitatively indicates that while no radical bending is observed in either sequence, there is, however, seemingly a slight bend in the right-hand ∼1/3rd of the *mar* DNA fragment (Figure S12A) that is not observed in the *micF* fragment (Figure S12B). This very slight bend is consistent with the differences in the minor groove width shown in Figure S11. It also suggests that the *mar* DNA sequence must have some intrinsic bending capacity, even if this is not highly pronounced, that could rationalize the differences observed in the corresponding protein–DNA complexes. One resulting model is that unbound DNA is primarily straight, while binding to both A- and B-boxes energetically favors bent DNA. This would explain our observations.

We note here also the presence of an A-tract between the A- and B-boxes in the *mar* sequence (Figure 1 and Table S1). While it has been suggested that such A-tracts may induce helix bending and affect other structural properties,^79−82^ more recent work^83,84^ has suggested that the role of A-tracts in DNA curvature may not be as significant as previously thought. Nevertheless, it is of course possible that the A-tract present in the *mar* but not *micF* sequence contributes to the greater malleability of this sequence in the protein–DNA complex. Apart from this, we observe only minor differences between the different promoter sequences, as in our previous work on LacI.^11^ This strongly suggests that, at the intrinsic level, there are no significant differences between the wild-type and corresponding mutant sequences.

Finally, we note that, as described in the Methods section, the two terminal base pairs on each end of the sequences were omitted from the analysis to avoid well-known fraying artifacts,^85^ observed in all simulations, especially in those of *micF*, as shown in Figures S13 and S14 (and as also hinted at in the context of the greater fluctuations in the terminal base pairs as shown in the RMSF plots in Figure S3).

### Exploring the Flexibility of the Protein–DNA Interface

It is well-known from structural studies that MarA inserts both of its HTH motifs into the major grooves of the DNA duplex (referred to as the A- and B-boxes in this context, see Figure 1), thus bending the DNA upward by 35°.^19^ In contrast, Rob only inserts its N-terminal HTH motif into the A-box of the DNA duplex, while the C-terminal HTH lies on the surface of an unbent DNA duplex (Figure 1).^23^

To test the propensity of the DNA to naturally bend when in complex with MarA, we performed unrestrained conventional molecular dynamics simulations of both Rob and MarA in complex with all seven DNA sequences shown in Table S1 to equilibrate the systems (Figures S15 and S16), specifically: the *mar* and *micF* promoters, as well as five mutated DNA sequences, one with a mutation in the A-box (*micFA*) and two with mutations in the B-box (*marU*/*micFU* and *marP*/*micFP*). Note that, in the case of Rob, we observe significant motion of the B-box domain relative to the DNA, which is why the systems appear not to be fully equilibrated even after 2.5 μs of simulation time. The B-box mutations were originally developed^23^ in order to assess the importance of the B-box to protein–DNA binding. In particular, it has been suggested that the two conserved thymines within the B-boxes of the *mar* and *micF* promoters contribute to sequence specific interactions with MarA, establishing van der Waals contacts with the protein through the C5 methyl group.^19^ In the *marU* and *micFU* sequences, both thymines were replaced by uracil in order to eliminate the contacts through the C5 methyl groups, and in the *marP* and *micFP* sequences, the thymine C5 methyl groups were substituted by larger propyne groups (5–1-propynyl-uracil), in order to assess whether the C-terminal HTH motif needs to be inserted into the B-box of the DNA in order for binding to occur. It has been demonstrated that MarA and Rob both bind to these B-box mutated sequences with affinities (*K*_DNA_) similar to those of the unmodified promoters (Table S2).^23^

Following from this, we added also the *micFA* sequence presented in ref (44), which contains an A-box mutation of a central base pair (C ↔ G) in the binding domain, as it has been shown that a single (C ↔ G) substitution within the A-box of the *micF* promoter decreased the binding affinity to Rob by ∼100-fold.^44^ In this study, single base pair mutations within the A-box of the *micF* promoter in general decreased the binding affinity by 7- to 100-fold, whereas single base pair substitutions in the B-box of this promoter only had a 2- to 4-fold effect on the Rob binding affinity. This again suggests that most of the sequence specific DNA-interactions made between Rob and the *micF* promoter involve the A-box of the DNA sequence, with minimal contribution from interactions at the B-box. This agrees with the structural observation that only the N-terminal HTH motif of Rob is inserted inside the A-box of the DNA duplex (Figure 1).^23^ However, the origin of these effects remains unclear.

Our goal, therefore, was to address two distinct questions through our simulations of the MarA/Rob-DNA complexes: (1) Do the B-box mutations also disrupt MarA-DNA interactions with the B-box, or does MarA just switch interactions with neighboring base pairs? (2) Do single base pair mutations in the A-box have similar disruptive effects on MarA-DNA interactions as on Rob-DNA interactions? To achieve this, we performed 5 × 2.5 μs MD simulations of each protein in complex with all the DNA sequences considered in this work (Table S1). In the case of MarA, we initiated these simulations from a crystal structure of the protein and DNA (PDB ID: 1BL0(19,31)) superimposed on the crystal structure of Rob (PDB ID: 1D5Y(23,31)) so that only the N-terminal HTH motif is inserted inside the DNA A-box, with the second HTH motif lying on the surface of the DNA, as shown for Rob in Figure 1. The resulting complex is shown in Figure S17. This was done in order to allow free fluctuations in the interactions between MarA and the protein at both the A- and B-boxes upon modifying the DNA sequences, without biasing the simulations by starting from a crystal structure in which MarA was already interacting with both the A- and B-boxes. The crystallographic MarA-*mar* conformation (PDB ID: 1BL0(19,31)) was also simulated in order to assess the stability of the crystal structure’s bent conformation.

**Figure 4:**
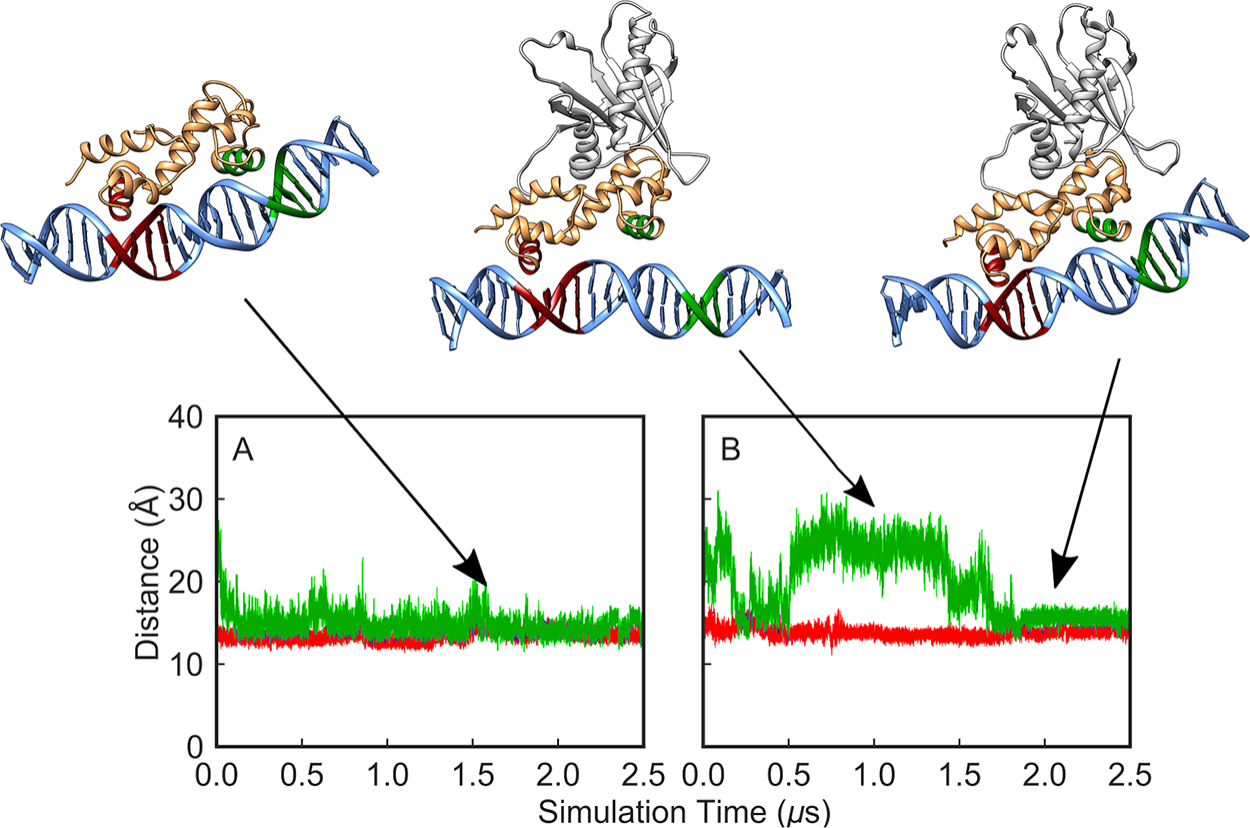
Time evolution of the distances between the helices inserted inside the major groove and the base pairs at the A- and B-boxes (shown in red and green, respectively) during 2.5 μs of MD simulation of the (A) MarA-*mar* and (B) Rob-*mar* complexes. Shown here also are examples of different binding conformations during our simulations selected based on visual examination of the trajectories.

We started by monitoring the time evolution of the insertion of both HTH motifs inside the A- and B-boxes, tracking the distances between the helices inserted inside the major groove and the base pairs at the A- and B-boxes (i.e., between Lys35-Thr46 and nucleotides 9–10 and 43–44 for the A-box and Gln85-Phe96 to nucleotides 19–20 and 33–34 for the B-box), as shown in Figures 4 and Figures S18, S19. On the basis of this data, we observe the following: (1) The MarA-*mar* bent conformation observed in the crystal structure is stable, with both HTH motifs inserted in the major groove of the *mar* promoter during the simulation time (Figure S20). (2) Even when the starting structure of MarA is not inserted in both binding domains of the *mar* promoter, the system reaches a conformation close to that observed in the crystal structure,^19^ with a DNA angle of 35°, within the first 500 ns of the simulations (Figure 4, Supplementary Movie 1; note that all Supplementary Movies were generated using morphing between simulation frames in Chimera for visual clarity, this creates artifactual floppiness of some of the DNA base pairs, which is not observed in the actual trajectories). (3) The HTH motif of Rob which appears to be sitting on the surface of the B-box in the Rob crystal structure (PDB ID: 1D5Y,^23,31^Figure 1) is actually conformationally flexible, and can go in and out of the B-box and establish quite strong interactions with the B-box, that last for more than 1 μs in some replicas (Figure 4, Supplementary Movie 2). (**4**) B-box mutations do not prevent the HTH helices of either MarA or Rob from being inserted into the major grooves of any of the DNA sequences; therefore, despite the mutations, MarA is able to bend the DNA and establish interactions with both the A- and B-boxes of the different DNA sequences.

**Figure 5:**
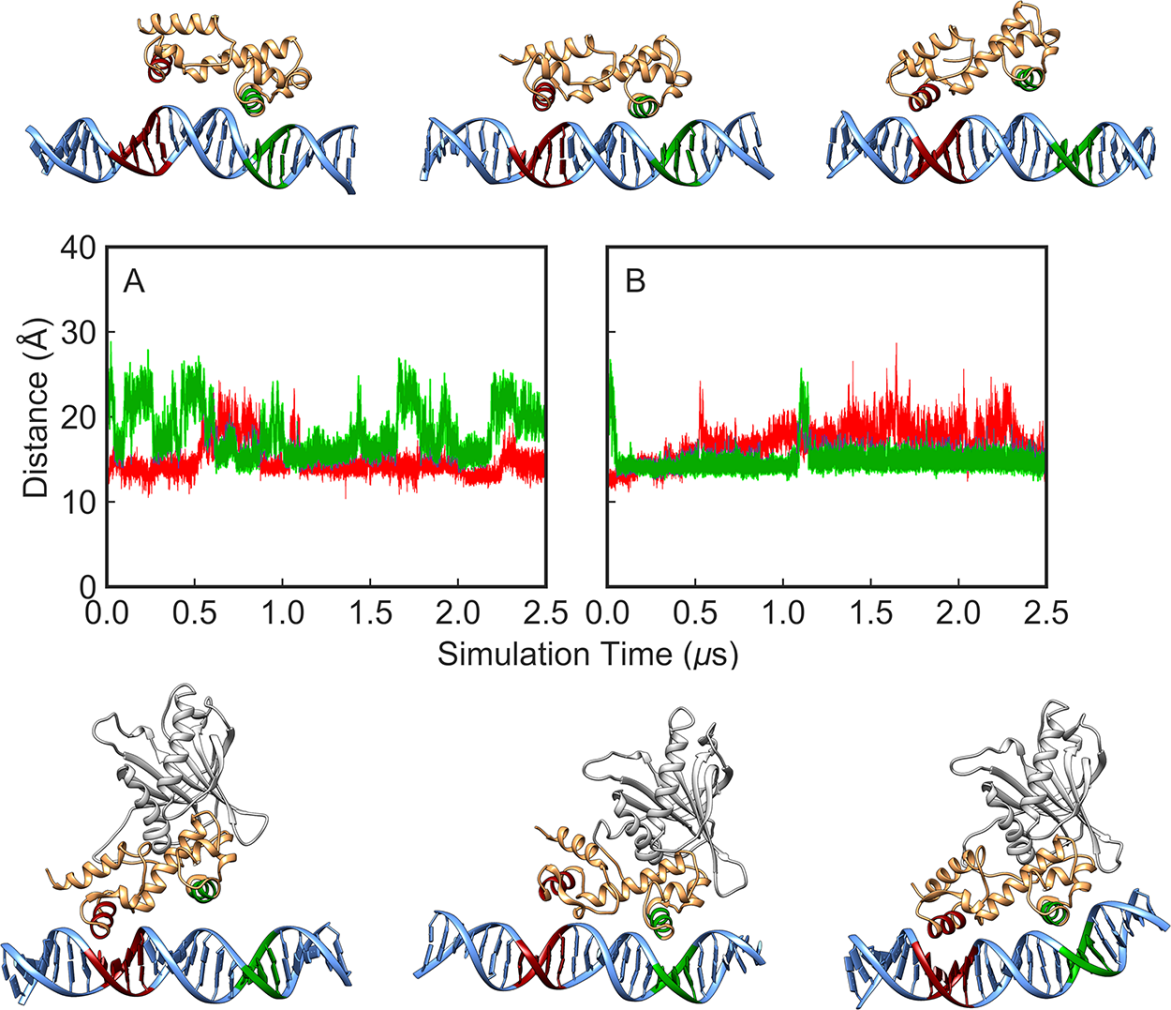
Time evolution of the distances between the helices inserted inside the major groove and the base pairs at the A- and B-boxes (shown in red and green respectively) during 2.5 μs MD simulations of (A) Rob-*micFA* and (B) MarA-*micFA* mutated complexes. Representative snapshots corresponding to different binding conformations during our simulations, showing how the A-box mutation confers more binding flexibility to both Rob and MarA, thus allowing these protein to interact with either, both or neither of the A- and B-boxes.

When examining the interactions between MarA and Rob and the A-box mutated sequence, *micFA*, it can be seen that for Rob, in some replicas, the N-terminal HTH motif initially inserted into the A-box of the DNA sequence dissociates from the DNA for more than 1 μs, allowing the second HTH motif to interact with the B-box and fit nicely inside the major groove of the DNA duplex (Figure 5, Supplementary Movie 3). This suggests that even though the key binding interactions that determine specificity appear to be between the protein and the A-box of the DNA, nevertheless, when bound to the A-box mutant sequence *micFA*, the A-box and B-box appear to be able to “trade-off” on interactions. This could also theoretically facilitate the sideways motion of Rob across the DNA sequence. For comparison, the impact of A-box mutations on MarA is even more substantial, as it causes MarA to dissociate completely from the A-box for more than 1 μs and eventually from both A- and B-boxes, instead lying on the surface of a straight DNA duplex (Figure 5 and Figure S21, and Supplementary Movie 4). This once again emphasizes the importance of the A-box for tight binding. Note that all Supplementary Movies are available for download from Zenodo, DOI: 10.5281/zenodo.4119117, and are also presented as Supporting Information.

### Interactions that Drive the Binding of MarA and Rob to Different DNA Sequences

Having confirmed through simulation that the protein–DNA interface is highly dynamic, with selectivity and tight binding being primarily driven by interactions with the A-box (Figure 1), we next set out to explore what key interactions facilitate the binding of MarA and Rob to the different DNA sequences considered in this work. As mentioned above, from Table S2, it can be seen that mutations in the B-box have very little effect on *K*_DNA_(23) (the thermodynamic differences are too small to be captured computationally), whereas in contrast from ref (44), it is known that the (C ↔ G) substitution within the A-box of the *micF* promoter decreases the binding affinity to Rob by ∼100-fold (corresponding to ∼3 kcal mol^–1^ difference in binding affinity). This is in qualitative agreement with our simulations, which show a tendency of MarA to dissociate completely from the *micFA* sequence during the simulation time (Figure 5).

**Figure 6:**
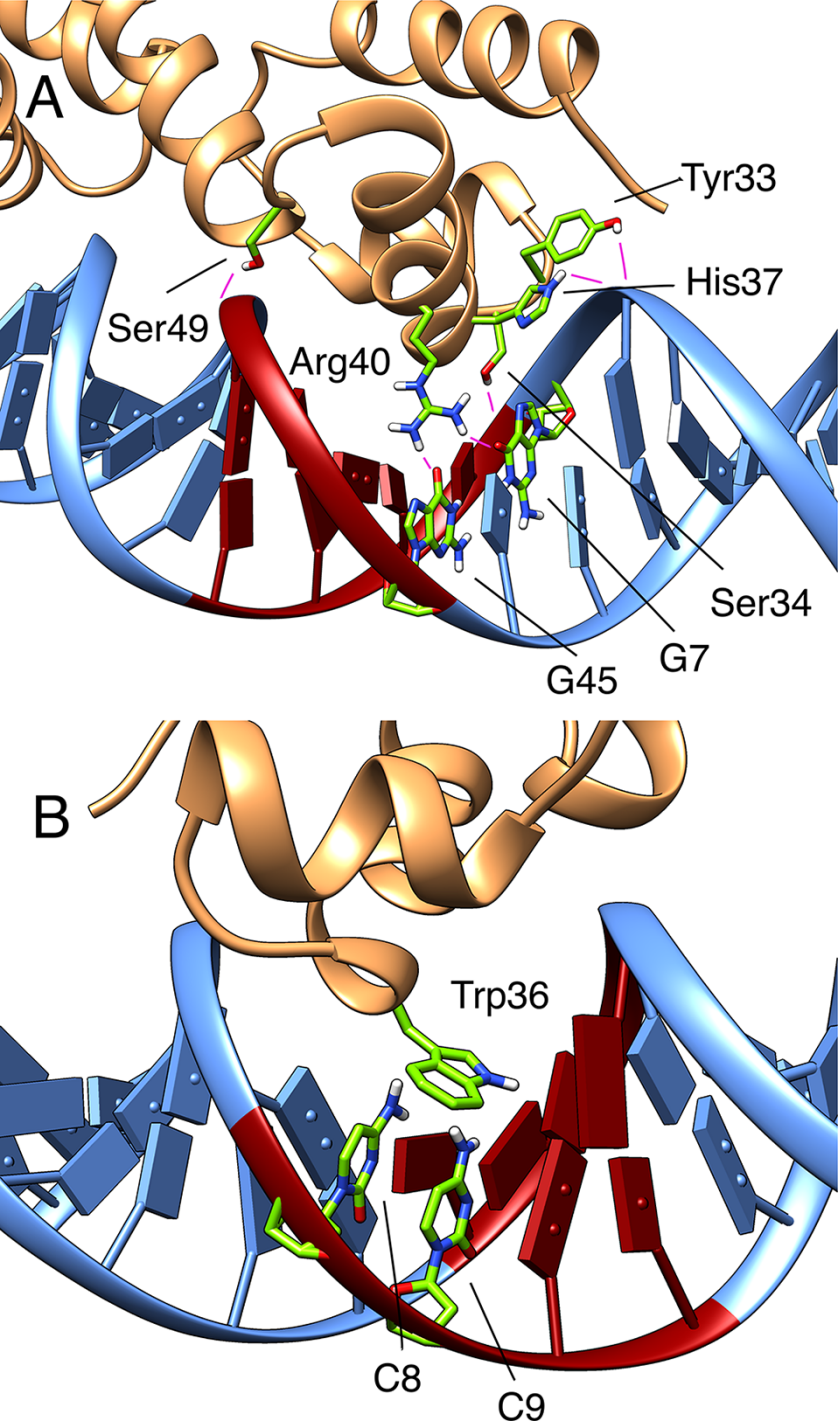
Key residues of the N-terminal HTH motif of MarA that form (A) stable hydrogen bonds or (B) π–π stacking interactions with the A-box of *mar* promoter during our simulations. Structures were selected based on visual examination of the trajectories.

To further understand the molecular interactions that are likely driving A- and B-box binding, we performed a detailed analysis of the hydrogen bonds formed between the helices inserted inside A- and B-boxes of the different DNA sequences (Tables S6 to S13). Most stable hydrogen bonds are established nonspecifically between the protein and DNA backbone atoms (Figure 6). As expected, A-box hydrogen bonds are more stable than B-box hydrogen bonds for both the MarA and Rob complexes (Tables S6–S7 and S10–S11); however, while the stability of A-box interactions is similar between MarA and Rob complexes, the stability of B-box interactions is drastically reduced in Rob complexes due to the observed dynamic movement of the B-box. Interestingly, the stability of such A-box interactions slightly raises within *mar* and *micF* mutated sequences in MarA complexes, while it is maintained within all Rob complexes. These trends are in line with the observed differences in *K*_DNA_ within MarA and Rob mutated sequences.^23^

Here we identified two key arginine residues that drive specific hydrogen bonding interactions within the A- and B-boxes. Arginine 40 is inserted in the A-box of both MarA and Rob complexes, establishing specific hydrogen bonds with the oxygen atom of guanine nucleobases 7, 44, and 45 in *mar* sequences, or 8 and 44 in *micF* sequences (Figure 6A and Tables S8 and S12, for base pair numbering see Table S1). In all the *micF* complexes, the interaction Arg40-G8 is much more stable than the others, being the one showing major impact, in *micFA* complexes. Arg90 is inserted in the B-box, establishing specific hydrogen bonds with the oxygen atom of guanine nucleobases 17 and 35 and the free oxygen atom of thymine nucleobase 16 in *mar* sequences, or guanine nucleobases 17 and 34 and thymine nucleobases 16 and 18 in *micF* sequences (Tables S9 and S13). Such B-box specific interactions are less prevalent along all our Rob-DNA simulations due to the dynamic movement of B-box in Rob complexes. More nonspecific interactions were also observed between this Arg90 and the backbone atoms of the B-box, highlighting a shallow insertion of this arginine inside B-box, as well as the overall minor impact of B-box mutations on the *K*_DNA_.^23^ This is significant as while protein–DNA backbone interactions are important for the stability of the protein–DNA complex, they may also aid in orienting the protein toward the DNA thus allowing for specific interactions with the DNA base edges. These are in turn crucial to specificity and selective target binding.^86−88^

Apart from specific hydrogen bonds, other specific interactions are formed between Trp36 and cytosines 8 and 9 in the *mar*-based sequences, or guanine 8 and cytosine 9 in the *micF*-based sequences, through T-shaped π–π stacking interactions (Figure 6B). Such interactions are stable throughout the simulation for both the *mar* and *micF* promoters and the corresponding B-box mutated sequences, with an average distance between ring centroids (*R*_c_) of ∼5.5 ± 0.5 Å and an angle between normal vectors of each ring plane (γ) of ∼68.5 ± 11.8° (Table S14). Such T-shaped π–π stacking interactions between tryptophan and nucleobases of an HTH motif inserted in the major groove of a recognition sequence were observed before in transpose S911 from the IS3 family of bacterial insertion sequences, and were reported to have a crucial impact on DNA binding.^89^ Furthermore, Gillette et al. reported a decrease of 20–40% in an *in vivo* assay where β-galactosidase expression is activated by a W36A MarA variant binding a *micF* promoter. In contrast, when the same variant bound the *mar* promoter the activation was unaffected.^35^ Taliaferro et al. also reported a decrease in the *in vivo* β-galactosidase expression (37%) activated by a W36A Rob variant binding the *micF* promoter, as well as an even more pronounced effect when binding a promoter where the corresponding interacting cytosine (C9) was mutated to thymine (11%).^37^ We observe that when introducing an A-box mutation (*micFA*), such π–π stacking interactions are disrupted, as shown on Table S14 and Supplementary Movie 4, due to the dissociation of the A-box preventing the insertion of both Arg40 and Trp36 inside the major groove.

Finally, water-mediated interactions, including hydrogen bonding, may play an additional role in determining the binding stabilities of Rob and MarA to different DNA sequences. Such interactions can be probed experimentally with optical tweezers^90^ and force spectroscopy.^91^ In addition, it has been suggested that bridging waters play a key energetic role in protein–DNA interactions.^92^ To obtain computational insight into the overall protein–water, DNA–water and protein–water–DNA interactions in each system, we first identified all crystallographic protein/DNA–water interactions in the crystal structure of the MarA-*mar* complex^19,31^ and analyzed the stability of the corresponding interactions in our simulated complexes (Tables S15–S17). An interaction was defined as a hydrogen bond if the distance between the oxygen atom of the water molecule and a donor or acceptor on the protein/DNA fell within 3.5 Å. Note that, as described in the Methods, our simulations of MarA in complex with different sequences were initiated from a straight conformation of the DNA to see if we observe the crystallographically observed bending in our simulations, and therefore crystallographic water molecules were not retained in our simulations. However, we identified and tracked the analogous water molecules. From the data shown in Tables S15 and S16, it can be seen that most of the protein–water or DNA–water interactions observed in the crystal structure are well-conserved in our simulations of MarA with different sequences, with a few interactions that are only present less than half the simulation time. However, the sole bridging protein–water–DNA interaction observed in the crystal structure, between the side chain of Thr89, a water molecule, and T/U32, is rarely observed in our simulations.

In the case of Rob, as there are no water molecules present in the Rob-*micF* crystal structure,^23,31^ we cannot compare directly to crystallographic water molecules. However, we have examined all protein–water–DNA bridging interactions found for more than 10% of the simulation time for each system (Tables S18–S21). We observe that, with the exception of an interaction in the A-box of MarA and Rob (Gly32:G/A7), all bridging interactions are transient, and are present less than half the simulation time. In addition, bridging water molecules found in the vicinity of the A-box are shown to be more stable over simulation time than those in the B-box for all systems studied.

The largest differences between the systems appear in the B-box and, specifically, when comparing full-length Rob to the Rob loop-deletion and C-terminal deletion complexes. Specifically, for full-length Rob, the majority of the more stable protein–water–DNA bridging interactions established in the B-box are between residues of the C-terminal HTH motif and nucleobases 16–18 of either the *mar* or *micF* promoters. In contrast, in full-length Rob, more transient bridging interactions are established between the HTH motif and nucleobases from the other side of the B-box, as would be expected due to the in-and-out movement of the DNA at the B-box of the protein. In the truncated systems, however, the water-bridging interactions established between the C-terminal HTH motif and nucleobases 31–33, found on the farthest side of the box, are shown to be much more stable during the simulation time (∼40% of the simulation time), thus assisting the insertion of the transcription factor into the B-box.

### Impact of Protein–DNA Complexation on the Structural Flexibility of the DNA

As a further point of interest, we have explored the impact of protein–DNA interactions on the flexibility of the different DNA sequences considered in this work. Here, we started by comparing the sequence-dependent DNA deformability of the base pair step parameters both in the absence of protein and when the different DNA sequences shown in Table S1 are in complex with MarA and Rob. In the case of the free DNA sequences (Figures S4 to S11), we obtained similar average values of the base pair step parameters for all DNA sequences, that are in turn in good overall agreement with the reference configuration of B-DNA (−0.02 ± 0.45, −0.23 ± 0.81, 3.32 ± 0.19 Å for shift, slide, and rise, and −0.1° ± 2.5, 0.6° ± 5.2 and 36° ± 6.8 for tilt, roll, and twist angles, respectively).^78^ However, when the DNA forms a complex with MarA or Rob, the base pair step parameters are slightly affected, particularly in the A-box where the protein–DNA interactions are more stable (Figures S22 to S37). In addition, we note the impact of protein–DNA complexation on the roll angle at the base pair steps between both binding boxes of both transcription factors in all sequences studied here (Figures S30 and S31). The change in this angle is an indicator of induced curvature at this region. This effect is especially notable within the MarA complexes, in which the DNA is stably bound at both the A- and B-boxes, and which in turn bends the DNA in order to allow interaction with both regions of the protein.

We also analyzed the major and minor groove widths of the different DNA sequences both in the presence and absence of protein, and while the major groove widths oscillate around the values expected for the canonical B-DNA form of DNA (14.2 Å),^78^ in the protein DNA complexes, the minor groove widths adjacent to both binding domains of the proteins are reduced to ∼4 Å due to the overall bending of the DNA strand upon binding to the protein (Figures S38 and S39), with conformational differences in the free DNA sequences that may be linked to the presence of the A-tract in the *mar* but not *micF* sequences (Figure 1 and Table S1). This observed compression appears to be critical in order to allow the two recognition helices of Rob and MarA to be able to insert into the adjacent major grooves upon DNA binding. To confirm this, we selected the base pair steps with higher changes in the minor groove width, and analyzed their time evolution (Figures S40 and S41). From this data, it can be seen that in the MarA complexes, where the protein binds equally efficiently to both the A- and B-boxes of the DNA, the minor groove widths for GC/AT (*mar* based sequences) and TA/TA (*micF* based sequences) oscillate around 3 Å, indicating a bent structure such as that shown in Figure 1. In contrast, in the case of Rob–DNA complexes, where the C-terminal HTH motif binds and unbinds from the B-box of the DNA, the minor groove widths of the base pair steps that correspond to the region between the two different binding domains oscillate either around 3 Å when Rob interacts with both the A- and B-boxes of the DNA (bent DNA), or around 5–6 Å (close to the canonical B-DNA value of 6.2 Å)^78^ when the binding domain of Rob dissociates from the DNA.

### Impact of Protein–DNA Complexation on the Structural Dynamics of MarA and Rob

We have also explored the impact of protein–DNA interactions on the structural integrity and dynamical behavior of MarA and Rob. In the absence of DNA (Figure 3) both MarA and Rob show reduced helicity and higher RMSF (and thus structural integrity) of the HTH motifs compared to when DNA is present (Figure S42) and the helices are inserted into the major groove. It is perhaps unsurprising that these HTH motifs would be more rigid and helical when inserted into the major grooves of the DNA helices, in particular the A-box of the DNA duplexes (nucleotides 28–40).

**Figure 7:**
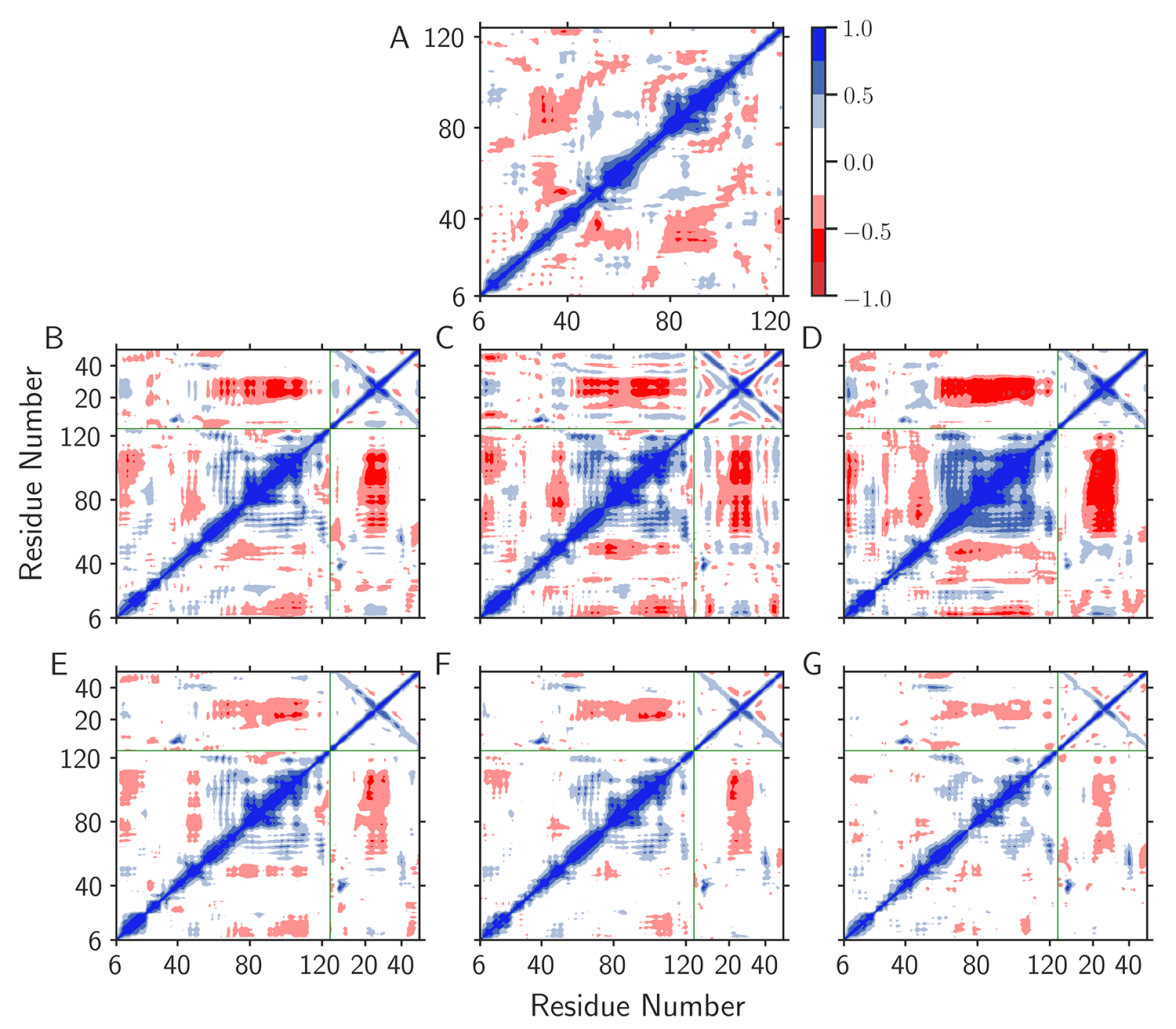
Calculated DCCM plots based on simulations of (A) free MarA, as well as MarA in complex with the DNA promoters (B) *mar*, (C) *marU*, (D) *marP*, (E) *micF*, (F) *micFU*, or (G) *micFP*. The plots were calculated with Bio3D^72^ by considering only the C_α_- and P atoms during 5 × 2 μs simulations each of free MarA and the relevant MarA-DNA complexes, respectively.

As our previous study of LacI indicated the presence of sequence-dependent patterns of (anti)correlated motions upon DNA binding,^11^ we performed similar analysis of MarA and Rob, based on dynamic cross-correlation maps (DCCM) generated using Bio3D.^72^ The data for MarA is shown in Figure 7, and the analogous data for Rob is shown in Figure S43. In the case of the free proteins, there is very little correlation in the motion of the different protein regions, although there appears to be a small amount of anticorrelation in the motion of the two HTH motifs, in particular on the more flexible turn that links both helices (residues 27–37 and 87–97), suggesting these helices move in opposite directions to each other in an anticorrelated fashion. Analogous motions are not observed in free Rob, and are also lost in the MarA DNA complexes.

As in the case of LacI,^11^ we observe a significantly greater presence of (anti-) correlated motions in both MarA and Rob once these proteins form complexes with DNA, and while we observe more or less the same correlation patterns with all sequences studied (Table S1), the intensity of the correlations appears to vary in a sequence-dependent fashion. In the case of MarA (Figure 7), we observe high anticorrelation between the region containing the C-terminal HTH motif, the region connecting both HTH motifs, and the half of the DNA duplex containing the B-box, indicating movement of the C-terminal HTH helix in and out of the B-box. This is particularly pronounced in the case of the native *mar* promoter sequence and its associated mutants. Curiously, in the case of Rob (Figure S43) the corresponding regions display *less* anticorrelated motions than in MarA, which is likely a consequence of the increased size of Rob, which in turn could be constraining the motion of the C-terminal domain.

### The Role of the Rob C-Terminal Domain in DNA Binding

Finally, we have focused on explaining the observed differences between the DNA-binding modes of MarA and Rob, including the inability of Rob to establish stable interactions with the B-box of any of the DNA sequences studied in this work, even though they possess the same conserved binding domains. We have in particular focused on the dynamical behavior of the extra C-terminal domain of Rob, to understand why, while we do observe interactions with both the A-box and B-box of DNA simultaneously during our simulations, these interactions are only transient. To explore the origins of this effect, we first performed additional MD simulations on two artificial Rob-*micF* and Rob-*mar* complexes for which the extra C-terminal domain was deleted allowing this artificially truncated Rob to structurally mimic MarA (the truncated structure) (Figure S44). The remaining N-terminal binding domain of Rob has 51% sequence identity with MarA,^23^ as well as high structural similarity (RMSD of 0.9 Å between the two binding domains). Therefore, unsurprisingly, our simulations (Figures S45 and S46) show that removing the C-terminal domain of Rob allows Rob to mimic the MarA binding mode.

**Figure 8:**
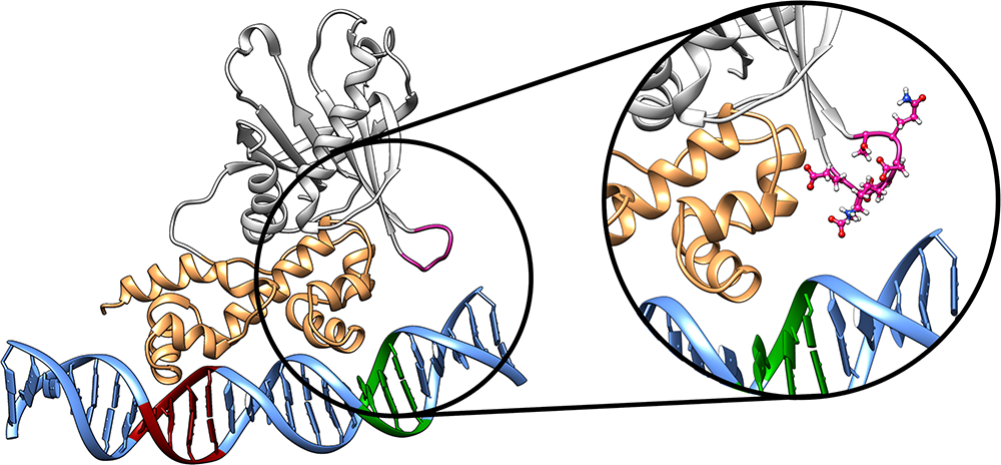
Snapshot of the Rob-*micF* complex extracted from our simulations. As can be seen from Figure 1, in the initial crystal structure, Rob interacts with the DNA exclusively through the A-box. Shown here is the conformation of the system when the DNA is bent toward the protein, thus establishing transient B-box interactions with the C-terminal HTH motif. The acidic loop (residues 187–193) connecting strands β3 and β4 in the extra C-terminal domain are highlighted in pink. It has been proposed that this loop sterically hinders stable interactions between wild-type Rob and the B-box.^23^

Interestingly, the extra C-terminal domain of Rob contains an acidic loop (residues 187–193) connecting strands β3 and β4. This loop is located close to the DNA binding surface when the DNA is bent in a similar manner as in the MarA-DNA complex (Figure 8, loop highlighted in pink). It was originally suggested that the presence of the acidic loop highlighted in Figure 8 might be preventing the DNA from bending toward Rob,^23^ but this hypothesis was discarded when it was observed that a loop-deleted variant of Rob shows similar affinity toward the *micF* promoter as the wild-type does.^23^ Here, in addition to our artificial Rob C-terminal deletion constructs, we have also constructed artificial Rob-*mar* and Rob-*micF* loop-deletion complexes, in which residues 187–193 were substituted by an alanine connecting strands β3 and β4.

**Figure 9:**
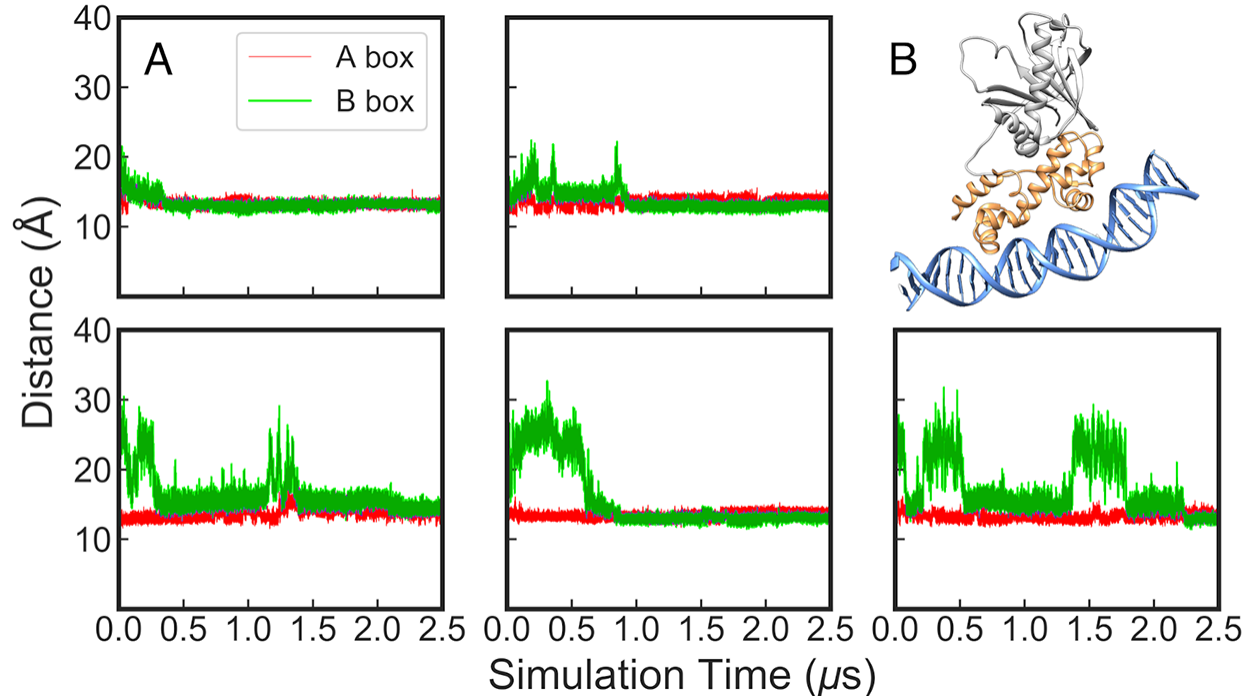
(A) Time evolution of the distances between the helices inserted inside the major groove and the base pairs at the A- and B-box during 5 × 2.5 μs simulations of the Rob loop-deletion variant in complex with the *micF* promoter. The distance analysis was performed using PLUMED v2.5^64^ based on snapshots extracted every 10 ps of the simulations. (B) An example of a structure from the simulation of the Rob loop-deletion complex showing the stable bent conformation of the DNA sequences toward the protein.

We have performed MD simulations on these systems using the same protocol as for other variants studied in this work. Our simulation data shows that the loop-deletion construct is in fact capable of bending both the *mar* and *micF* promoters and establishing stable interactions between the B-box of the DNA and the C-terminal HTH motif (Figures 9 and S47). In particular, the distances observed in the Rob loop-deletion complex are similar to those observed in all MarA-DNA complexes, indicating that truncation of this loop allows this Rob variant to stably bind both the A-box and B-box simultaneously (Figure 9), in contrast to wild-type Rob (Figure 4 and Figure S19).

This is further supported by principal-component analysis of alpha-carbon displacements across the simulation data set to identify modes that differentiate transcription factors. In this analysis, we observe the first principal component (PC1) to describe the majority of the variance (76%) between the different structures (Figure S48); however, the motion across this PC appears to be nonspecific. The second, PC2, contributes 8% to the total variance, and describes the binding and unbinding of the DNA from the B-box of the different systems, as shown from a projection of this PC onto representative structures of Rob-*mar*, loop-deletion Rob-*mar*, and MarA-*mar* (Figure S49), as well as analysis of the distances between the C-terminal HTH motif and the base pairs at the B-box along PC2 for each system (Figure S50). This data shows that while DNA binding and unbinding are observed in simulations of full-length Rob, the truncated system behaves more similarly to MarA.

**Figure 10:**
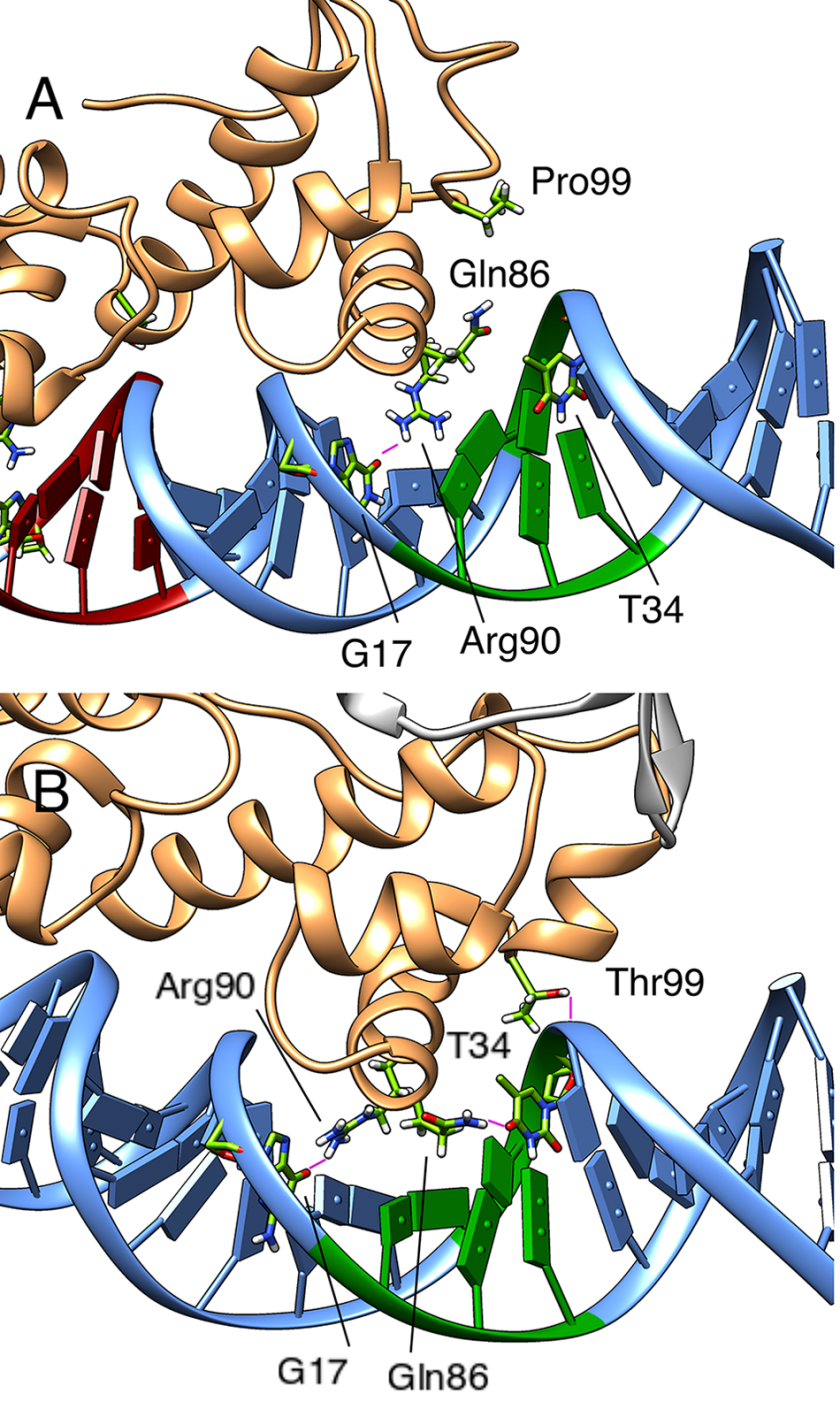
Key residues of the C-terminal HTH motif of (A) MarA and (B) Rob loop-deleted variant, that form stable hydrogen bonds interactions with the B-box of the *mar* promoter during our simulations. Snapshots were selected based on visual examination of the trajectories.

To understand the structural basis for this effect, we have compared the protein–DNA hydrogen bonding interactions established between MarA and Rob and the respective promoter sequences in both the full (Tables S6–S13) and truncated Rob constructs. This analysis shows that while both the specific and nonspecific interactions at the A-box are basically conserved in all systems, as expected, we observe more stable protein–DNA interactions at the B-box in the truncated Rob-DNA complexes than in the full Rob-DNA complex. However, when comparing the MarA B-box protein–DNA interactions and the truncated Rob B-box protein–DNA interactions (Tables S7 and S11), we observe a significant increase in B-box interactions between the Rob Thr99 side chain and the DNA (the corresponding residue in MarA is a proline that does not establish any hydrogen-bonding interactions with the DNA, see Figure 10). An additional interaction appears to be formed between the side chains of Gln86 and the oxygen of the T34 nucleobase in the B-box of the DNA in the C-terminal domain truncated Rob simulations (Table S11), that is not observed in the corresponding MarA simulations (Figure 10).

Because our simulations predict that deleting the C-terminal acidic loop in Rob converts its DNA binding mode to be more similar to MarA, we evaluated the binding electrostatics of Rob, its loop deletion variant, and MarA to both *mar* and *micF* promoter sequences. Binding energies were estimated using MM/PBSA calculations^65^ on snapshots taken at 50 ps intervals from five 2.5-μs simulations of each of these six complexes (90 μs aggregate simulation time, Figures S51 and S52). These calculations predict that the loop-deletion variant can take on at least two states, which are also predicted for wild-type Rob but at slightly different relative probabilities (Figure S51). We note the poor quantitative agreement between our predictions and the *K*_DNA_ values presented in Table S2. It is unclear what the origin of this effect is, although it is possible that a dielectric constant of 10, which was selected based on prior work,^67^ is too low to accurately describe the interaction between the protein and the highly charged DNA molecule, which would be expected to have a much higher local dielectric constant (see discussion of protein dielectric constants in, for example, ref (93)). In addition, the approximate nature of single-structure MM/PBSA calculations and the slow equilibration between states preclude accurate estimation of the equilibrium binding affinities and relative fractions of each state present at equilibrium, but if the first of these two states predominates at equilibrium, this would be consistent with the report of Ellenberger and colleagues that deleting the acidic loop did not noticeably change binding affinity.^23^

We note here that, as there have been suggestions in the literature that the nonlinear version of the Poisson–Boltzmann equation provides a better description of electrostatic potential calculations of highly charged biomolecules such as DNA (see for example, refs (67, 94, and 95) among others), we have also performed a comparison of the polar contribution to the solvation free energy calculated using both the linear and nonlinear Poisson–Boltzmann equations as implemented in the Delphi PBE solver^96,97^ (Table S22). In these calculations, we detect a significant (*p* = 0.02 via the Wilcoxon rank sum statistic) but extremely small difference (<1 kcal mol^–1^) in energies between the two approaches. However, both linear and nonlinear Poisson–Boltzmann calculations are often insufficient to describe the electrostatic potential for challenging systems such as highly charged protein–DNA complexes in a meaningful way. Since robust methods to estimate the absolute free energy differences between the bound and unbound states are not computationally tractable for these systems at the present time, simplified approximations such as those used in the Poisson–Boltzmann approach provide a reasonable proxy. However, the highly qualitative nature of these results should be taken into consideration.

Thus, our simulations indicate that while the factors contributing to the protein–DNA binding affinity in the acidic-loop truncated RobA construct are likely complex, from a structural perspective, truncation of the C-terminal domain allows for Rob to assume a “MarA-like” DNA binding mode (Figure 1), and, indeed, truncation of the acidic loop appears to be sufficient to facilitate this interconversion between the two binding modes. Therefore, it is likely that the single A-box binding mode observed in Rob plays some form of regulatory role for this protein, that is made feasible by the fact that Rob can bind the promoter sequence at only the A-box without significantly compromising DNA binding affinity.

## Conclusions

In the present study, we have performed a detailed μs-time scale simulation study of the transcription factors MarA and Rob in complex with both their native promoter sequences, and a variety of mutated DNA sequences based on their native promoter sequences (Table S1). The small system size of these proteins (∼100 amino acids in the case of MarA and ∼180 amino acids in the case of Rob) makes these proteins tractable for long time scale simulations, making them excellent model systems with which to explore the molecular details of protein–DNA recognition. In parallel, the involvement of their corresponding regulons in the development of multidrug^18^ resistance makes them particularly important systems to study from a biomedical perspective, as they are important targets for antimicrobial drug development.

Our simulations explore the structural and dynamical properties of both free MarA and Rob, as well as both proteins in complex with different DNA sequences with mutations in both the A-box and the B-box of the DNA sequences (Figure 1 and Table S1). We briefly summarize the insights gained from our analyses below.

Our analysis of the flexibility and DNA base pair steps of the free DNA sequences (Figures S2 to S11) indicates subtle differences in the minor groove width between the *mar* and *micF* sequences (and corresponding variants) that might potentially be linked to differences in the intrinsic ability of these sequences to bend when in complex with either MarA or Rob. This could, in part, be due to the presence of an A-tract between the A- and B-boxes of the *mar* sequence (Figure 1 and Table S1), as it has been suggested that such A-tracts may contribute to DNA deformability.^79−82^

Following from this, we demonstrate that the binding domains (HTH motifs) of both MarA and Rob are highly flexible in the absence of DNA but become more ordered upon DNA binding. In agreement with structural information, from our distance analyses (Figure 9 and Figures S18 to S21, and S45 to S47), we observe stable interactions of both binding domains of MarA with both the A-box and the B-box of the DNA in all systems studied, whereas in the case of Rob, we observe only transient interactions with the B-box with the exception of an artificially constructed loop-deletion variant in which we remove an acidic C-terminal loop that has been proposed to prevent binding of Rob to the B-box of the DNA through a steric clash^23^ (although this hypothesis was discarded due to the similar binding affinities of both full and truncated Rob toward the *micF* promoter sequence^23^). Our simulations indicate that removal of this loop allows for bending of the DNA, and thus facilitates interactions with both the A-box and B-boxes simultaneously. Our analyses of DNA base pair steps (Figures S22–S41), and in particular the changes in minor groove width (Figures S36 and S37) indicate that the specific B-box binding behavior is due to DNA bending, rather than a change in transcription factor conformation. This is supported by our principal component analysis of bound-state motions (Figures S48–S50) where we again observe transient interactions between the HTH motif of full-length Rob and the B-box, which are stable in corresponding simulations of the Rob loop-deletion variant. In addition, our RMSF and helicity (Figure S42) analyses indicate a decrease in flexibility of both HTH motifs, as well as a population shift toward increased helicity of these motifs, which would aid in DNA binding.

We also observe sequence-dependent changes in the dynamics and correlated motions of both proteins upon complexation with DNA (Figure 7 and Figure S43), and in general, modification of the A-box of the DNA duplexes appears to have more drastic consequences for protein–DNA binding (both structural and electrostatic) than substitutions introduced into the B-box. H-bond analysis of these simulations (Tables S6–S13) indicate that a greater number of hydrogen bonding interactions are established between the HTH motifs of both transcription factors and the DNA, with a reduction in these interactions between Rob and the B-box of the DNA. While the majority of these A-box interactions are nonspecific, our simulations showcase the role of Arg40 of the N-terminal HTH motif in allowing for tight specific binding through interactions with G7/8 of the DNA duplex, as well as a stable T-shaped π–π stacking interaction between the side chain of Trp36 and nucleobases 8 and 9 of the DNA. Both interactions are lost, however, in the A-box mutant *micFA* (Table S14), likely contributing to the significantly impaired *K*_DNA_ toward this mutant (Table S2).

Finally, simulations of truncated versions (acidic-loop and C-terminal deletion variants) of Rob in complex with both *mar* and *micF* (Figure 9 and Figures S45–S47) indicate a shift in binding mode from stable A-box/transient B-box binding in full-length Rob to stable binding of the HTH domains to both the A- and B-boxes in the truncated variants, similarly to the binding mode observed in MarA (Figure 1). This further highlights the role of the acidic-loop and the HTH motifs in driving DNA recognition.

Taken together, our simulations support a critical role for interactions between the N-terminal HTH-motif and the A-box of the DNA for facilitating DNA binding and recognition, with the B-box being less critical, and potentially mainly facilitating sidewise motion of MarA and Rob on the DNA while searching for their promoter sequences (see the Supplementary Movies). This detailed molecular insight into DNA binding and recognition by MarA and Rob provides an important step forward toward the efficient design of antivirulence agents targeting these proteins.

## Supporting Information Available

Additional simulation analysis, including base pair steps, flexibility, root-mean-square deviations, and further analysis of key distances and hydrogen bonding interactions (PDF)
Movie 1 Conformational dynamics of the MarA-mar complex. The MarA-mar complex, starting from the B-box unbound conformation mimicking the Rob-micF crystal structure (PDB ID: 1d5y), rapidly inserts the second HTH motif into the B-box as found in the MarA-mar crystal structure (PDB ID: 1bl0). The DNA and transcription factor are shown as cartoons, and the key residues in the A- (Arg40 and Trp36) and B-boxes (Arg90) are represented by green sticks (MP4)
Movie 2 Conformational dynamics of Rob-micF complex. Conformational flexibility of the Rob-micF complex with the HTH motif of Rob sitting on the surface of the B-box in the crystal structure (PDB ID: 1d5y), showing a dynamical in and out movement at the B-box region. The DNA and transcription factor are shown as cartoons, and the key residues in the A- (Arg40 and Trp36) and B-boxes (Arg90) are represented by green sticks (MP4)
Movie 3 Conformational dynamics of the Rob-micFA complex. The A-box mutation promotes Rob unbinding from the A-box, and "trades off" interactions with the B-box. The DNA and transcription factor are shown as cartoons, and the key residues in A- (Arg40 and Trp36) and B-boxes (Arg90) are represented by green sticks (MP4)
Movie 4 Conformational dynamics of the MarA-micFA complex. The A-box mutation promotes MarA unbinding from the A-box, with the transcription factor eventually unbinding from both boxes and lying on the surface of the DNA. The DNA and transcription factor are shown as cartoons, and the key residues in A- (Arg40 and Trp36) and B-boxes (Arg90) are represented by green sticks (MP4)

## References

[ref1] Latchman D. S. Transcription Factors: An Overview. Int. J. Exp. Pathol. 1993, 74, 417–422. 8217775 PMC2002184

[ref2] Siggers T.; Gordan R. Protein-DNA Binding: Complexities and Multi-Protein codes. Nucleic Acids Res. 2014, 42, 2099–2111. 10.1093/nar/gkt1112. 24243859 PMC3936734

[ref3] Hudson W. H.; Ortlund E. A. The Structure, Function and Evolution of Proteins that Bind DNA and RNA. Nat. Rev. Mol. Cell Biol. 2014, 15, 749–760. 10.1038/nrm3884. 25269475 PMC4280011

[ref4] Smith N. C.; Matthews J. M. Mechanisms of DNA-Binding Specificity and Functional Gene Regulation by Transcription Factors. Curr. Opin. Struct. Biol. 2016, 38, 68–74. 10.1016/j.sbi.2016.05.006. 27295424

[ref5] Vicente M.; Chater K. F.; De Lorenzo V. Bacterial Transcription Factors Involved in Global Regulation. Mol. Microbiol. 1999, 33, 8–17. 10.1046/j.1365-2958.1999.01445.x. 10411719

[ref6] Balleza E.; López-Bojorquez L. N.; Martínez-Antonio A.; Resendis-Antonio O.; Lozada-Chávez I.; Balderas-Martínez Y. I.; Encarnación S.; Collado-Vides J. Regulation by Transcription Factors in Bacteria: Beyond Description. FEMS Microbiol. Rev. 2009, 33, 133–151. 10.1111/j.1574-6976.2008.00145.x. 19076632 PMC2704942

[ref7] Ishihama A. Prokaryotic Genome Regulation: Multifactor Promoters, Multitarget Regulators and Hierarchic Networks. FEMS Microbiol. Rev. 2010, 34, 628–645. 10.1111/j.1574-6976.2010.00227.x. 20491932

[ref8] Halford S. E.; Marko J. F. How Do Site-Specific DNA-Binding Proteins Find Their Targets?. Nucleic Acids Res. 2004, 32, 3040–3052. 10.1093/nar/gkh624. 15178741 PMC434431

[ref9] Marklund E.; Amselem E.; Kipper K.; Zheng X.; Johansson M.; Deindl S.; Elf J. Direct Observation of Rotation-Coupled Protein Diffusion Along DNA on the Microsecond Timescale. BioRxiv 2018, 401414 10.1101/401414.

[ref10] Jones D. L.; Leroy P.; Unoson C.; Fange D.; Ćurić V.; Lawson M. J.; Elf J. Kinetics of dCas9 Target Search in *Escherichia coli*. Science 2017, 357, 1420–1424. 10.1126/science.aah7084. 28963258 PMC6150439

[ref11] Liao Q.; Lüking M.; Krüger D. M.; Deindl S.; Elf J.; Kasson P. M.; Kamerlin S. C. L. Long Time-Scale Atomistic Simulations of the Structure and Dynamics of Transcription Factor-DNA Recognition. J. Phys. Chem. B 2019, 123, 3576–3590. 10.1021/acs.jpcb.8b12363. 30952192

[ref12] Marklund E. G.; Mahmutovic A.; Berg O. G.; Hammar P.; van der Spoel D.; Fange D.; Elf J. Transcription-Factor Binding and Sliding on DNA Studied Using Micro- and Macroscopic models. Proc. Natl. Acad. Sci. U. S. A. 2013, 110, 19796–19801. 10.1073/pnas.1307905110. 24222688 PMC3856812

[ref13] Mondal A.; Bhattacherjee A. Searching Target Sites on DNA by Proteins: Role of DNA Dynamics Under Confinement. Nucleic Acids Res. 2015, 43, 9176–9186. 10.1093/nar/gkv931. 26400158 PMC4627088

[ref14] Tobes R.; Ramos J. L. AraC-XylS Database: A Family of Positive Transcriptional Regulators in Bacteria. Nucleic Acids Res. 2002, 30, 318–321. 10.1093/nar/30.1.318. 11752325 PMC99111

[ref15] Martin R. G.; Rosner J. L. Genomics of the MarA/SoxS/Rob Regulon of *Escherichia Coli*: Identification of Directly Activated Promoters by Application of Molecular Genetics and Informatics to Microarray Data. Mol. Microbiol. 2002, 44, 1611–1624. 10.1046/j.1365-2958.2002.02985.x. 12067348

[ref16] Neidhardt F. C.; Savageau M. A. Regulation Beyond the Operon; American Society for Microbiology Press: Washington, DC, 1996; Vol. 1.

[ref17] Duval V.; Lister I. M. MarA, SoxS and Rob of *Escherichia coli* - Global Regulators of Multidrug Resistance, Virulence and Stress rResponse. Int. J. Biotechnol. Wellness Ind. 2013, 2, 101–124. 10.6000/1927-3037.2013.02.03.2. 24860636 PMC4031692

[ref18] Sharma P.; Haycocks J. R. J.; Middlemiss A. D.; Kettles R. A.; Sellars L. E.; Ricci V.; Piddock L. J. V.; Grainger D. C. The Multiple Antibiotic Resistance Operon of Enteric Bacteria Controls DNA Repair and Outer Membrane Integrity. Nat. Commun. 2017, 8, 1444–1456. 10.1038/s41467-017-01405-7. 29133912 PMC5684230

[ref19] Rhee S.; Martin R. G.; Rosner J. L.; Davies D. R. A Novel DNA-Binding Motif in MarA: The First Structure for an AraC Family Transcriptional Activator. Proc. Natl. Acad. Sci. U. S. A. 1998, 95, 10413–10418. 10.1073/pnas.95.18.10413. 9724717 PMC27908

[ref20] Gallegos M. T.; Schleif R.; Bairoch A.; Hofmann K.; Ramos J. L. Arac/XylS Family of Transcriptional Regulators. Microbiol. Mol. Biol. Rev. 1997, 61, 393–410. 10.1128/.61.4.393-410.1997. 9409145 PMC232617

[ref21] Ramos J. L.; Rojo F.; Zhou L.; Timmis K. N. A Family of Positive Regulators Related to the *Pseudomonas Putida* TOL Plasmid XylS and the *Escherichia coli* AraC Activators. Nucleic Acids Res. 1990, 18, 2149–2152. 10.1093/nar/18.8.2149. 2186376 PMC330695

[ref22] Gallegos M. T.; Michan C.; Ramos J. L. The XylS/AraC Family of Regulators. Nucleic Acids Res. 1993, 21, 807–10. 10.1093/nar/21.4.807. 8451183 PMC309210

[ref23] Kwon H. J.; Bennik M. H.; Demple B.; Ellenberger T. Crystal Structure of the *Escherichia coli* Rob Transcription Factor in Complex with DNA. Nat. Struct. Biol. 2000, 7, 424–430. 10.1038/75213. 10802742

[ref24] Demple B. Redox Signaling and Gene Control in the *Escherichia Coli* SoxRS Oxidative Stress Regulon - A Review. Gene 1996, 179, 53–57. 10.1016/S0378-1119(96)00329-0. 8955629

[ref25] Cohen S. P.; Hachler H.; Levy S. B. Genetic and Functional Analysis of the Multiple Antibiotic Resistance (Mar) Locus in *Escherichia Coli*. J. Bacteriol. 1993, 175, 1484–1492. 10.1128/jb.175.5.1484-1492.1993. 8383113 PMC193236

[ref26] Ariza R. R.; Li Z.; Ringstad N.; Demple B. Activation of Multiple Antibiotic Resistance and Binding of Stress-Inducible Promoters by *Escherichia Coli* Rob Protein. J. Bacteriol. 1995, 177, 1655–1661. 10.1128/jb.177.7.1655-1661.1995. 7896685 PMC176790

[ref27] Ariza R. R.; Cohen S. P.; Bachhawat N.; Levy S. B.; Demple B. Repressor Mutations in the MarRAB Operon that Activate Oxidative Stress Genes and Multiple Antibiotic Resistance in *Escherichia Coli*. J. Bacteriol. 1994, 176, 143–148. 10.1128/JB.176.1.143-148.1994. 8282690 PMC205025

[ref28] Greenberg J. T.; Chou J. H.; Monach P. A.; Demple B. Activation of Oxidative Stress Genes by Mutations at the SoxQ/CfxB/MarA locus of *Escherichia Coli*. J. Bacteriol. 1991, 173, 4433–4439. 10.1128/JB.173.14.4433-4439.1991. 1648558 PMC208106

[ref29] Jair K. W.; Yu X.; Skarstad K.; Thony B.; Fujita N.; Ishihama A.; Wolf R. E. Jr. Transcriptional Activation of Promoters of the Superoxide and Multiple Antibiotic Resistance Regulons by Rob, A Binding Protein of the *Escherichia Coli* Origin of Chromosomal Replication. J. Bacteriol. 1996, 178, 2507–2513. 10.1128/jb.178.9.2507-2513.1996. 8626315 PMC177972

[ref30] White D. G.; Goldman J. D.; Demple B.; Levy S. B. Role of the AcrAB Locus in Organic Solvent Tolerance Mediated by Expression of MarA, SoxS, or RobA in *Escherichia Coli*. J. Bacteriol. 1997, 179, 6122–6126. 10.1128/JB.179.19.6122-6126.1997. 9324261 PMC179517

[ref31] Berman H. M.; Westbrook J.; Feng Z.; Gilliland G.; Bhat T. N.; Weissig H.; Shindyalov I. N.; Bourne P. E. The Protein Data Bank. Nucleic Acids Res. 2000, 28, 235–242. 10.1093/nar/28.1.235. 10592235 PMC102472

[ref32] Pettersen E. F.; Goddard T. D.; Huang C. C.; Couch G. S.; Greenblatt D. M.; Meng E. C.; Ferrin T. E. UCSF Chimera - A Visualization System for Exploratory Research and Analysis. J. Comput. Chem. 2004, 25, 1605–1612. 10.1002/jcc.20084. 15264254

[ref33] Harrison S. C. A Structural Taxonomy of DNA-Binding Domains. Nature 1991, 353, 715–719. 10.1038/353715a0. 1944532

[ref34] Brennan R. G.; Matthews B. W. The Helix-Turn-Helix DNA Binding Motif. J. Biol. Chem. 1989, 264, 1903–1906. 10.1016/S0021-9258(18)94115-3. 2644244

[ref35] Gillette W. K.; Martin R. G.; Rosner J. L. Probing the *Escherichia Coli* Transcriptional Activator MarA Using Alanine-Scanning Mutagenesis: Residues Important for DNA Binding and Activation. J. Mol. Biol. 2000, 299, 1245–1255. 10.1006/jmbi.2000.3827. 10873449

[ref36] Dangi B.; Gronenborn A. M.; Rosner J. L.; Martin R. G. Versatility of the Carboxy-Terminal Domain of the Alpha Subunit of RNA Polymerase in Transcriptional Activation: Use of the DNA Contact Site as a Protein Contact Site for MarA. Mol. Microbiol. 2004, 54, 45–59. 10.1111/j.1365-2958.2004.04250.x. 15458404

[ref37] Taliaferro L. P.; Keen E. F. 3rd; Sanchez-Alberola N.; Wolf R. E. Jr. Transcription Activation by *Escherichia Coli* Rob at Class II Promoters: Protein-Protein Interactions Between Rob’s N-Terminal Domain and The Sigma(70) Subunit of RNA Polymerase. J. Mol. Biol. 2012, 419, 139–157. 10.1016/j.jmb.2012.03.019. 22465792 PMC3640845

[ref38] Zhang C.; Chen S.; Bai X.; Dedkova L. M.; Hecht S. M. Alteration of Transcriptional Regulator Rob *In Vivo*: Enhancement of Promoter DNA Binding and Antibiotic Resistance in the Presence of Nucleobase Amino Acids. Biochemistry 2020, 59, 1217–1220. 10.1021/acs.biochem.0c00103. 32157864 PMC7175798

[ref39] Zhang C.; Chen S.; Dedkova L. M.; Hecht S. M. Effects of Nucleobase Amino Acids on the Binding of Rob to Its Promoter DNA: Differential Alteration of DNA Affinity and Phenotype. Biochemistry 2020, 59, 2111–2119. 10.1021/acs.biochem.0c00290. 32412234 PMC7301883

[ref40] Pérez A.; Luque F. J.; Orozco M. Frontiers in Molecular Dynamics Simulations of DNA. Acc. Chem. Res. 2012, 45, 196–205. 10.1021/ar2001217. 21830782

[ref41] van der Vaart A. Coupled Binding–Bending–Folding: The Complex Conformational Dynamics of Protein-DNA Binding Studied by Atomistic Molecular Dynamics Simulations. Biochim. Biophys. Acta, Gen. Subj. 2015, 1850, 1091–1098. 10.1016/j.bbagen.2014.08.009. 25161164

[ref42] Yoo J.; Winogradoff D.; Aksimentiev A. Molecular Dynamics Simulations of DNA-DNA and DNA-Protein Interactions. Curr. Opin. Struct. Biol. 2020, 64, 88–96. 10.1016/j.sbi.2020.06.007. 32682257

[ref43] Kalodimos C. G.; Bonvin A. M. J. J.; Salinas R. K.; Wechselberger R.; Boelens R.; Kaptein R. Plasticity in Protein–DNA Recognition: Lac Repressor Interacts With Its Natural Operator O1 Through Alternative Conformations of Its DNA-Binding Domain. EMBO J. 2002, 21, 2866–2876. 10.1093/emboj/cdf318. 12065400 PMC126071

[ref44] Li Z.; Demple B. Sequence Specificity for DNA Binding by *Escherichia Coli* SoxS and Rob Proteins. Mol. Microbiol. 1996, 20, 937–945. 10.1111/j.1365-2958.1996.tb02535.x. 8809747

[ref45] Zheng G.; Lu X. J.; Olson W. K. Web 3DNA - A Web Server for the Analysis, Reconstruction, and Visualization of Three-Dimensional Nucleic-Acid Structures. Nucleic Acids Res. 2009, 37, W240–W246. 10.1093/nar/gkp358. 19474339 PMC2703980

[ref46] Jorgensen W. L.; Chandrasekhar J.; Madura J. D.; Impey R. W.; Klein M. L. Comparison of Simple Potential Functions for Simulating Liquid Water. J. Chem. Phys. 1983, 79, 926–935. 10.1063/1.445869.

[ref47] Case D. A.; Ben-Shalom I. Y.; Brozell S. R.; Cerutti D. S.; Cheatham T. E. III; Cruzeiro V. W. D.; Darden T. A.; Duke R. E.; Gilson M. K.; Gohlke H.; AMBER 2018; University of California: San Francisco, 2018.

[ref48] Maier J. A.; Martinez C.; Kasavajhala K.; Wickstrom L.; Hauser K. E.; Simmerling C. ff14SB: Improving the Accuracy of Protein Side Chain and Backbone Parameters from ff99SB. J. Chem. Theory Comput. 2015, 11, 3696–3713. 10.1021/acs.jctc.5b00255. 26574453 PMC4821407

[ref49] Ivani I.; Dans P. D.; Noy A.; Perez A.; Faustino I.; Hospital A.; Walther J.; Andrio P.; Goni R.; Balaceanu A.; Portella G.; Battistini F.; Gelpi J. L.; Gonzalez C.; Vendruscolo M.; Laughton C. A.; Harris S. A.; Case D. A.; Orozco M. Parmbsc1: A Refined Force Field for DNA Simulations. Nat. Methods 2016, 13, 55–58. 10.1038/nmeth.3658. 26569599 PMC4700514

[ref50] Hopkins C. W.; Le Grand S.; Walker R. C.; Roitberg A. E. Long-Time-Step Molecular Dynamics Through Hydrogen Mass Repartitioning. J. Chem. Theory Comput. 2015, 11, 1864–1874. 10.1021/ct5010406. 26574392

[ref51] Wang J.; Wolf R. M.; Caldwell J. W.; Kollman P. A.; Case D. A. Development and Testing of a General Amber Force Field. J. Comput. Chem. 2004, 25, 1157–1174. 10.1002/jcc.20035. 15116359

[ref52] Frisch M. J.; Trucks G. W.; Schlegel H. B.; Scuseria G. E.; Robb M. A.; Cheeseman J. R.; Scalmani G.; Barone V.; Mennucci B.; Petersson G. A.; Nakatsuji H.; Caricato M.; Li X.; Hratchian H. P.; Izmaylov A. F.; Bloino J.; Zheng G.; Sonnenberg J. L.; Hada M.; Ehara M.; Toyota K.; Fukuda R.; Hasegawa J.; Ishida M.; Nakajima T.; Honda Y.; Kitao O.; Nakai H.; Vreven T.; Montgomery J. A. Jr.; Peralta J. E.; Ogliaro F.; Bearpark M.; Heyd J. J.; Brothers E.; Kudin K. N.; Staroverov V. N.; Kobayashi R.; Normand J.; Raghavachari K.; Rendell A.; Burant J. C.; Iyengar S. S.; Tomasi J.; Cossi M.; Rega N.; Millam J. M.; Klene M.; Knox J. E.; Cross J. B.; Bakken V.; Adamo C.; Jaramillo J.; Gomperts R.; Stratmann R. E.; Yazyev O.; Austin A. J.; Cammi R.; Pomelli C.; Ochterski J. W.; Martin R. L.; Morokuma K.; Zakrzewski V. G.; Voth G. A.; Salvador P.; Dannenberg J. J.; Dapprich S.; Daniels A. D.; Farkas O.; Foresman J. B.; Ortiz J. V.; Cioslowski J.; Fox D. J. Gaussian 09, Rev. D.01; Gaussian Inc.: Wallingford, CT, 2009.

[ref53] Bayly C. I.; Cieplak P.; Cornell W.; Kollman P. A. A Well-Behaved Electrostatic Potential Based Method Using Charge Restraints for Deriving Atomic Charges: The RESP Model. J. Phys. Chem. 1993, 97, 10269–10280. 10.1021/j100142a004.

[ref54] Gotz A. W.; Williamson M. J.; Xu D.; Poole D.; Le Grand S.; Walker R. C. Routine Microsecond Molecular Dynamics Simulations with AMBER on GPUs. 1. Generalized Born. J. Chem. Theory Comput. 2012, 8, 1542–1555. 10.1021/ct200909j. 22582031 PMC3348677

[ref55] Le Grand S.; Götz A. W.; Walker R. C. SPFP: Speed Without Compromise - A Mixed Model for GPU Accelerated Molecular Dynamics Simulations. Comput. Phys. Commun. 2013, 184, 374–380. 10.1016/j.cpc.2012.09.022.

[ref56] Salomon-Ferrer R.; Gotz A. W.; Poole D.; Le Grand S.; Walker R. C. Routine Microsecond Molecular Dynamics Simulations with AMBER on GPUs. 2. Explicit Solvent Particle Mesh Ewald. J. Chem. Theory Comput. 2013, 9, 3878–3888. 10.1021/ct400314y. 26592383

[ref57] Berendsen H. J. C.; Postma J. P. M.; Van Gunsteren W. F.; Dinola A.; Haak J. R. Molecular Dynamics with Coupling to an External Bath. J. Chem. Phys. 1984, 81, 3684–3690. 10.1063/1.448118.

[ref58] Pastor R. W.; Brooks B. R.; Szabo A. An Analysis of the Accuracy of Langevin and Molecular Dynamics Algorithms. Mol. Phys. 1988, 65, 1409–1419. 10.1080/00268978800101881.

[ref59] Loncharich R. J.; Brooks B. R.; Pastor R. W. Langevin Dynamics of Peptides: The Frictional Dependence of Isomerization Rates of N-Acetylalanyl-N′-Methylamide. Biopolymers 1992, 32, 523–535. 10.1002/bip.360320508. 1515543

[ref60] Allen M. P.; Tildesley D. J. Computer Simulation of Liquids; Oxford, Science: Great Britain, 1987.

[ref61] Åqvist J.; Wennerstrom P.; Nervall M.; Bjelic S.; Brandsdal B. O. Molecular Dynamics Simulations of Water and Biomolecules with a Monte Carlo Constant Pressure Algorithm. Chem. Phys. Lett. 2004, 384, 288–294. 10.1016/j.cplett.2003.12.039.

[ref62] Ryckaert J. P.; Ciccotti G.; Berendsen H. J. C. Numerical Integration of the Cartesian Equations of Motion of a System with Constraints: Molecular Dynamics of n-Alkanes. J. Comput. Phys. 1977, 23, 327–341. 10.1016/0021-9991(77)90098-5.

[ref63] Darden T.; York D.; Pedersen L. Particle Mesh Ewald: An NLog(N) Method for Ewald Sums in Large Systems. J. Chem. Phys. 1993, 98, 10089–10092. 10.1063/1.464397.

[ref64] Tribello G. A.; Bonomi M.; Branduardi D.; Camilloni C.; Bussi G. PLUMED 2: New Feathers for an Old ird. Comput. Phys. Commun. 2014, 185, 604–613. 10.1016/j.cpc.2013.09.018.

[ref65] Kollman P. A.; Massova I.; Reyes C.; Kuhn B.; Huo S.; Chong L.; Lee M.; Lee T.; Duan Y.; Wang W.; et al. Calculating Structures and Free Energies of Complex Molecules: Combining Molecular Mechanics and Continuum Models. Acc. Chem. Res. 2000, 33, 889–897. 10.1021/ar000033j. 11123888

[ref66] Miller B. R. III; McGee T. D. Jr.; Swails J. M.; Homeyer N.; Gohlke H.; Roitberg A. E. MMPBSA.py: An Efficient Program for End-State Free Energy Calculations. J. Chem. Theory Comput. 2012, 8, 3314–3321. 10.1021/ct300418h. 26605738

[ref67] Ramos R. M.; Moreira I. S. Computational Alanine Scanning Mutagenesis - An Improved Methodological Approach for Protein–DNA Complexes. J. Chem. Theory Comput. 2013, 9, 4243–4256. 10.1021/ct400387r. 26592413

[ref68] Roe D. R.; Cheatham T. E. 3rd PTRAJ and CPPTRAJ: Software for Processing and Analysis of Molecular Dynamics Trajectory Data. J. Chem. Theory Comput. 2013, 9, 3084–3095. 10.1021/ct400341p. 26583988

[ref69] Kabsch W.; Sander C. Dictionary of Protein Secondary Structure: Pattern Recognition of Hydrogen-Bonded and Geometrical Features. Biopolymers 1983, 22, 2577–637. 10.1002/bip.360221211. 6667333

[ref70] Touw W. G.; Baakman C.; Black J.; te Beek T. A. H.; Krieger E.; Joosten R. P.; Vriend G. A Series of PDB-Related Databanks for Everyday Needs. Nucleic Acids Res. 2015, 43, D364–D368. 10.1093/nar/gku1028. 25352545 PMC4383885

[ref71] Humphrey W.; Dalke A.; Schulten K. VMD: Visual Molecular Dynamics. J. Mol. Graphics 1996, 14, 33–8. 10.1016/0263-7855(96)00018-5. 8744570

[ref72] Skjaerven L.; Yao X. Q.; Scarabelli G.; Grant B. J. Integrating Protein Structural Dynamics and Evolutionary Analysis with Bio3D. BMC Bioinf. 2014, 15, 399 10.1186/s12859-014-0399-6. PMC4279791 25491031

[ref73] Chu X.; Liu F.; Maxwell B. A.; Wang Y.; Suo Z.; Wang H.; Han W.; Wang J. Dynamic Conformational Change Regulates the Protein-DNA Recognition: An Investigation on Binding of a Y-Family Polymerase to its Target DNA. PLoS Comput. Biol. 2014, 10, e1003804 10.1371/journal.pcbi.1003804. 25188490 PMC4154647

[ref74] Etheve L.; Martin J.; Lavery R. Dynamics and Recognition Within a Protein-DNA Complex: A Molecular Dynamics Study of the SKN-1/DNA Interaction. Nucleic Acids Res. 2016, 44, 1440–1448. 10.1093/nar/gkv1511. 26721385 PMC4756839

[ref75] Battistini F.; Hospital A.; Buitrago D.; Gallego D.; Dans P. D.; Gelpi J. L.; Orozco M. How B-DNA Dynamics Decipher Sequence-Selective Protein Recognition. J. Mol. Biol. 2019, 431, 3845–3859. 10.1016/j.jmb.2019.07.021. 31325439

[ref76] Harteis S.; Schneider S. Making the Bend: DNA Tertiary Structure and Protein-DNA Interactions. Int. J. Mol. Sci. 2014, 15, 12335–12363. 10.3390/ijms150712335. 25026169 PMC4139847

[ref77] Babcock M. S.; Pednault E. P. D.; Olson W. K. Nucleic-Acid StructureAanalysis - Mathematics for Local Cartesian and Helical Structure Parameters That are Truly Comparable Between Structure. J. Mol. Biol. 1994, 237, 125–156. 10.1006/jmbi.1994.1213. 8133513

[ref78] Olson W. K.; Bansal M.; Burley S. K.; Dickerson R. E.; Gerstein M.; Harvey S. C.; Heinemann U.; Lu X. J.; Neidle S.; Shakked Z.; et al. A Standard Reference Frame for the Description of Nucleic Acid Base-Pair Geometry. J. Mol. Biol. 2001, 313, 229–237. 10.1006/jmbi.2001.4987. 11601858

[ref79] Hagerman P. J. Sequence-Directed Curvature of DNA. Annu. Rev. Biochem. 1990, 59, 755–781. 10.1146/annurev.bi.59.070190.003543. 2197990

[ref80] Haran T. E.; Mohanty U. The Unique Structure of A-Tracts and Intrinsic DNA Bending. Q. Rev. Biophys. 2009, 42, 41–81. 10.1017/S0033583509004752. 19508739

[ref81] Peters J. P.; Maher L. J. I. DNA Curvature and Flexibility *In Vitro* and *In Vivo*. Q. Rev. Biophys. 2010, 43, 23–63. 10.1017/S0033583510000077. 20478077 PMC4190679

[ref82] Lankas F.; Spackova N.; Moakher M.; Enkhbayar P.; Sponer J. A Measure of Bending in Nucleic Acid Structures Applied to A-Tract DNA. Nucleic Acids Res. 2010, 38, 3414–3422. 10.1093/nar/gkq001. 20123729 PMC2879501

[ref83] Dršata T.; Špačková N.; Jurečka P.; Zgarbová M.; Šponer J.; Lankaš F. Mechanical Properties of Symmetric and Assymetric DNA A-Tracts: Implications for Looping and Nucleosome Positioning. Nucleic Acids Res. 2014, 42, 7383–7394. 10.1093/nar/gku338. 24829460 PMC4066768

[ref84] Mills A.; Gago F. Atomistic Insight Into Sequence-Directed DNA Bending and Minicircle Formation Propensity in the Absence and Presence of Phased A-Tracts. J. Comput.-Aided Mol. Des. 2020, 34, 253–265. 10.1007/s10822-020-00288-z. 31950463

[ref85] Zgarbova M.; Otyepka M.; Sponer J.; Lankas F.; Jurecka P. Base Pair Fraying in Molecular Dynamics Simulations of DNA and RNA. J. Chem. Theory Comput. 2014, 10, 3177–3189. 10.1021/ct500120v. 26588288

[ref86] Siggers T. W.; Silkov A.; Honig B. Structural Alignment of Protein-DNA Interfaces: Insights Into the Determinants of Binding Specificity. J. Mol. Biol. 2005, 345, 1027–1045. 10.1016/j.jmb.2004.11.010. 15644202

[ref87] Pabo C. O.; Sauer R. T. Transcription Factors: Structural Families and Principles of DNA Recognition. Annu. Rev. Biochem. 1992, 61, 1053–1095. 10.1146/annurev.bi.61.070192.005201. 1497306

[ref88] Luscombe N. M.; Laskowski R. A.; Thornton J. M. Amino Acid-Base Interactions: A Three-Dimensional Analysis of Protein-DNA Interactions at An Atomic Level. Nucleic Acids Res. 2001, 29, 2860–2874. 10.1093/nar/29.13.2860. 11433033 PMC55782

[ref89] Rousseau P.; Gueguen E.; Duval-Valentin G.; Chandler M. The Helix–Turn–Helix Motif of Bacterial Insertion Sequence IS911 Transposase is Required for DNA Binding. Nucleic Acids Res. 2004, 32, 1335–1344. 10.1093/nar/gkh276. 14981152 PMC390272

[ref90] Bockelmann U.; Thomen P.; Essevaz-Roulet B.; Viasnoff V.; Heslot F. Unzipping DNA with Optical Tweezers: High Sequence Sensitivity and Force Flips. Biophys. J. 2002, 82, 1537–1553. 10.1016/S0006-3495(02)75506-9. 11867467 PMC1301953

[ref91] Bustamante C.; Bryant Z.; Smith S. B. Ten Years of Tension: Single-Molecule DNA Mechanics. Nature 2003, 421, 423–427. 10.1038/nature01405. 12540915

[ref92] Spyrakis F.; Cozzini P.; Bertoli C.; Marabotti A.; Kellogg G. E.; Mozzarelli A. Energetics of the Protein-DNA-Water Interaction. BMC Struct. Biol. 2007, 7, 4 10.1186/1472-6807-7-4. 17214883 PMC1781455

[ref93] Warshel A.; Åqvist J. Electrostatic Energy and Macromolecular Function. Annu. Rev. Biophys. Biophys. Chem. 1991, 20, 267–298. 10.1146/annurev.bb.20.060191.001411. 1714279

[ref94] Rocchia W.; Alexov E.; Honig B. Extending the Applicability of the Nonlinear Poisson–Boltzmann Equation: Multiple Dielectric Constants and Multivalent Ions. J. Phys. Chem. B 2001, 105, 6507–6514. 10.1021/jp010454y.

[ref95] Bertonati C.; Honig B.; Alexov E. Poisson-Boltzmann Calculations of Nonspecific Salt Effects on Protein-Protein Binding Free Energies. Biophys. J. 2007, 92, 1891–1899. 10.1529/biophysj.106.092122. 17208980 PMC1861767

[ref96] Li L.; Li C.; Sarkar S.; Zhang J.; Witham S.; Zhang Z.; Wang L.; Smith N. C.; Petukh M.; Alexov E. DelPhi: A Comprehensive Suite for DelPhi Software and Associated Resources. BMC Biophys. 2012, 5, 9 10.1186/2046-1682-5-9. 22583952 PMC3463482

[ref97] Li C.; Jia Z.; Chakravorty A.; Pahari S.; Peng Y.; Basu S.; Koirala M.; Panday S. K.; Petukh M.; Li L.; Alexov E. DelPhi Suite: New Developments and Review of Functionalities. J. Comput. Chem. 2019, 40, 2502–2508. 10.1002/jcc.26006. 31237360 PMC6771749

