# Supplementary material for: The N-terminal Helix-Turn-Helix Motif of Transcription Factors MarA and Rob Drives DNA Recognition
Source: J Phys Chem B. 2021 Jun 17;125(25):6791–806. doi: 10.1021/acs.jpcb.1c00771 (PMC8279559; doi:10.1021/acs.jpcb.1c00771)
Supplement: Supplementary file 1 — jp1c00771_si_001.pdf [file jp1c00771_si_001.pdf]

Supporting Information

The N-Terminal HTH-Motif of Transcription Factors MarA and Rob Drives DNA Recognition

Marina Corbella[a,†], Qinghua Liao[a,†], Cátia Moreira[a], Antonietta Parracino[a], Peter M. Kasson[b,c] and Shina Caroline Lynn Kamerlin[a,*]

[a] Science for Life Laboratory, Department of Chemistry – BMC, Uppsala University, Uppsala, S-751 23, Sweden

[b] Science for Life Laboratory, Department of Cell and Molecular Biology, Uppsala University, Uppsala, S-65124, Sweden

[c] Departments of Molecular Physiology and Biomedical Engineering, University of Virginia, Virginia, VA 22908, USA

[†] These authors contributed equally.

* To whom correspondence should be addressed. Tel: +4618-471 4423; Email: lynn.kamerlin@kemi.uu.se

Table of Contents

Supplementary Figures…………………………………………………………………….…………..S2

Supplementary Tables……………..……………………………………..….…………..............S54

Supplementary References…………...……………..…………………………….……….…………S79

Supplementary Figures

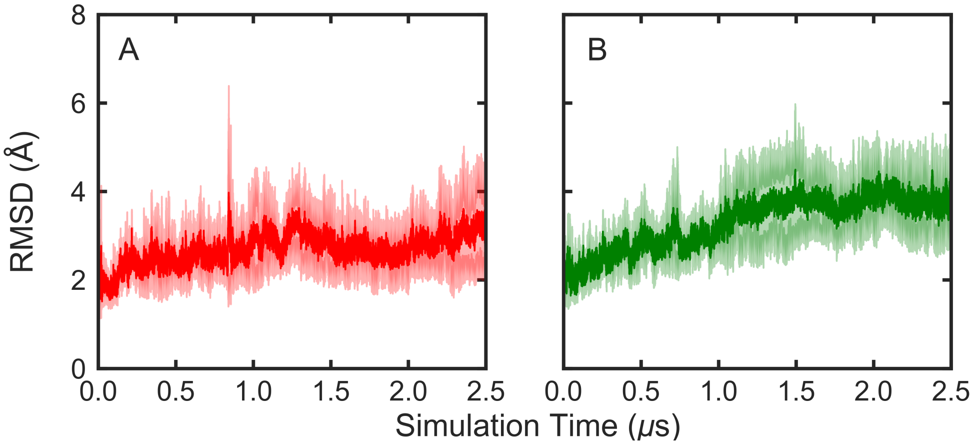

Figure S1. The root mean square deviation (RMSD, Å) of all backbone atoms (N, C_α, C) of (**A**) MarA, and (**B**) Rob in the absence of DNA, calculated over 5 independent 2.5 μs molecular dynamics simulations of free MarA and Rob, and using the corresponding crystal structures of each protein as reference structures. The solid lines denote the average RMSD over all replicas, and the shaded lines show the standard deviations over the different individual replicas.

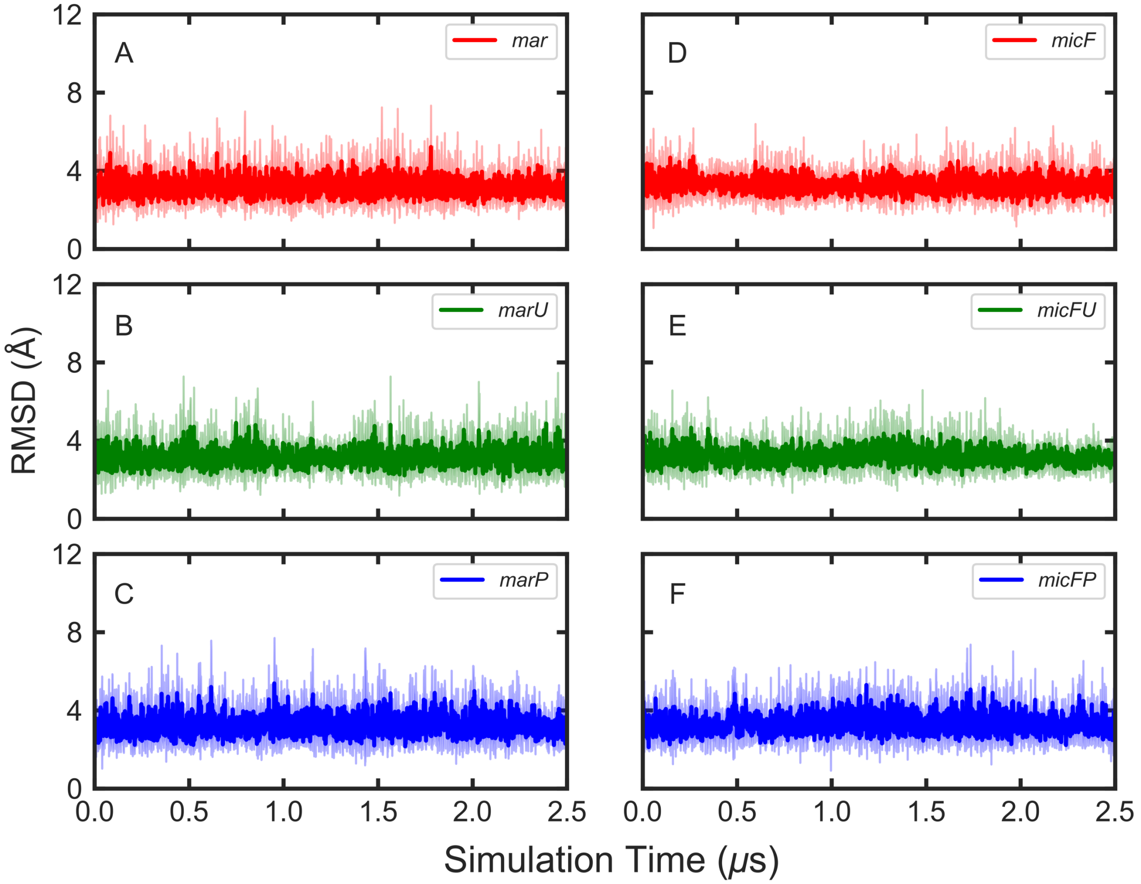

Figure S2. Root mean square deviation (RMSD, Å) of all backbone atoms of the (**A**) *mar,* (**B**) *marU*, (**C**) *marP,* (**D**) *micF*, (**E**) *micFU* and (**F**) *micFP* sequences, calculated over 3 independent 2.5 μs molecular dynamics simulations in the absence of protein (free DNA). The solid lines denote the average RMSD over all replicas, and the shaded lines show the standard deviations over the different individual replicas.

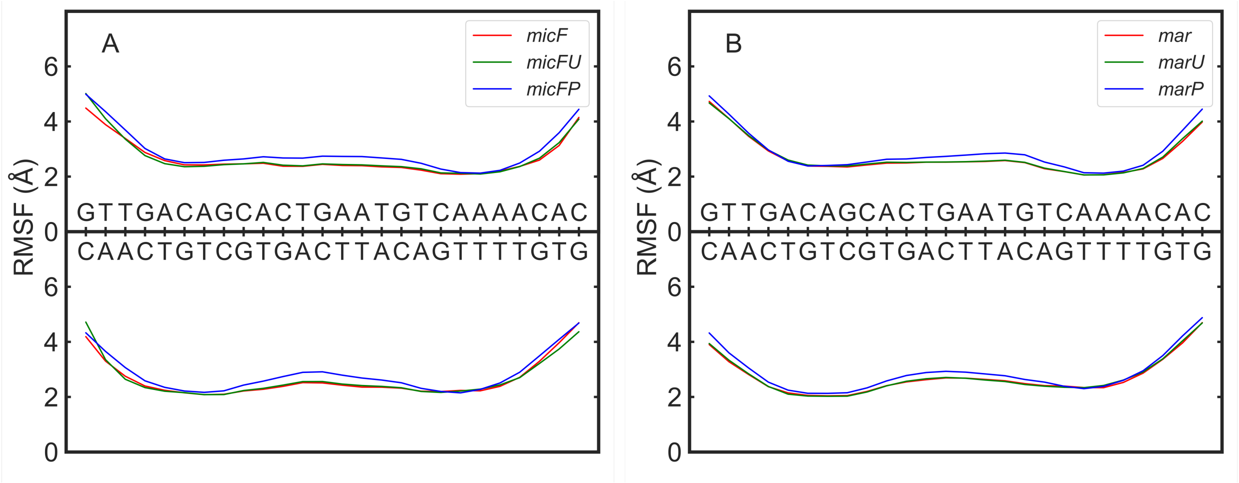

Figure S3. Root mean square fluctuation (RMSF, Å) of the backbone atoms of the (**A**) *micF* (red), *micFU* (green) and *micFP* (blue), and (**B**) *mar* (red), *marU* (green) and *marP* (blue) promoters, calculated over three independent 2.5 μs MD simulations in the absence of protein (free DNA, **Table S3**). Note that the two DNA strands are shown separately (as top/bottom half-panels for each system) for clarity.

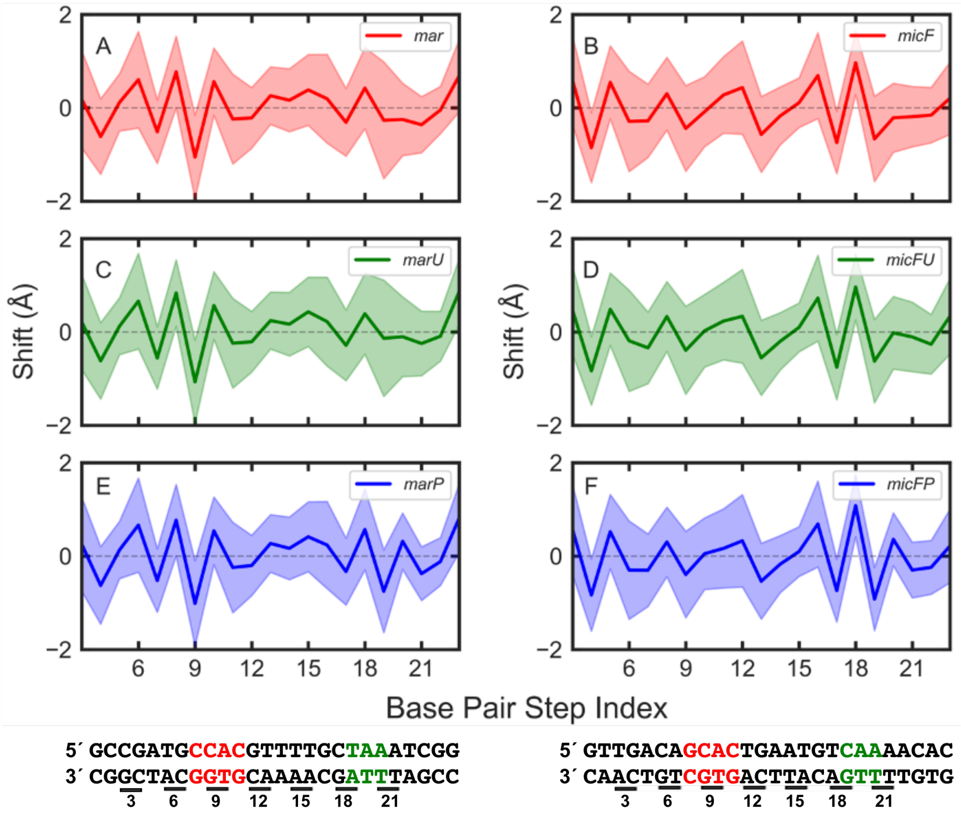

Figure S4. Shift displacement (Å) between two base pairs in one base pair step reference frame, in simulations of the (**A**) *mar*, (**B**) *marU*, (**C**) *marP*, (**D**) *micF*, (**E**) *micFU* and (**F**) *micFP* promoters, in the absence of protein. The solid and shaded lines denote average values and standard deviations per base pair step, averaged over all replicas for each system. Dashed horizontal lines refer to standard B-DNA parameters. The terminal two base pair steps on each end of the DNA strand were not analyzed to avoid end fraying effects.

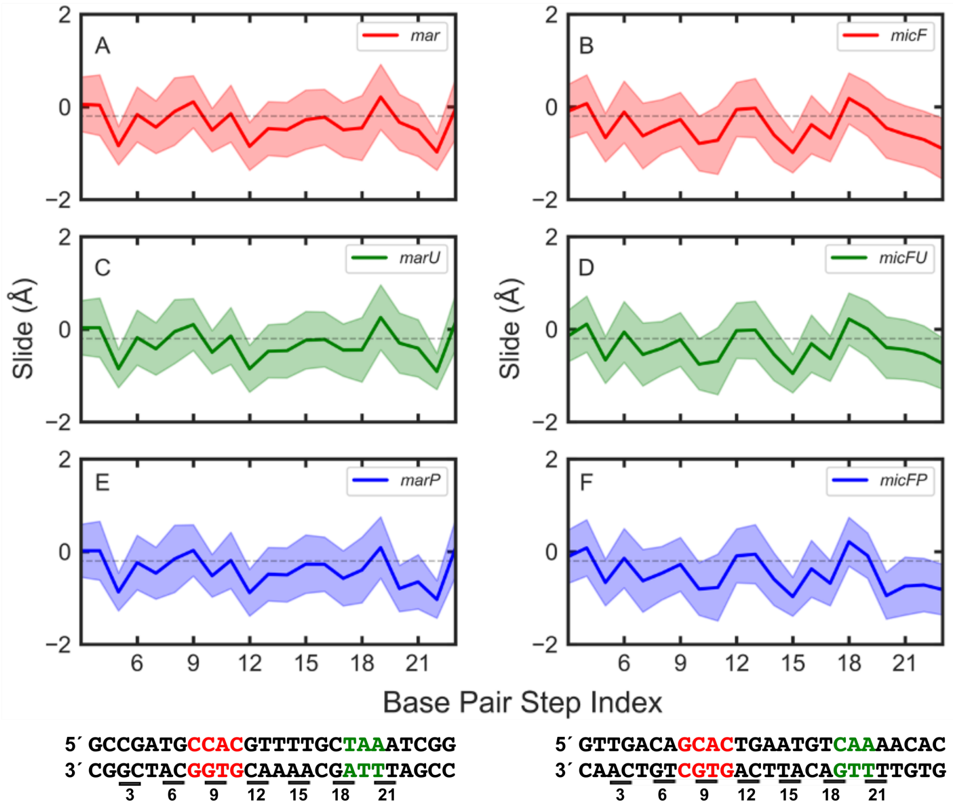

Figure S5. Slide displacement (Å) between two base pairs in one base pair step reference frame, in simulations of the (**A**) *mar*, (**B**) *marU*, (**C**) *marP*, (**D**) *micF*, (**E**) *micFU* and (**F**) *micFP* promoters, in the absence of protein. The solid and shaded lines denote average values and standard deviations per base pair step, averaged over all replicas for each system. Dashed horizontal lines refer to standard B-DNA parameters. The terminal two base pair steps on each end of the DNA strand were not analyzed to avoid end fraying effects.

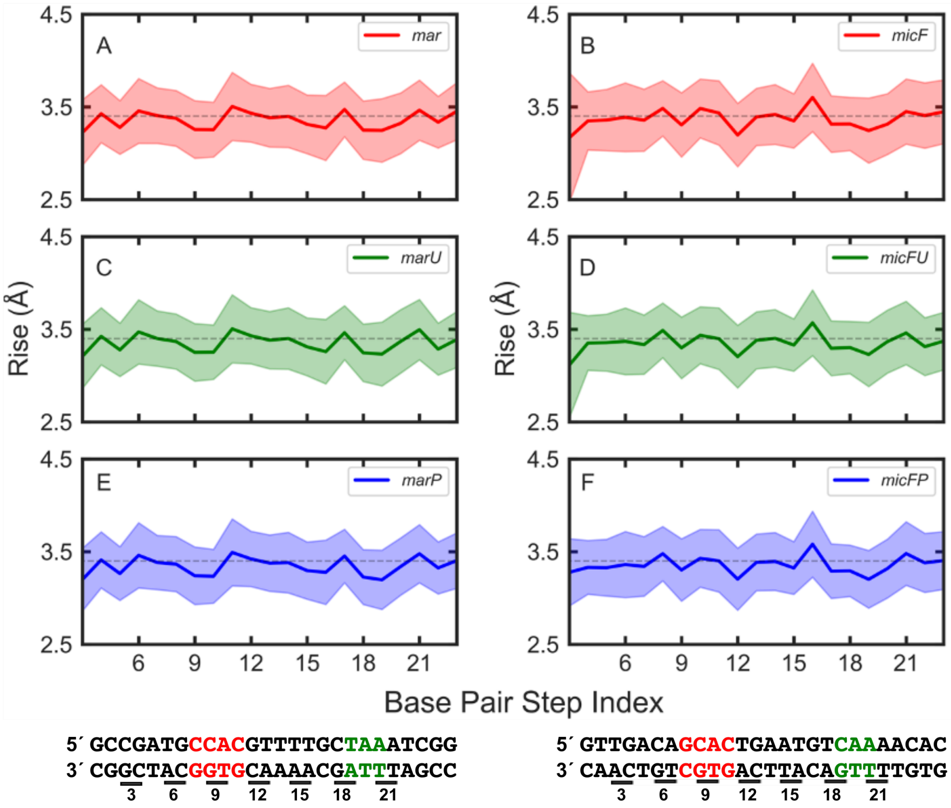

Figure S6. Rise displacement (Å) between two base pairs in one base pair step reference frame, in simulations of the (**A**) *mar*, (**B**) *marU*, (**C**) *marP*, (**D**) *micF*, (**E**) *micFU* and (**F**) *micFP* promoters, in the absence of protein. The solid and shaded lines denote average values and standard deviations per base pair step, averaged over all replicas for each system. Dashed horizontal lines refer to standard B-DNA parameters. The terminal two base pair steps on each end of the DNA strand were not analyzed to avoid end fraying effects.

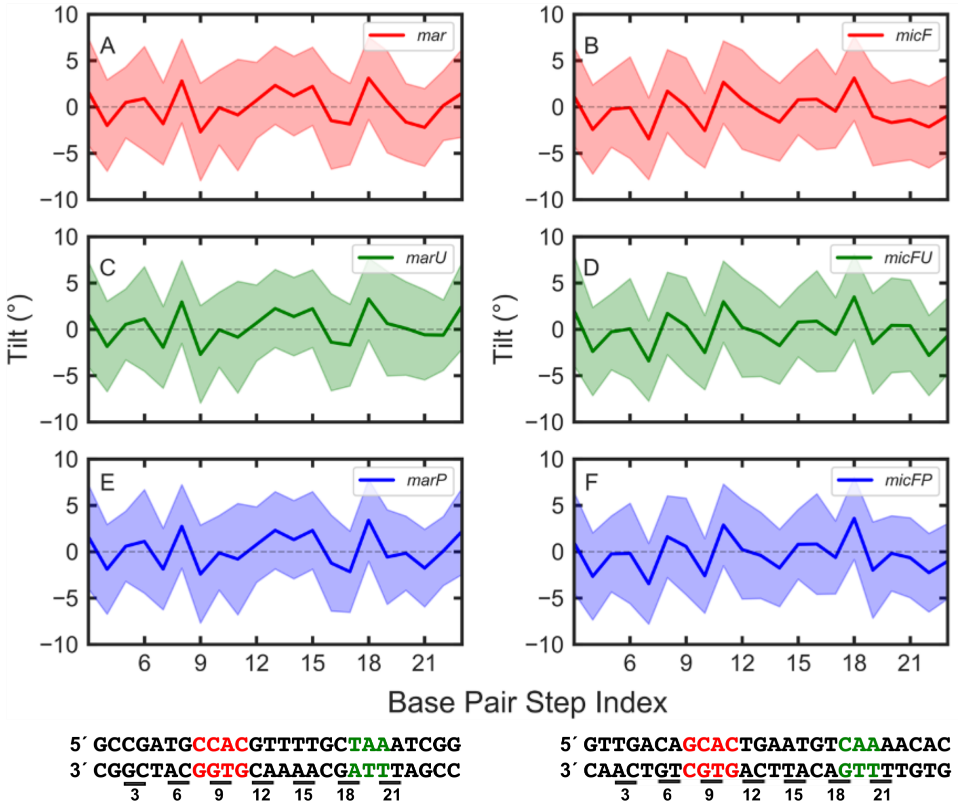

Figure S7. Tilt angle (°) between two base pairs in one base pair step reference frame, in simulations of the (**A**) *mar*, (**B**) *marU*, (**C**) *marP*, (**D**) *micF*, (**E**) *micFU* and (**F**) *micFP* promoters, in the absence of protein. The solid and shaded lines denote average values and standard deviations per base pair step, averaged over all replicas for each system. Dashed horizontal lines refer to standard B-DNA parameters. The terminal two base pair steps on each end of the DNA strand were not analyzed to avoid end fraying effects.

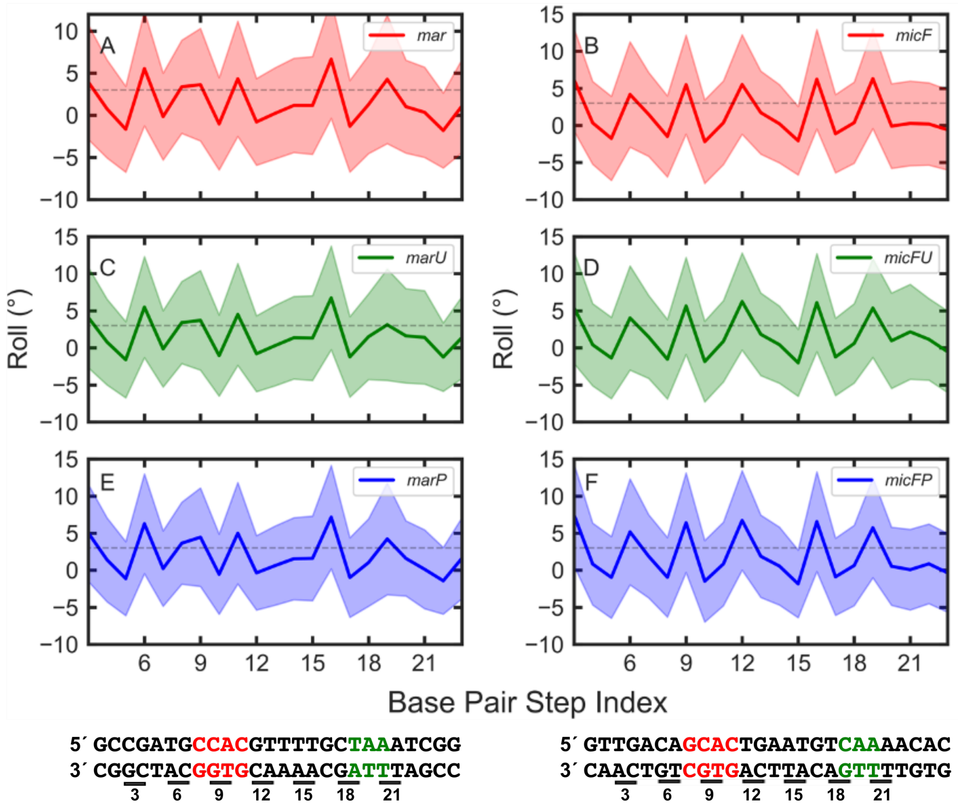

Figure S8. Roll angle (°) between two base pairs in one base pair step reference frame, in simulations of the (**A**) *mar*, (**B**) *marU*, (**C**) *marP*, (**D**) *micF*, (**E**) *micFU* and (**F**) *micFP* promoters, in the absence of protein. The solid and shaded lines denote average values and standard deviations per base pair step, averaged over all replicas for each system. Dashed horizontal lines refer to standard B-DNA parameters. The terminal two base pair steps on each end of the DNA strand were not analyzed to avoid end fraying effects.

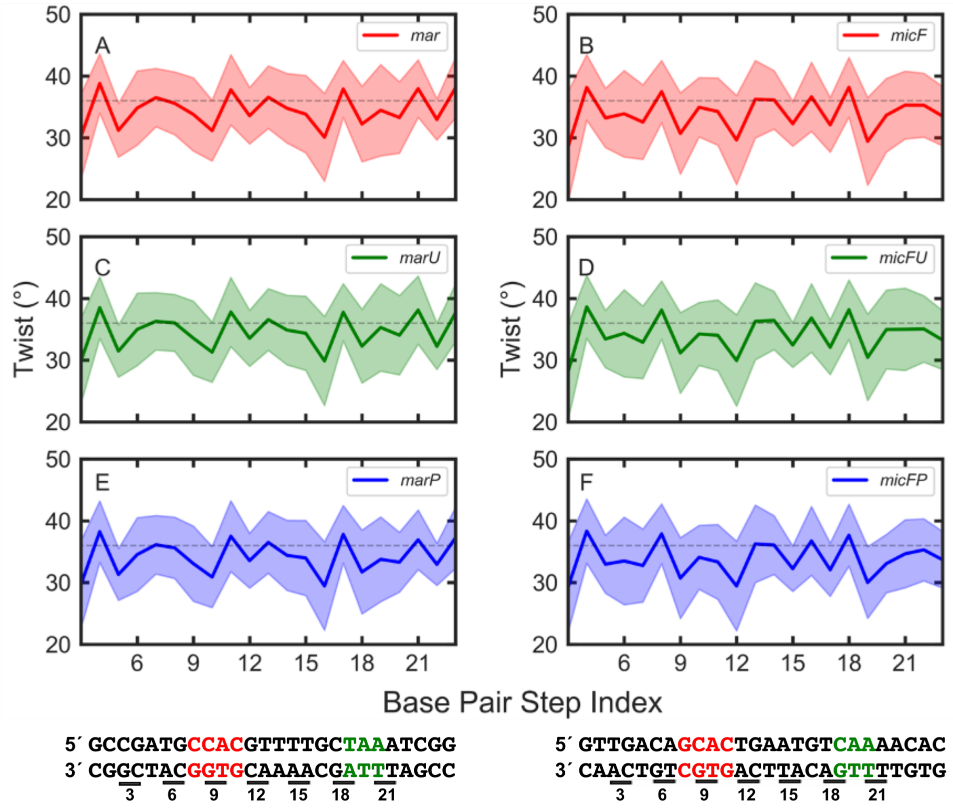

Figure S9. Twist angle (°) between two base pairs in one base pair step reference frame, in simulations of the (**A**) *mar*, (**B**) *marU*, (**C**) *marP*, (**D**) *micF*, (**E**) *micFU* and (**F**) *micFP* promoters, in the absence of protein. The solid and shaded lines denote average values and standard deviations per base pair step, averaged over all replicas for each system. Dashed horizontal lines refer to standard B-DNA parameters. The terminal two base pair steps on each end of the DNA strand were not analyzed to avoid end fraying effects.

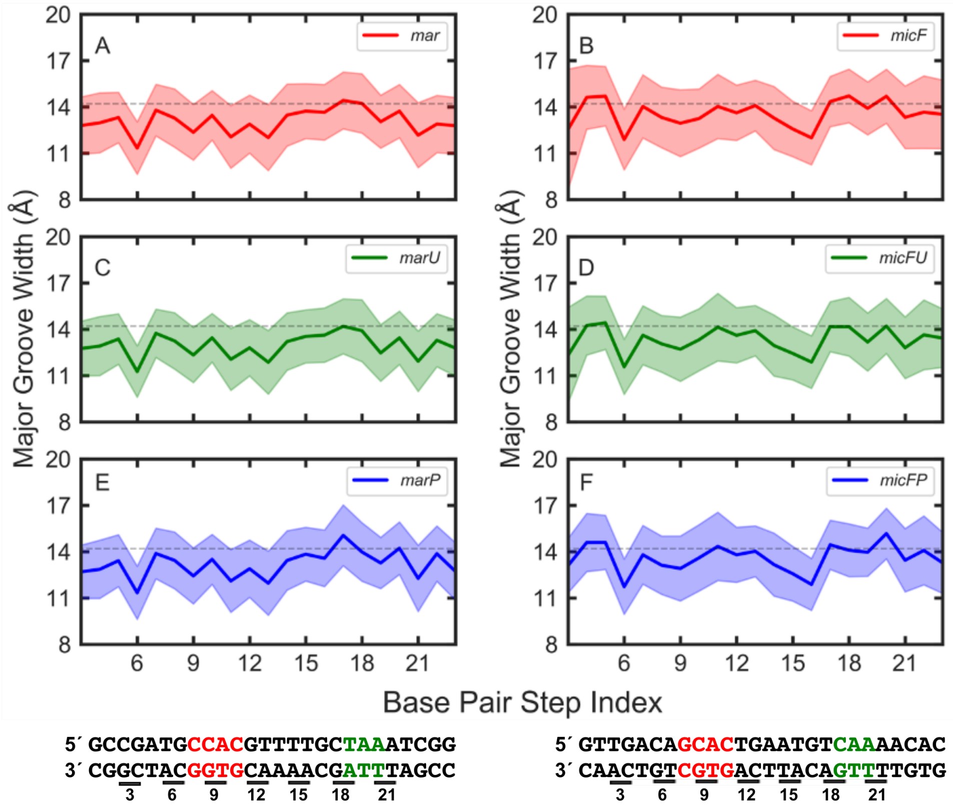

Figure S10. Major groove width (Å) between two base pairs in one base pair step reference frame, in simulations of the (**A**) *mar*, (**B**) *marU*, (**C**) *marP*, (**D**) *micF*, (**E**) *micFU* and (**F**) *micFP* promoters, in the absence of protein. The solid and shaded lines denote average values and standard deviations per base pair step, averaged over all replicas for each system. Dashed horizontal lines refer to standard B-DNA parameters. The terminal two base pair steps on each end of the DNA strand were not analyzed to avoid end fraying effects.

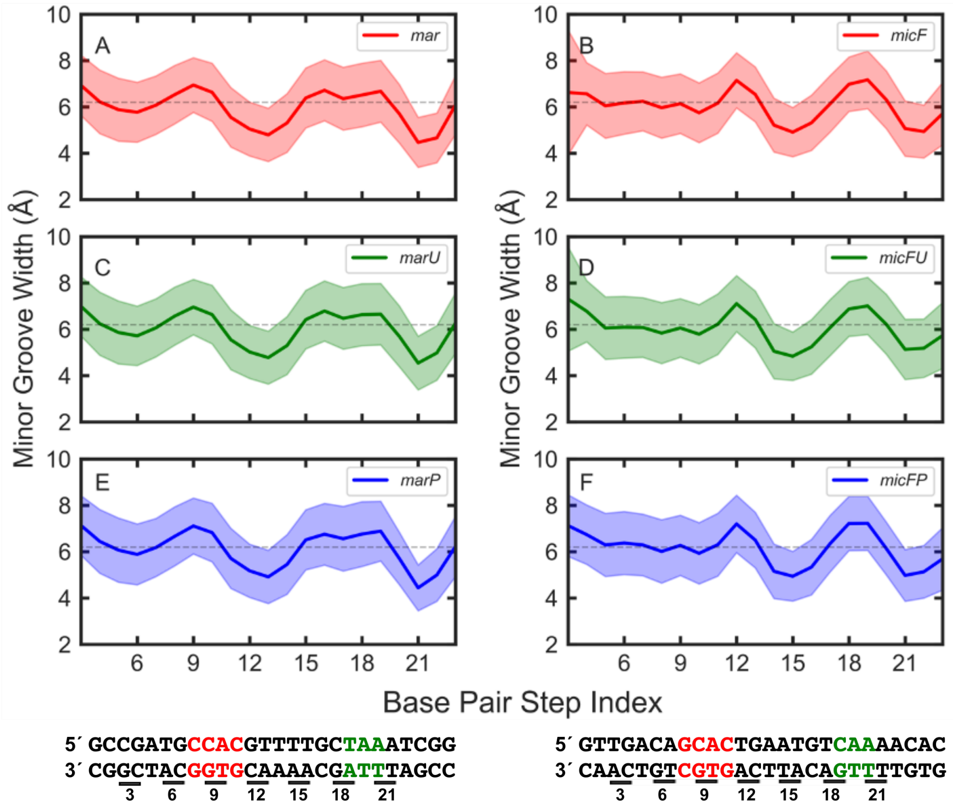

Figure S11. Minor groove width (Å) between two base pairs in one base pair step reference frame, in simulations of the (**A**) *mar*, (**B**) *marU*, (**C**) *marP*, (**D**) *micF*, (**E**) *micFU* and (**F**) *micFP* promoters, in the absence of protein. The solid and shaded lines denote average values and standard deviations per base pair step, averaged over all replicas for each system. Dashed horizontal lines refer to standard B-DNA parameters. The terminal two base pair steps on each end of the DNA strand were not analyzed to avoid end fraying effects.

A

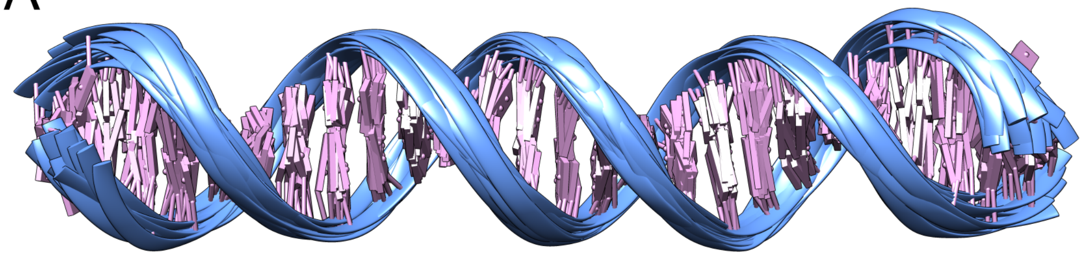

B

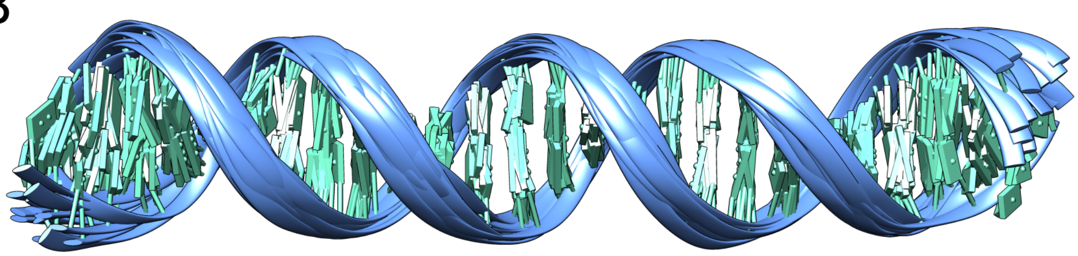

Figure S12. Overlay of the most-populated structures of each 500 ns time period along 3 x 2.5 μs molecular dynamics simulations of the free (**A**) *mar* and (**B**) *micF* promoters, in the absence of protein. For the sequences of these promoters, see **Table S1**. The most-populated structures were obtained by clustering each 500 ns interval of each individual trajectory using a hierarchical algorithm, as implemented in CPPTRAJ[1]. The clustering was performed based on pairwise RMSD calculations over all atoms, but omitting the terminal base pairs from the clustering to avoid fraying artefacts.

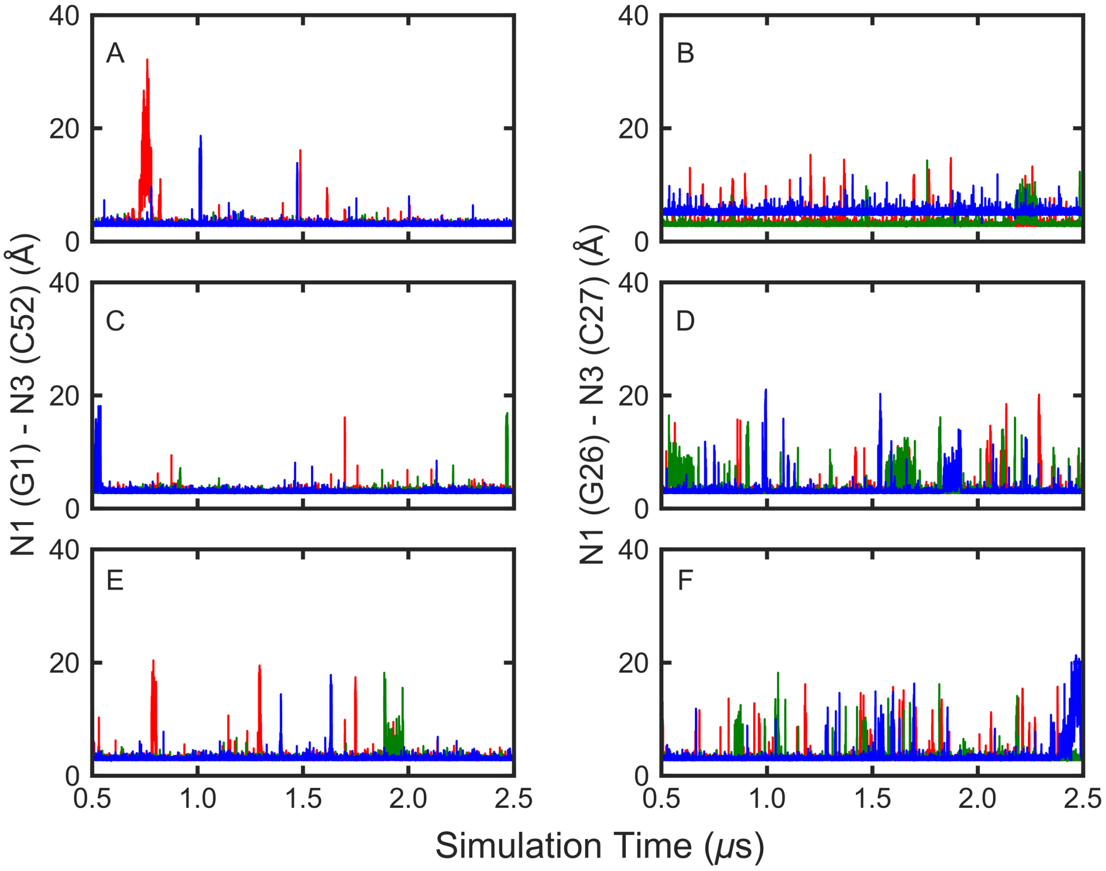

Figure S13. Monitoring the fraying of the (**A**, **C**, **E**) G1-C52 and (**B**, **D**, **F**) G26-C27 terminal base pairs during the last 2 μs of 3 × 2.5 μs molecular dynamics simulations of the (**A**, **B**) *mar*, (**C**, **D**) *marU* and (**E**, **F**) *marP* promoters in the absence of protein. Fraying is calculated based on the distance between N1 of guanine and N3 of cytosine of the last base pairs.

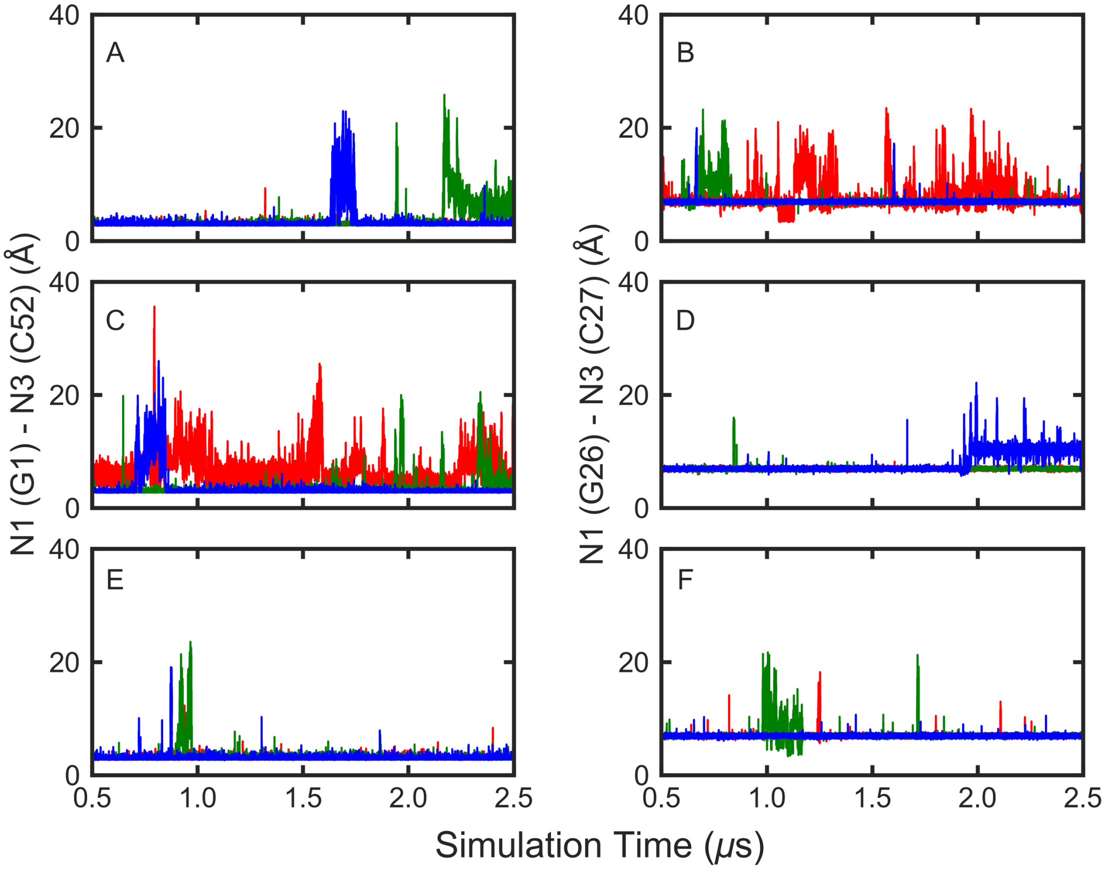

Figure S14. Monitoring the fraying of the (**A**, **C**, **E**) G1-C52 and (**B**, **D**, **F**) G26-C27 terminal base pairs during the last 2 μs of 3 × 2.5 μs molecular dynamics simulations of (**A**, **B**) *micF*, (**C**, **D**) *micU* and (**E**, **F**) *micFP* in the absence of protein. Fraying is calculated based on the distance between N1 of guanine and N3 of cytosine of the last base pairs.

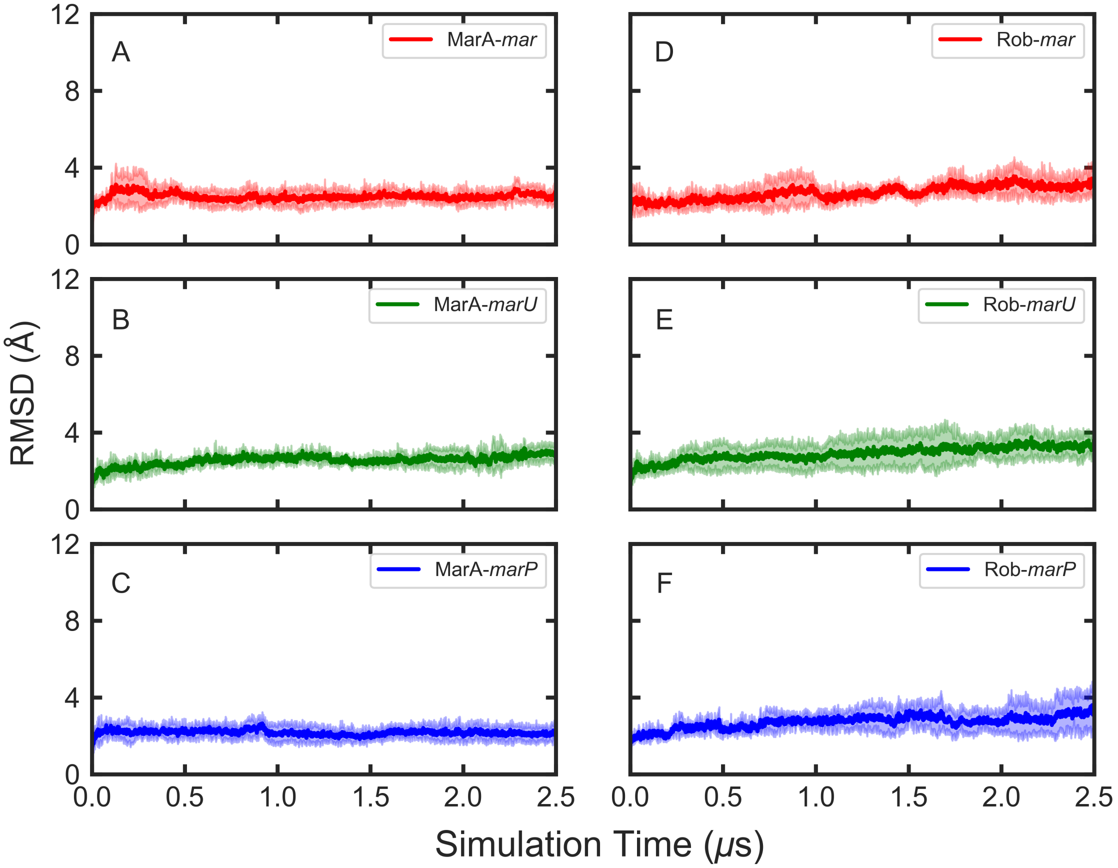

Figure S15. Root mean square deviation (RMSD, Å) of all protein backbone atoms of MarA in complex with the (**A**) *mar,* (**B**) *marU*, (**C**) *marP* promoters*,* and Rob in complex with the (**D**) *mar*, (**E**) *marU* and (**F**) *marP* promoters, calculated over 5 independent 2.5 μs molecular dynamics simulations, and relative to the initial constructs. The solid lines denote the average RMSD over all replicas, and the shaded lines show the standard deviations over the different individual replicas.

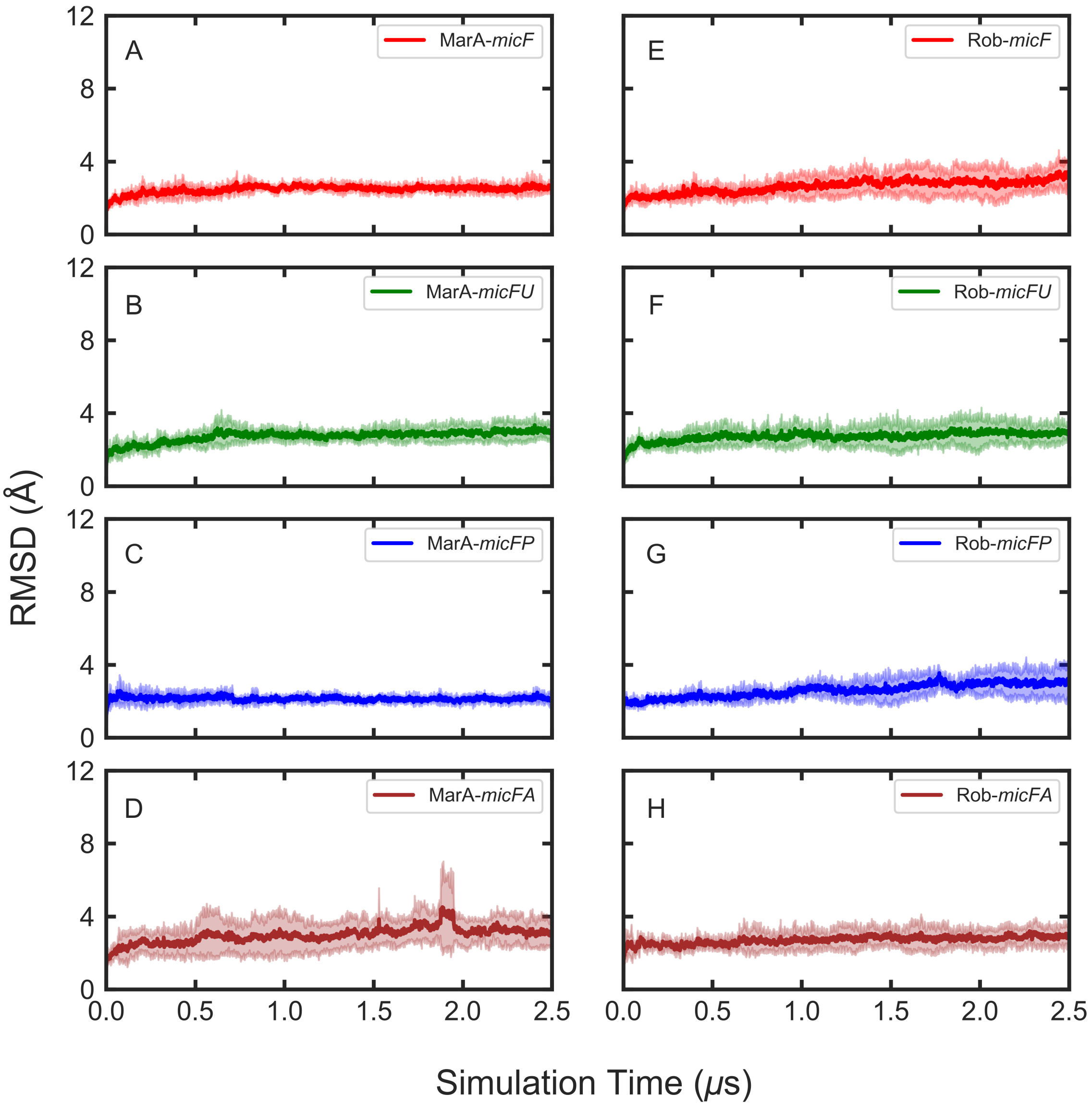

Figure S16. Root mean square deviation (RMSD, Å) of all protein backbone atoms of MarA in complex with the (**A**) *micF,* (**B**) *micFU*, (**C**) *micFP,* (**D**) *micFA* promoters*,* and Rob in complex with the (**E**) *micF*, (**F**) *micFU*, (**G**) *micFP* and (**H**) *micFA* promoters, calculated over 5 independent 2.5 μs molecular dynamics simulations, and relative to the initial constructs. The solid lines denote the average RMSD over all replicas, and the shaded lines show the standard deviations over the different individual replicas.

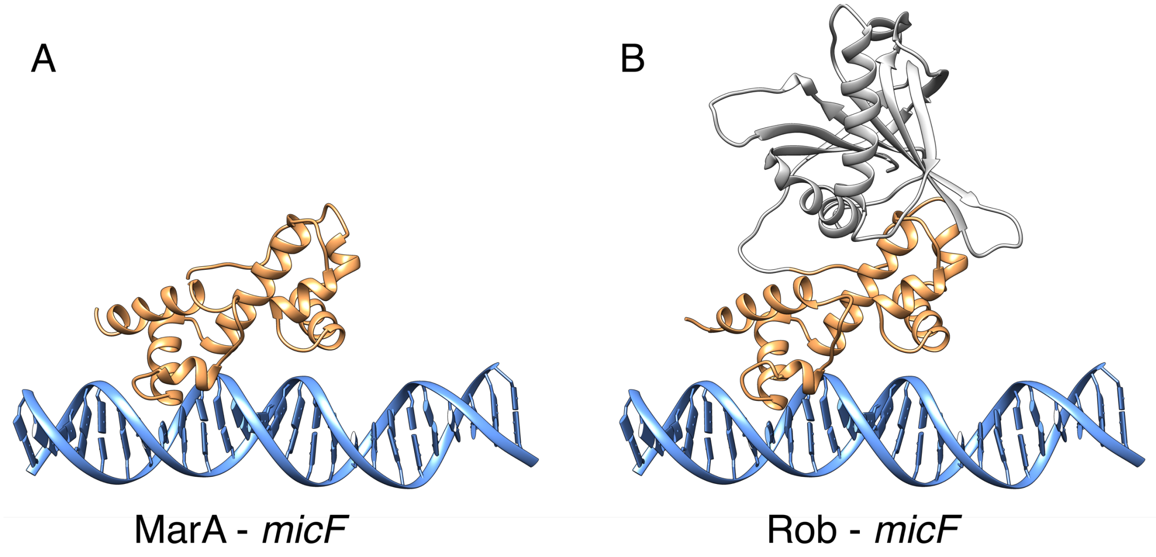

Figure S17. Starting structures of our MD simulations of (**A**) MarA in complex with the *micF* promoter and (**B**) Rob in complex with the *micF* promoter. Note that the MarA structure is an artificial construct on straight rather than bent DNA, generated as described in the main text.

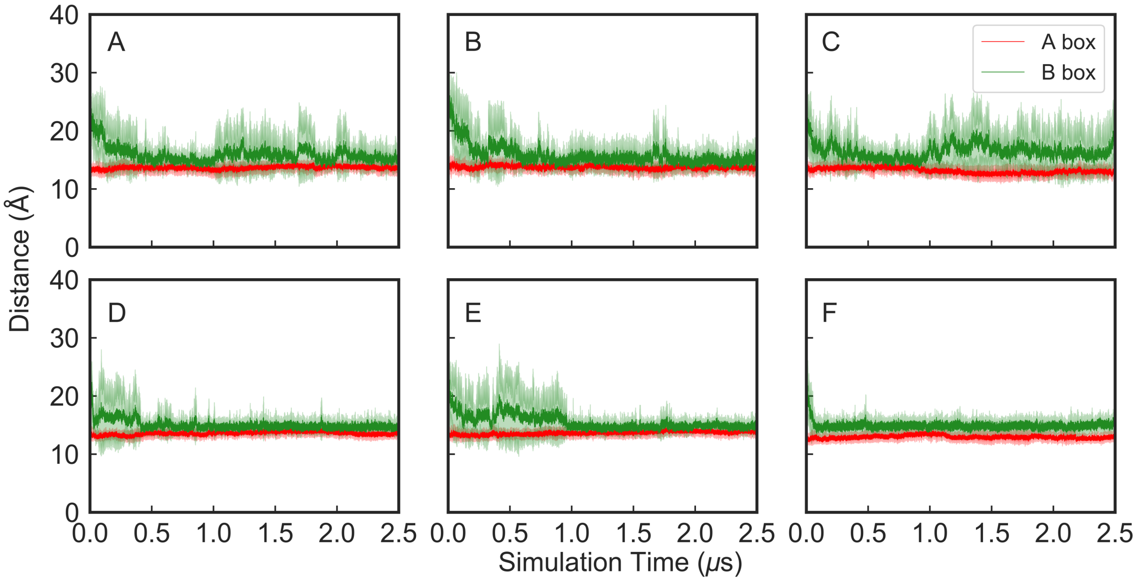

Figure S18. Average distances between the helices inserted inside the major groove and the base pairs at A- and B-boxes, during 5 x 2.5 μs simulations of MarA complexes with promoters (**A**) *mar*, (**B**) *marU,* (**C**) *marP*, (**D**) *micF*, (**E**) *micFU* and (**F**) *micFP.* The solid and shaded lines denote the average values and standard deviations over different replicas, respectively. The distance analysis was performed using PLUMED v. 2.5[2], based on snapshots extracted every 10 ps of the simulations.

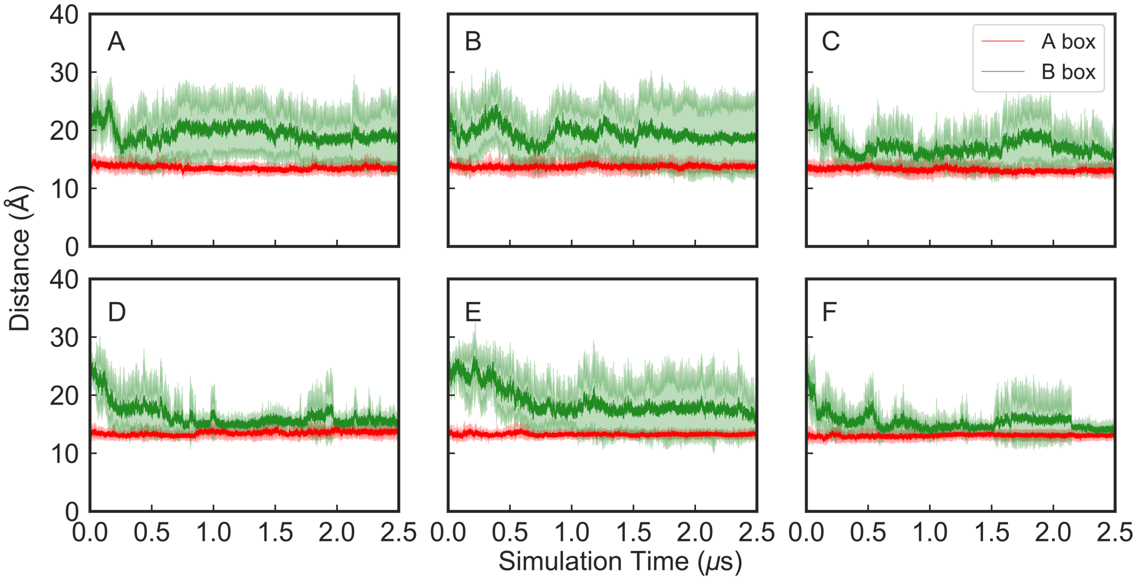

Figure S19. Average distances between the helices inserted inside the major groove and the base pairs at A- and B-boxes, during 5 x 2.5 μs simulations of Rob complexes with promoters (**A**) *mar*, (**B**) *marU,* (**C**) *marP*, (**D**) *micF*, (**E**) *micFU* and (**F**) *micFP.* The solid and shaded lines denote the average values and standard deviations over different replicas, respectively. The distance analysis was performed using PLUMED v. 2.5[2], based on snapshots extracted every 10 ps of the simulations.

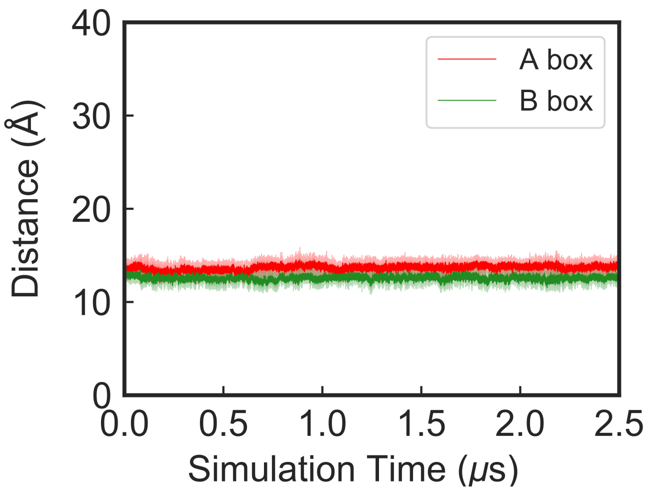

Figure S20. Average distances between the helices inserted inside the major groove and the base pairs at A- and B-boxes, during 5 x 2.5 μs simulations of the crysallographic MarA-*mar* conformation. The solid and shaded lines denote the average values and standard deviations over different replicas, respectively. The distance analysis was performed using PLUMED v. 2.5[2], based on snapshots extracted every 10 ps of the simulations.

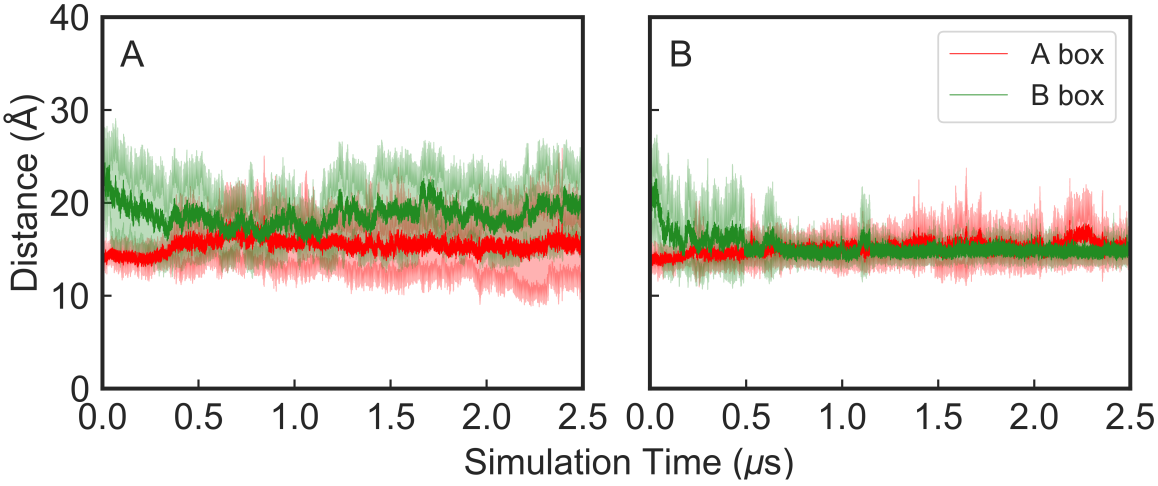

Figure S21. Average distances between the helices inserted inside the major groove and the base pairs at the A- and B-boxes during 5 x 2.5 μs simulations of (**A**) Rob and (**B**) MarA in complex with the mutant promoter sequence *micFA.* The solid and shaded lines denote the average values and standard deviations over different replicas, respectively. The distance analysis was performed using PLUMED v2.5[2] based on snapshots extracted every 10 ps of the simulations.

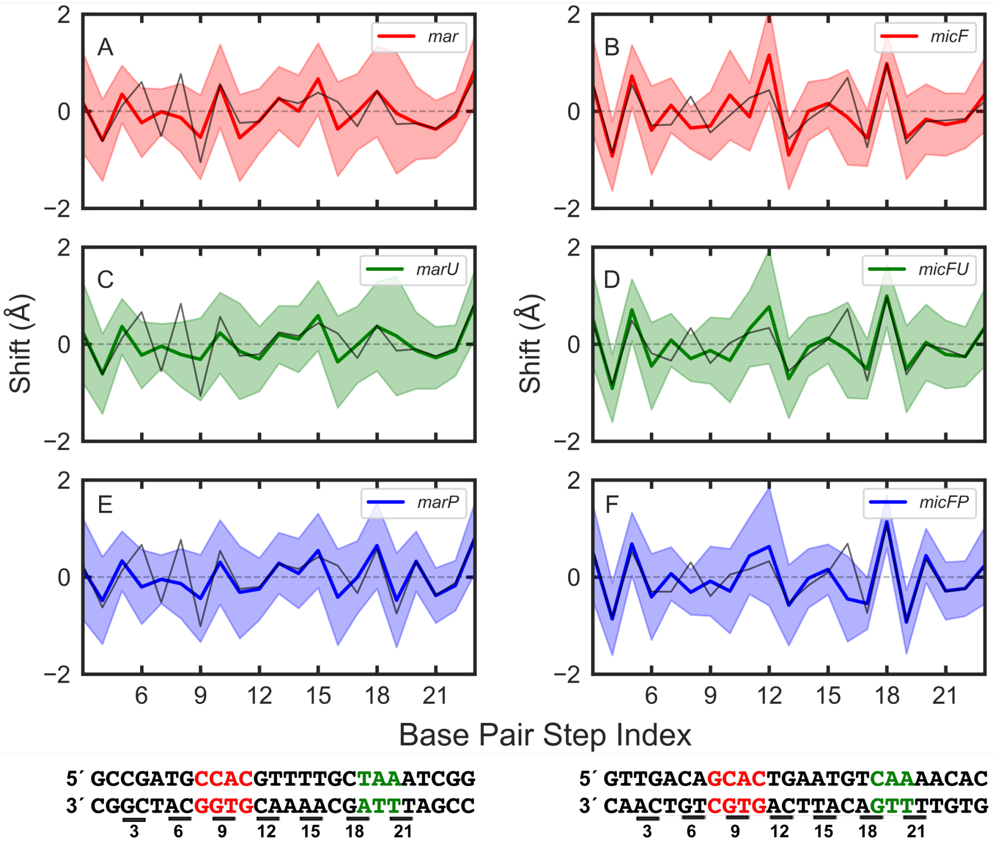

Figure S22. Shift displacement (Å) between two base pairs in one base pair step reference frame, in simulations of the (**A**) *mar*, (**B**) *marU*, (**C**) *marP*, (**D**) *micF*, (**E**) *micFU* and (**F**) *micFP* promoters, in complex with MarA. The solid and shaded lines denote average values and standard deviations per base pair step, averaged over all replicas for each system. Black solid lines denote average values for the corresponding free DNA sequences. Dashed horizontal lines refer to standard B-DNA parameters. The terminal two base pair steps on each end of the DNA strand were not analyzed to avoid end fraying effects.

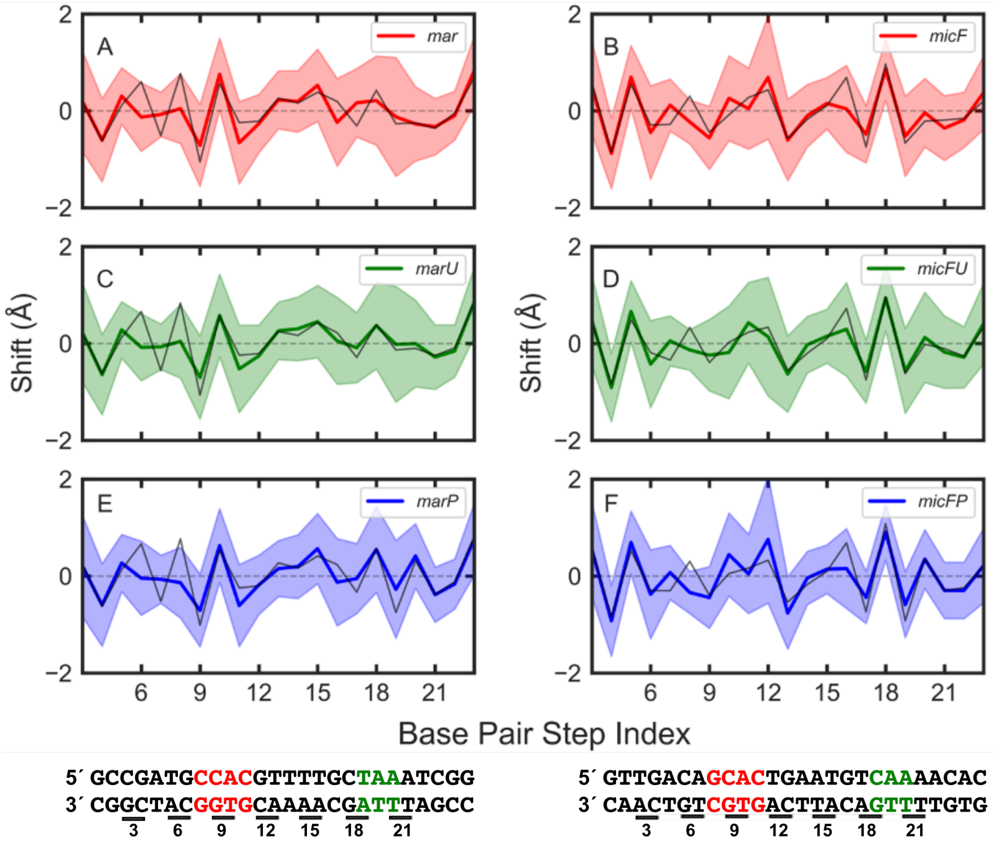

Figure S23. Shift displacement (Å) between two base pairs in one base pair step reference frame, in simulations of the (**A**) *mar*, (**B**) *marU*, (**C**) *marP*, (**D**) *micF*, (**E**) *micFU* and (**F**) *micFP* promoters, in complex with Rob. The solid and shaded lines denote average values and standard deviations per base pair step, averaged over all replicas for each system. Black solid lines denote average values for the corresponding free DNA sequences. Dashed horizontal lines refer to standard B-DNA parameters. The terminal two base pair steps on each end of the DNA strand were not analyzed to avoid end fraying effects.

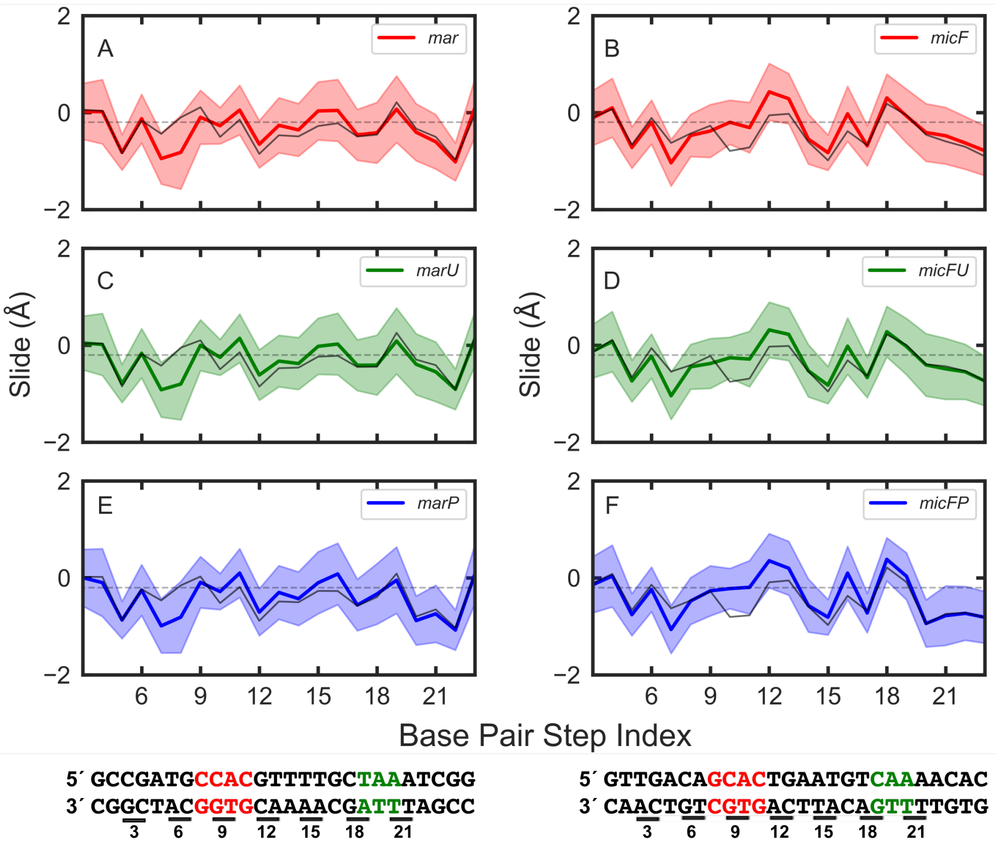

Figure S24. Slide displacement (Å) between two base pairs in one base pair step reference frame, in simulations of the (**A**) *mar*, (**B**) *marU*, (**C**) *marP*, (**D**) *micF*, (**E**) *micFU* and (**F**) *micFP* promoters, in complex with MarA. The solid and shaded lines denote average values and standard deviations per base pair step, averaged over all replicas for each system. Black solid lines denote average values for the corresponding free DNA sequences. Dashed horizontal lines refer to standard B-DNA parameters. The terminal two base pair steps on each end of the DNA strand were not analyzed to avoid end fraying effects.

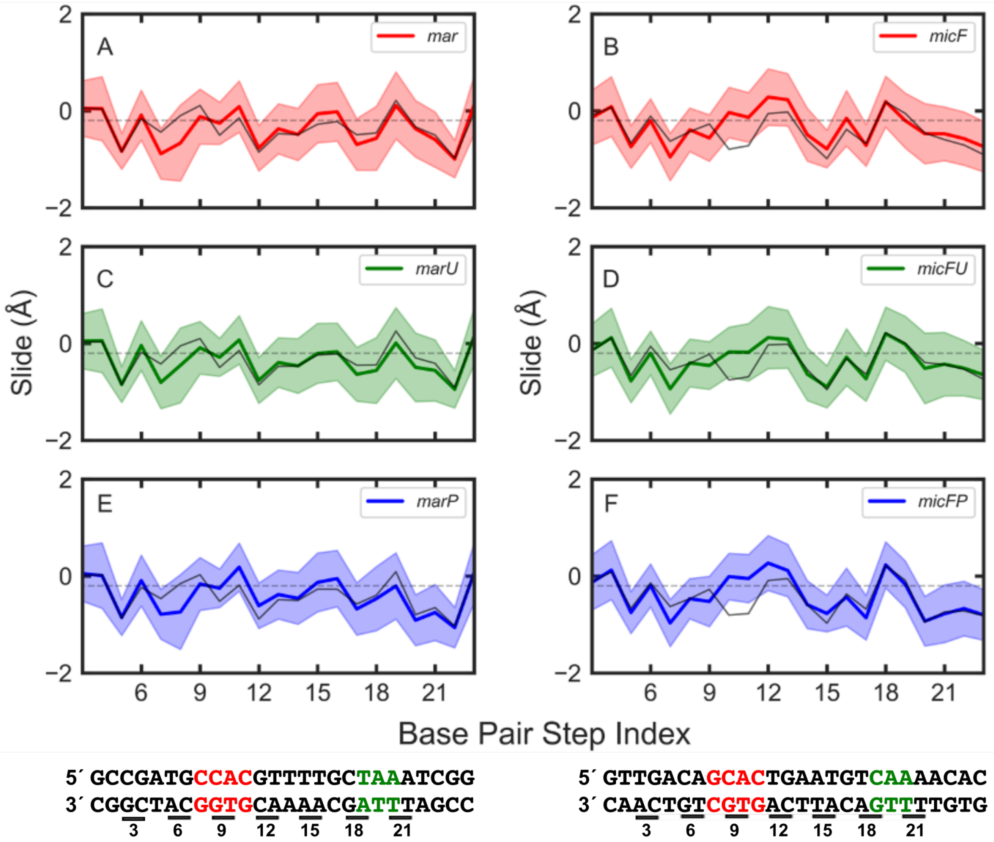

Figure S25. Slide displacement (Å) between two base pairs in one base pair step reference frame, in simulations of the (**A**) *mar*, (**B**) *marU*, (**C**) *marP*, (**D**) *micF*, (**E**) *micFU* and (**F**) *micFP* promoters, in complex with Rob. The solid and shaded lines denote average values and standard deviations per base pair step, averaged over all replicas for each system. Black solid lines denote average values for the corresponding free DNA sequences. Dashed horizontal lines refer to standard B-DNA parameters. The terminal two base pair steps on each end of the DNA strand were not analyzed to avoid end fraying effects.

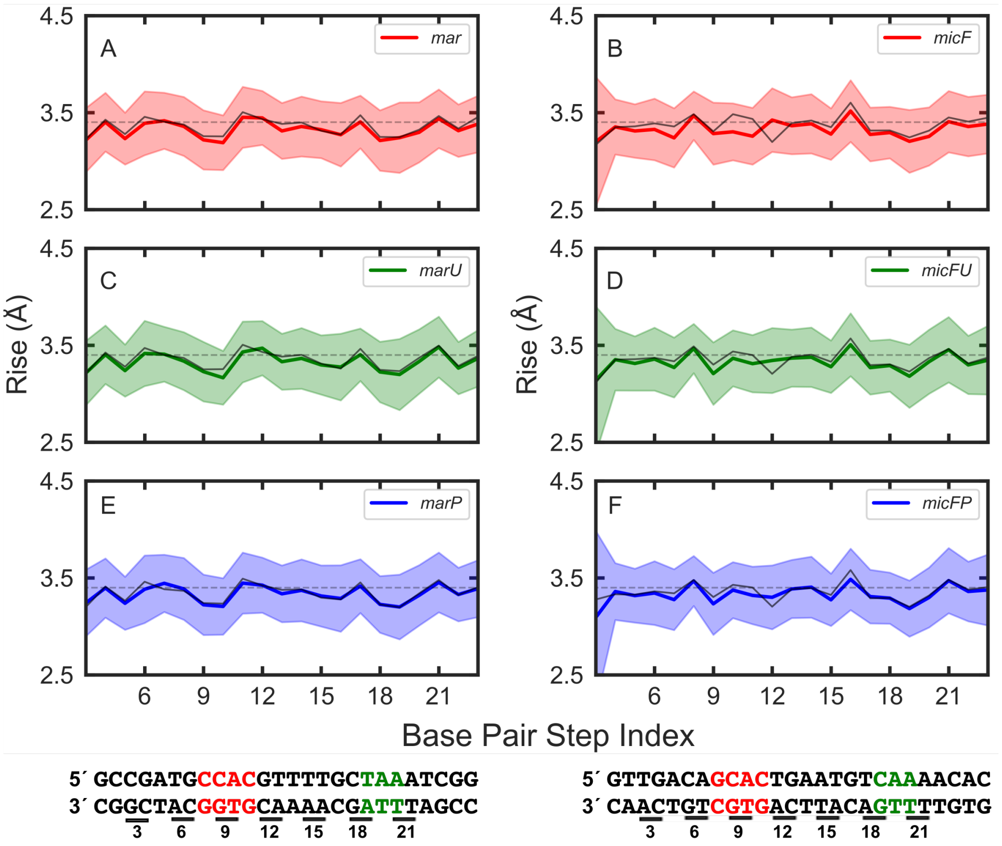

Figure S26. Rise displacement (Å) between two base pairs in one base pair step reference frame, in simulations of the (**A**) *mar*, (**B**) *marU*, (**C**) *marP*, (**D**) *micF*, (**E**) *micFU* and (**F**) *micFP* promoters, in complex with MarA. The solid and shaded lines denote average values and standard deviations per base pair step, averaged over all replicas for each system. Black solid lines denote average values for the corresponding free DNA sequences. Dashed horizontal lines refer to standard B-DNA parameters. The terminal two base pair steps on each end of the DNA strand were not analyzed to avoid end fraying effects.

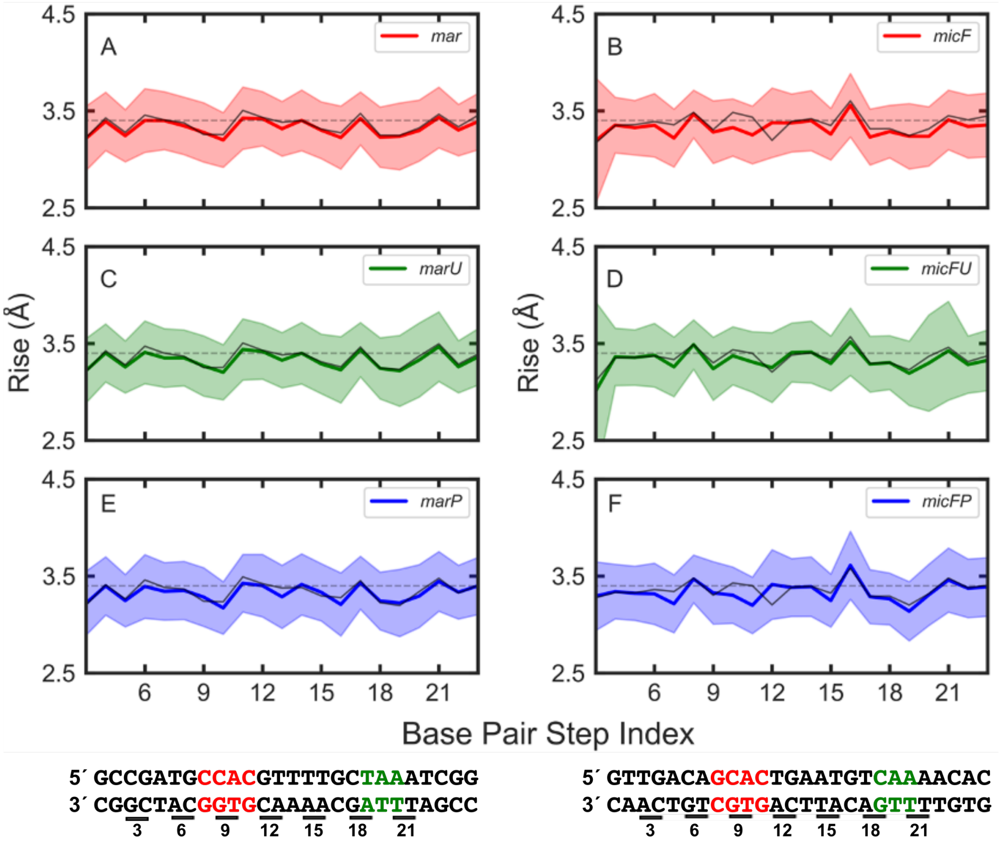

Figure S27. Rise displacement (Å) between two base pairs in one base pair step reference frame, in simulations of the (**A**) *mar*, (**B**) *marU*, (**C**) *marP*, (**D**) *micF*, (**E**) *micFU* and (**F**) *micFP* promoters, in complex with Rob. The solid and shaded lines denote average values and standard deviations per base pair step, averaged over all replicas for each system. Black solid lines denote average values for the corresponding free DNA sequences. Dashed horizontal lines refer to standard B-DNA parameters. The terminal two base pair steps on each end of the DNA strand were not analyzed to avoid end fraying effects.

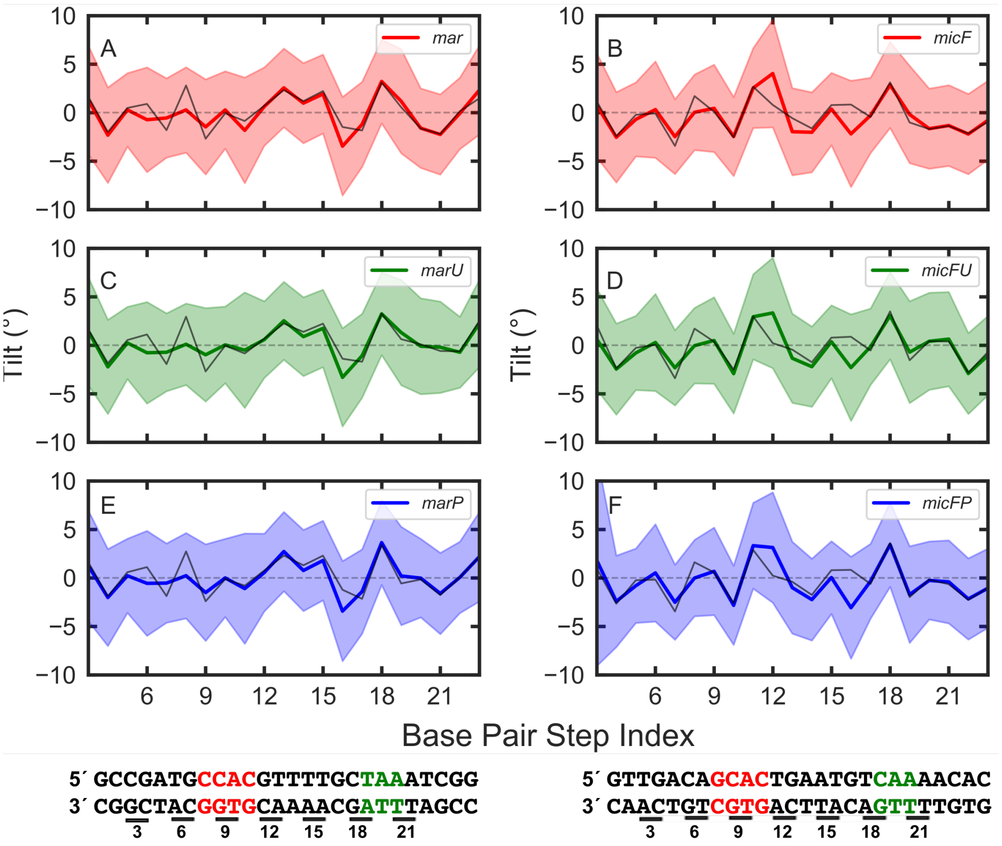

Figure S28. Tilt angle (°) between two base pairs in one base pair step reference frame, in simulations of the (**A**) *mar*, (**B**) *marU*, (**C**) *marP*, (**D**) *micF*, (**E**) *micFU* and (**F**) *micFP* promoters, in complex with MarA. The solid and shaded lines denote average values and standard deviations per base pair step, averaged over all replicas for each system. Black solid lines denote average values for the corresponding free DNA sequences. Dashed horizontal lines refer to standard B-DNA parameters. The terminal two base pair steps on each end of the DNA strand were not analyzed to avoid end fraying effects.

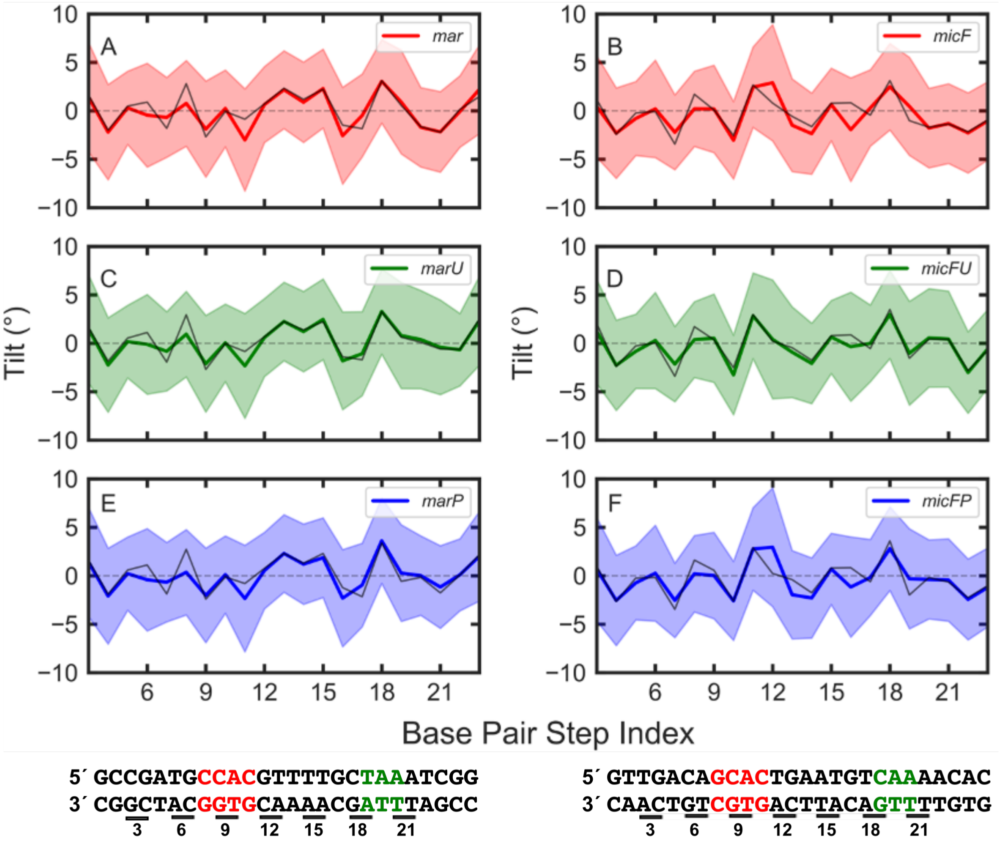

Figure S29. Tilt angle (°) between two base pairs in one base pair step reference frame, in simulations of the (**A**) *mar*, (**B**) *marU*, (**C**) *marP*, (**D**) *micF*, (**E**) *micFU* and (**F**) *micFP* promoters, in complex with Rob. The solid and shaded lines denote average values and standard deviations per base pair step, averaged over all replicas for each system. Black solid lines denote average values for the corresponding free DNA sequences. Dashed horizontal lines refer to standard B-DNA parameters. The terminal two base pair steps on each end of the DNA strand were not analyzed to avoid end fraying effects.

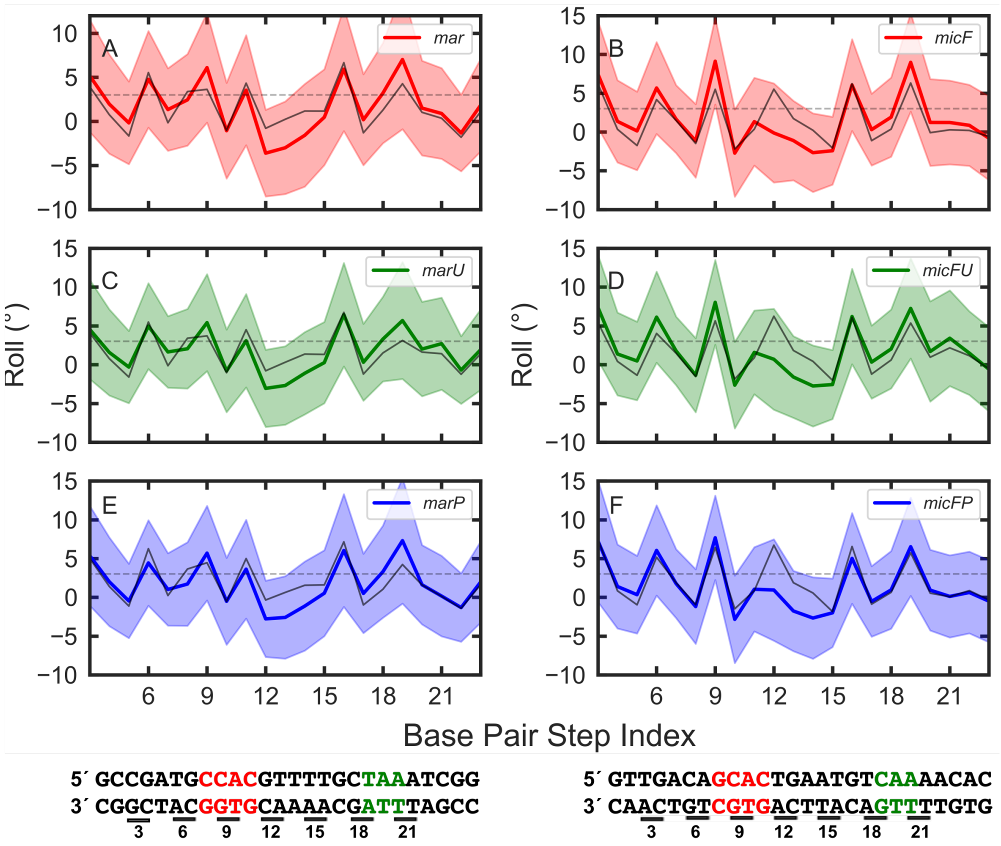

Figure S30. Roll angle (°) between two base pairs in one base pair step reference frame, in simulations of the (**A**) *mar*, (**B**) *marU*, (**C**) *marP*, (**D**) *micF*, (**E**) *micFU* and (**F**) *micFP* promoters, in complex with MarA. The solid and shaded lines denote average values and standard deviations per base pair step, averaged over all replicas for each system. Black solid lines denote average values for the corresponding free DNA sequences. Dashed horizontal lines refer to standard B-DNA parameters. The terminal two base pair steps on each end of the DNA strand were not analyzed to avoid end fraying effects.

.

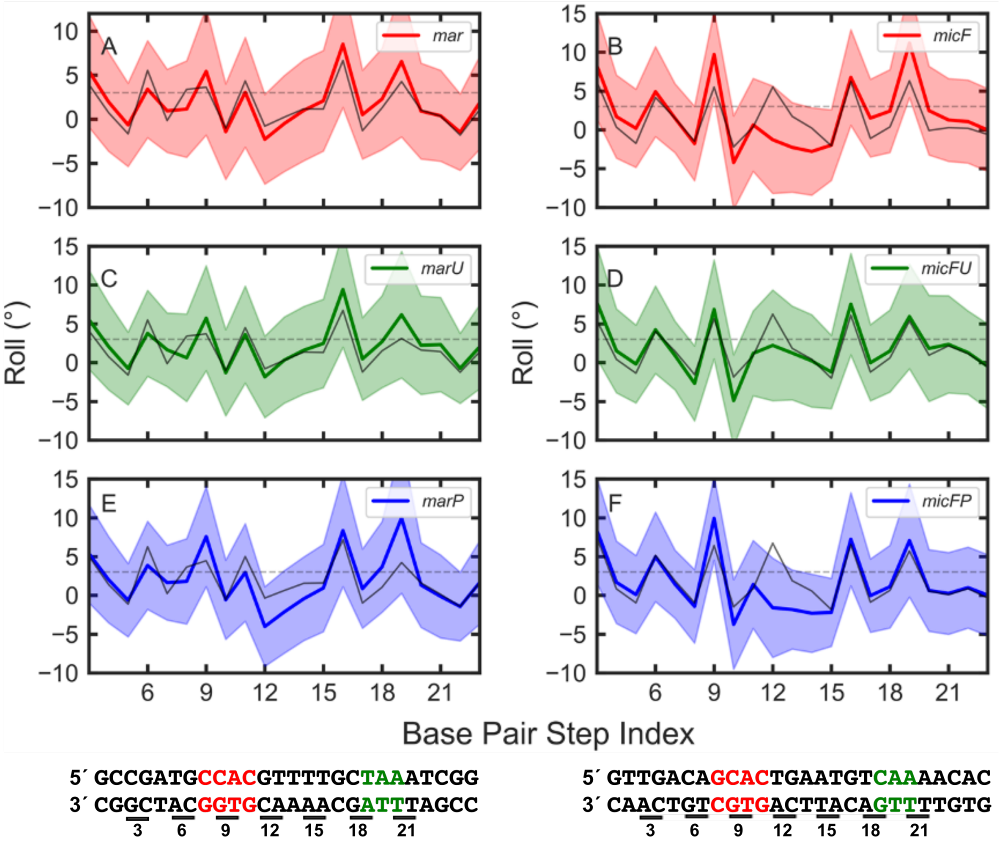

Figure S31. Roll angle (°) between two base pairs in one base pair step reference frame, in simulations of the (**A**) *mar*, (**B**) *marU*, (**C**) *marP*, (**D**) *micF*, (**E**) *micFU* and (**F**) *micFP* promoters, in complex with Rob. The solid and shaded lines denote average values and standard deviations per base pair step, averaged over all replicas for each system. Black solid lines denote average values for the corresponding free DNA sequences. Dashed horizontal lines refer to standard B-DNA parameters. The terminal two base pair steps on each end of the DNA strand were not analyzed to avoid end fraying effects.

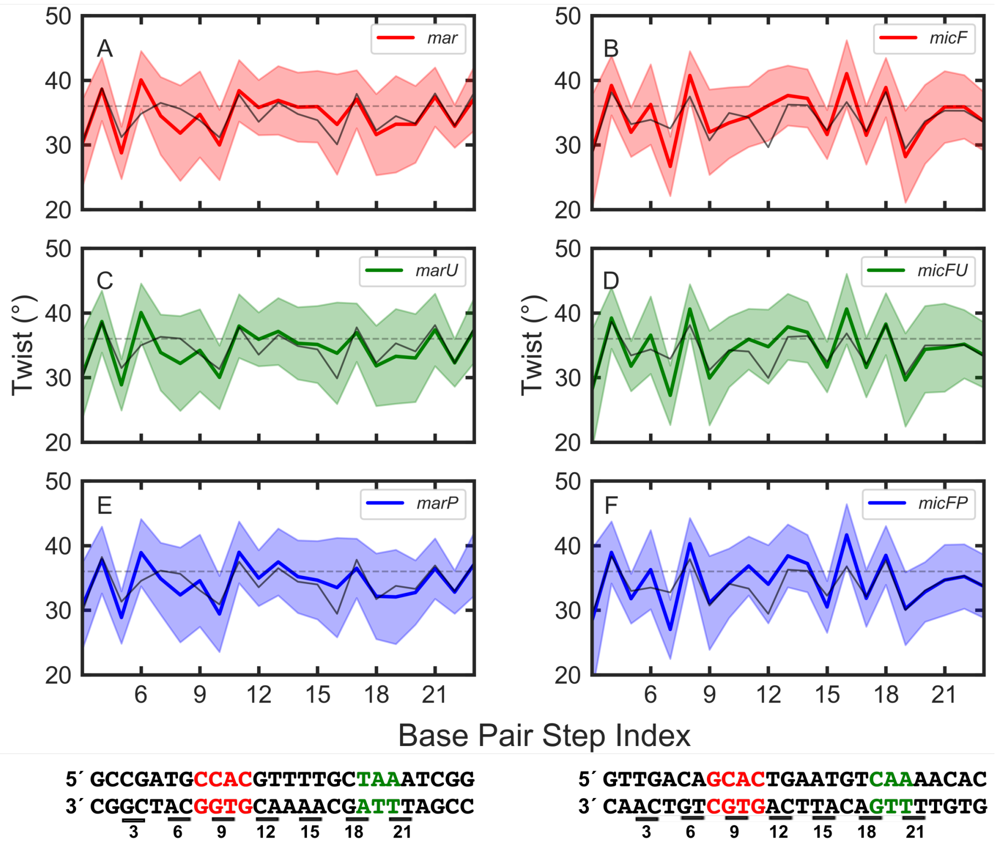

Figure S32. Twist angle (°) between two base pairs in one base pair step reference frame, in simulations of the (**A**) *mar*, (**B**) *marU*, (**C**) *marP*, (**D**) *micF*, (**E**) *micFU* and (**F**) *micFP* promoters, in complex with MarA. The solid and shaded lines denote average values and standard deviations per base pair step, averaged over all replicas for each system. Black solid lines denote average values for the corresponding free DNA sequences. Dashed horizontal lines refer to standard B-DNA parameters. The terminal two base pair steps on each end of the DNA strand were not analyzed to avoid end fraying effects.

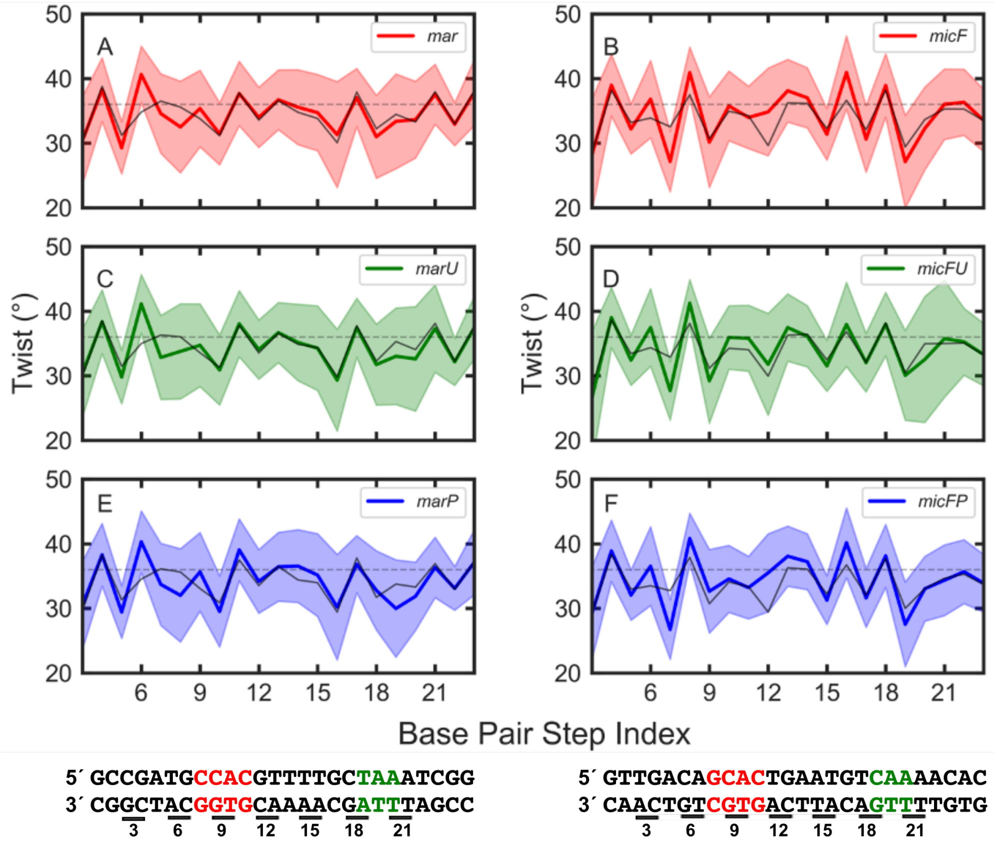

Figure S33. Twist angle (°) between two base pairs in one base pair step reference frame, in simulations of the (**A**) *mar*, (**B**) *marU*, (**C**) *marP*, (**D**) *micF*, (**E**) *micFU* and (**F**) *micFP* promoters, in complex with Rob. The solid and shaded lines denote average values and standard deviations per base pair step, averaged over all replicas for each system. Black solid lines denote average values for the corresponding free DNA sequences. Dashed horizontal lines refer to standard B-DNA parameters. The terminal two base pair steps on each end of the DNA strand were not analyzed to avoid end fraying effects.

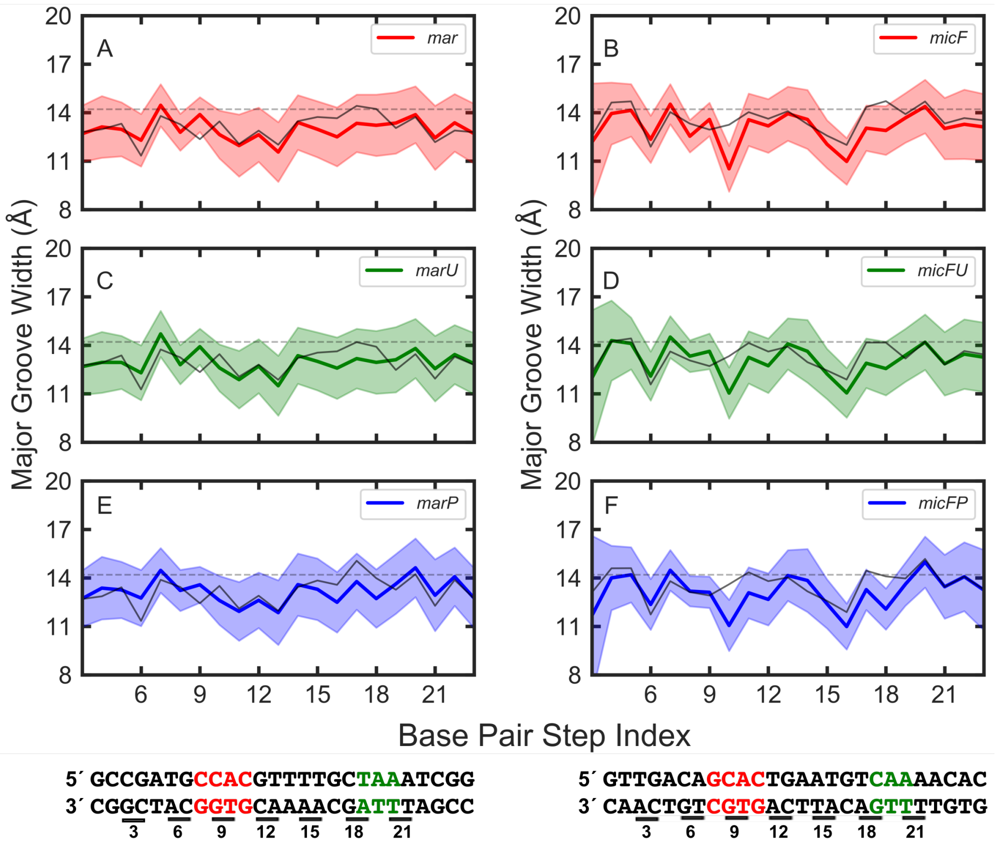

Figure S34. Major groove width (Å) between two base pairs in one base pair step reference frame, in simulations of the (**A**) *mar*, (**B**) *marU*, (**C**) *marP*, (**D**) *micF*, (**E**) *micFU* and (**F**) *micFP* promoters, in complex with MarA. The solid and shaded lines denote average values and standard deviations per base pair step, averaged over all replicas for each system. Black solid lines denote average values for the corresponding free DNA sequences. Dashed horizontal lines refer to standard B-DNA parameters. Note that 5.8 Å was subtracted from the values to take into account the van der Waals radii of the phosphate groups. The terminal two base pair steps on each end of the DNA strand were not analyzed to avoid end fraying effects.

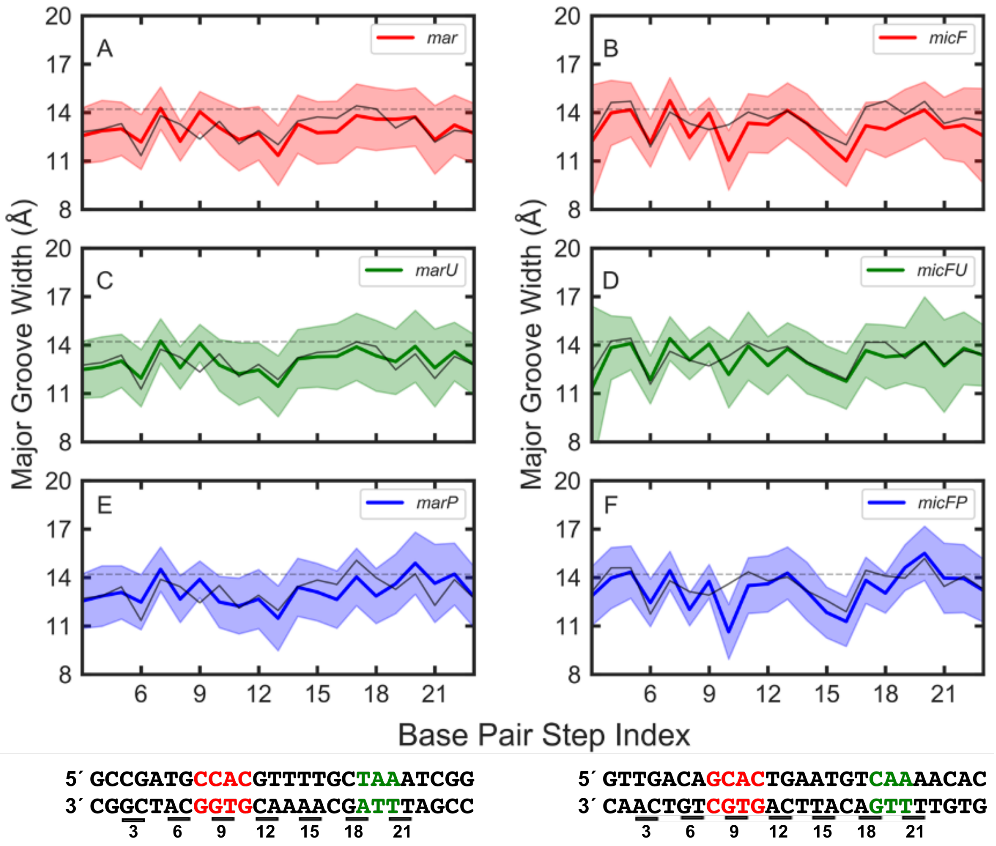

Figure S35. Major groove width (Å) between two base pairs in one base pair step reference frame, in simulations of the (**A**) *mar*, (**B**) *marU*, (**C**) *marP*, (**D**) *micF*, (**E**) *micFU* and (**F**) *micFP* promoters, in complex with Rob. The solid and shaded lines denote average values and standard deviations per base pair step, averaged over all replicas for each system. Black solid lines denote average values for the corresponding free DNA sequences. Dashed horizontal lines refer to standard B-DNA parameters. Note that 5.8 Å was subtracted from the values to take into account the van der Waals radii of the phosphate groups. The terminal two base pair steps on each end of the DNA strand were not analyzed to avoid end fraying effects.

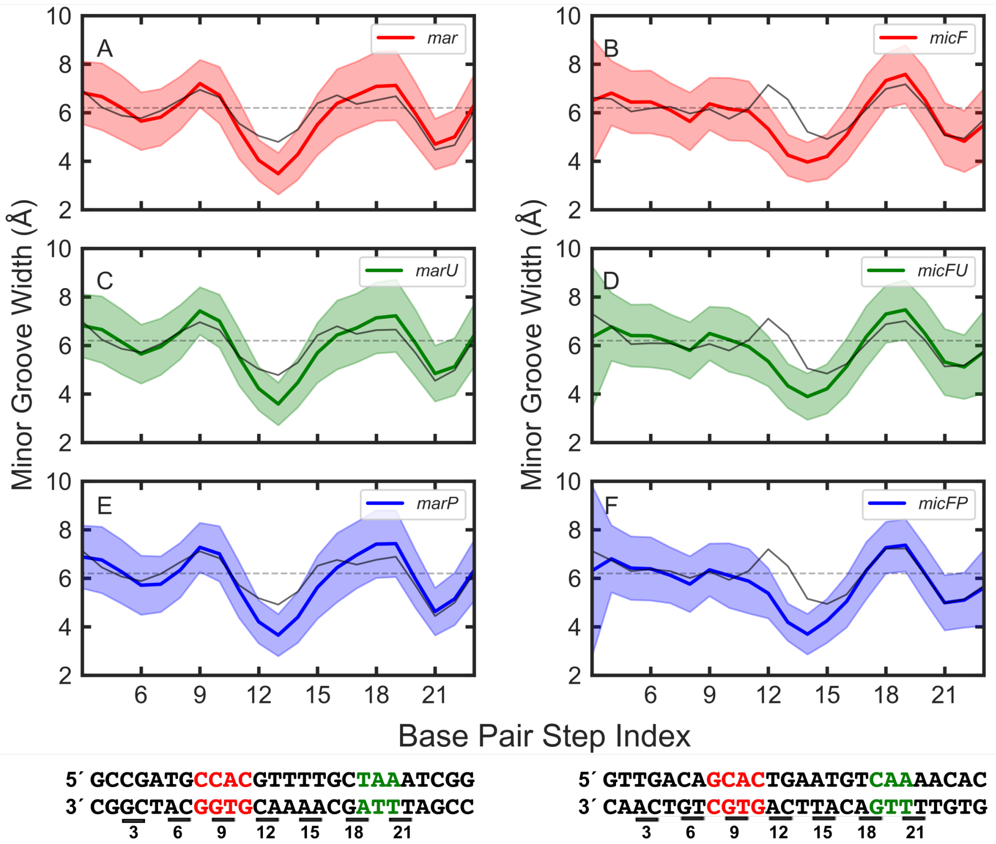

Figure S36. Minor groove width (Å) between two base pairs in one base pair step reference frame, in simulations of the (**A**) *mar*, (**B**) *marU*, (**C**) *marP*, (**D**) *micF*, (**E**) *micFU* and (**F**) *micFP* promoters, in complex with MarA. The solid and shaded lines denote average values and standard deviations per base pair step, averaged over all replicas for each system. Black solid lines denote average values for the corresponding free DNA sequences. Dashed horizontal lines refer to standard B-DNA parameters. Note that 5.8 Å was subtracted from the values to take into account the van der Waals radii of the phosphate groups. The terminal two base pair steps on each end of the DNA strand were not analyzed to avoid end fraying effects.

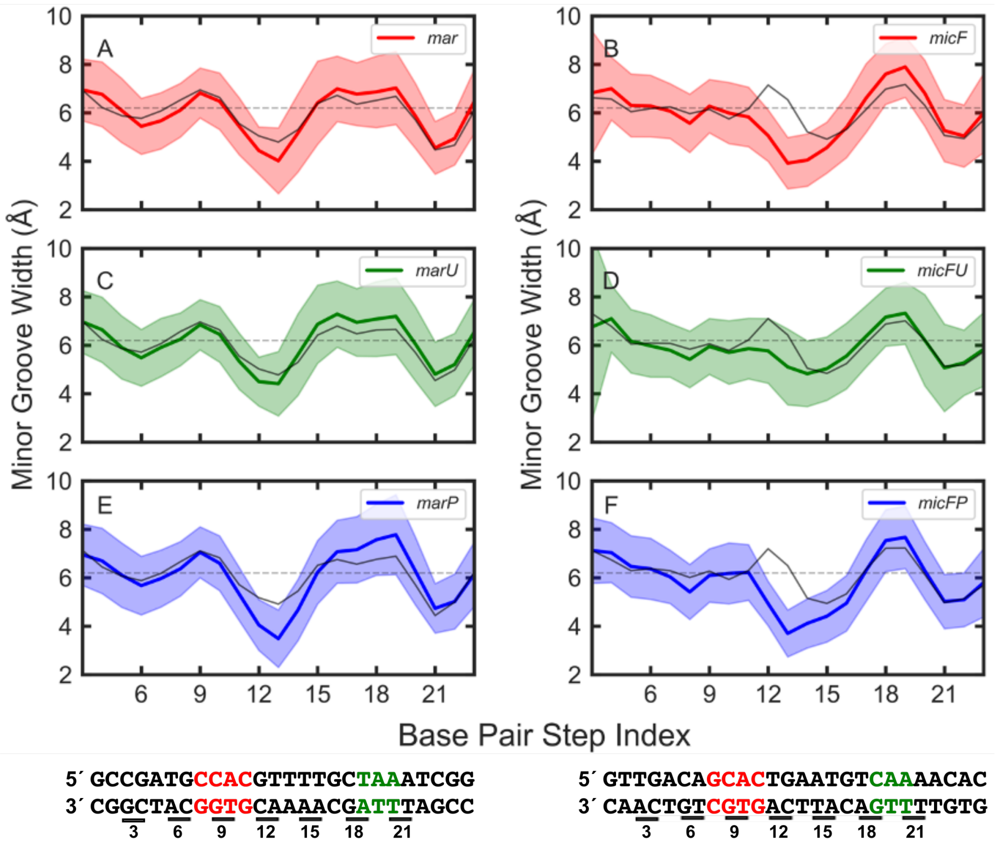

Figure S37. Minor groove width (Å) between two base pairs in one base pair step reference frame, in simulations of the (**A**) *mar*, (**B**) *marU*, (**C**) *marP*, (**D**) *micF*, (**E**) *micFU* and (**F**) *micFP* sequences, in complex with Rob. The solid and shaded lines denote average values and standard deviations per base pair step, averaged over all replicas for each system. Black solid lines denote average values for the corresponding free DNA sequences. Dashed horizontal lines refer to standard B-DNA parameters. Note that 5.8 Å was subtracted from the values to take into account the van der Waals radii of the phosphate groups. The terminal two base pair steps on each end of the DNA strand were not analyzed to avoid end fraying effects.

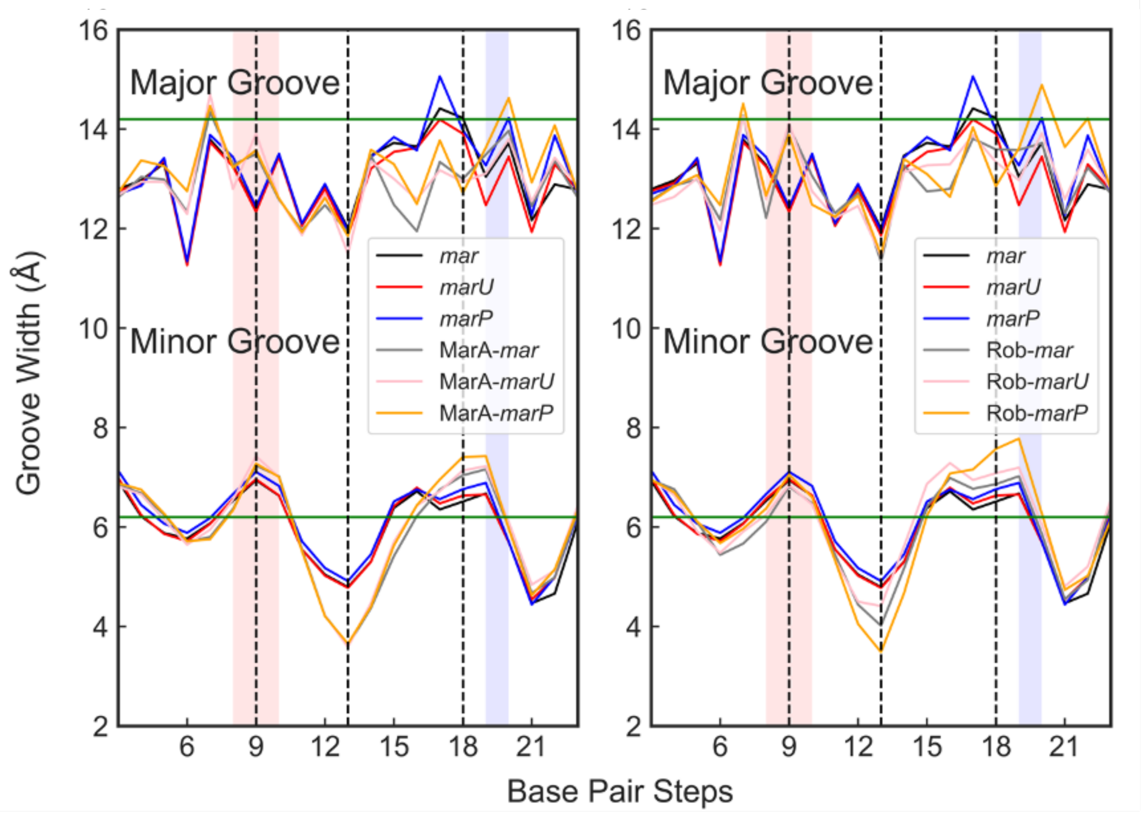

5´ GCCGATGCCACGTTTTGCTAAATCGG
3´ CGGCTACGGTGCAAAACGATTTAGCC
3 6 9 12 15 18 21

Figure S38. Comparison of the major and minor groove widths (Å) over the central 21 base pair steps between *mar* sequences in the absence of DNA and in complex with MarA and Rob. The horizontal green lines refer to the canonical B-DNA major and minor groove widths. The binding domains are highlighted in red (A box) and blue (B box). Note that 5.8 Å was subtracted from the values to take into account the van der Waals radii of the phosphate groups. Finally, the two terminal base pair steps on each end of the DNA strand were not analyzed to avoid end fraying effects.

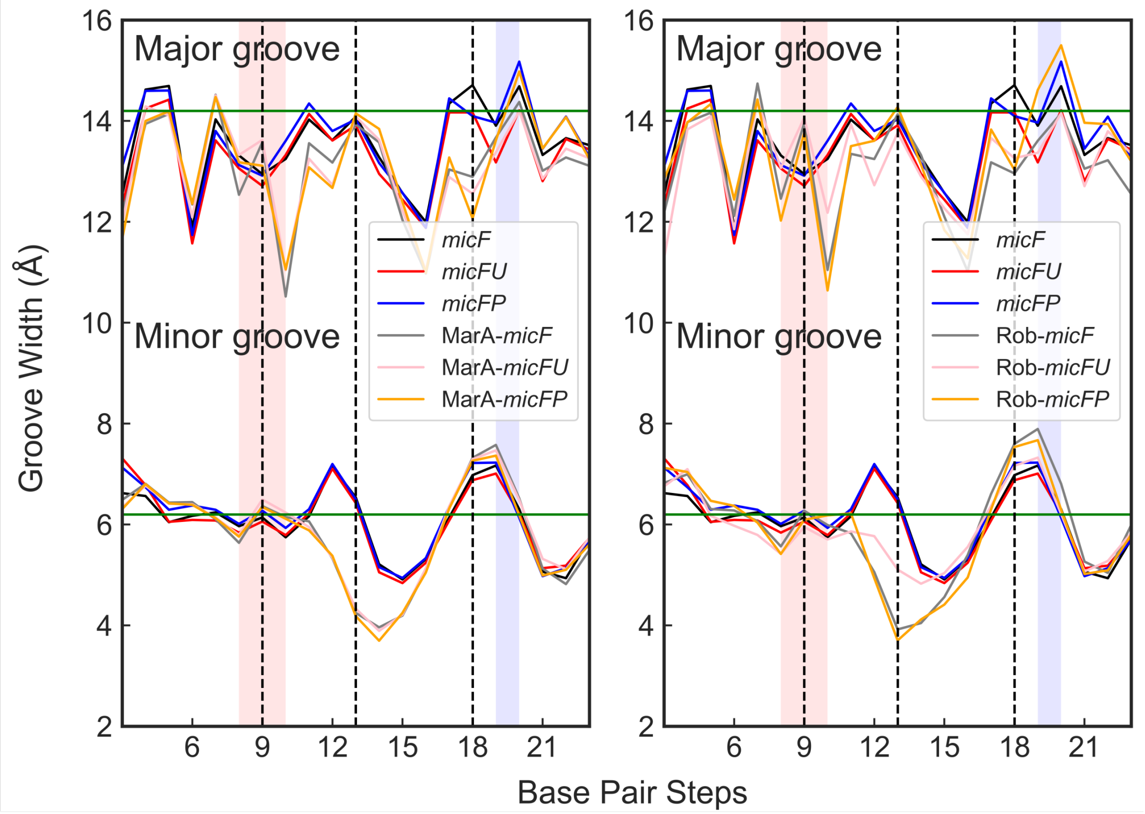

5´ GTTGACAGCACTGAATGTCAAAACAC
3´ CAACTGTCGTGACTTACAGTTTTGTG
3 6 9 12 15 18 21

Figure S39. Comparison of the major and minor groove widths (Å) over the central 21 base pair steps between *micF* sequences in the absence of DNA and in complex with MarA and Rob. The horizontal green lines refer to the canonical B-DNA major and minor groove widths. The binding domains are highlighted in red (A box) and blue (B box). Note that 5.8 Å was subtracted from the values to take into account the van der Waals radii of the phosphate groups. Finally, the two terminal base pair steps on each end of the DNA strand were not analyzed to avoid end fraying effects.

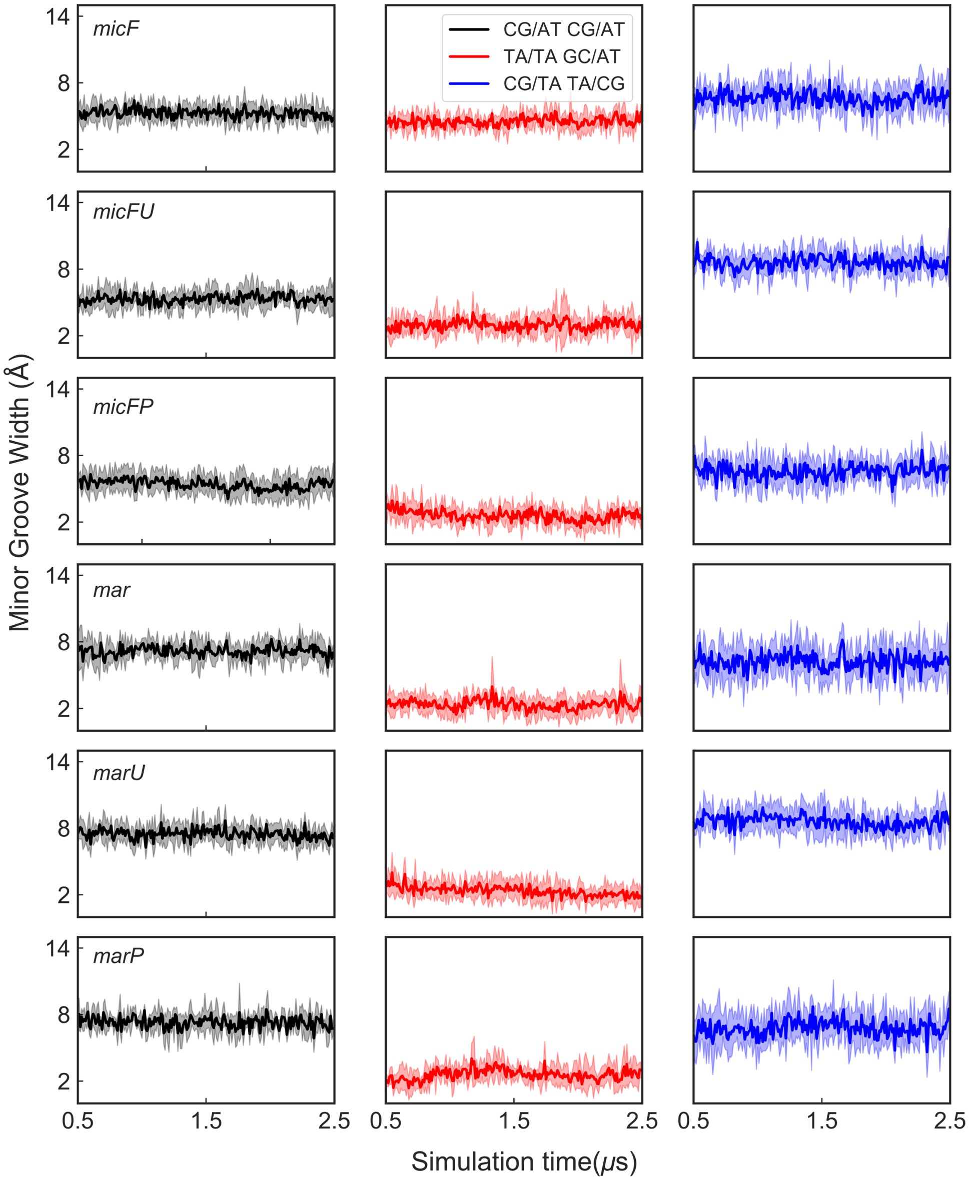

Figure S40. Time evolution of the minor groove width (Å) of three selected base pair steps (CG/AT, TA/TA and CG/TA), displaying major changes in the *micF, micFU, micFP, mar, marU and marP* complexes. The data was extracted from of 5 x 2.5 μs MD simulations of MarA-DNA complexes and collected every 100 ps. The solid lines denote average values over all replicas, and the shaded lines show the standard deviations over the different individual replicas.

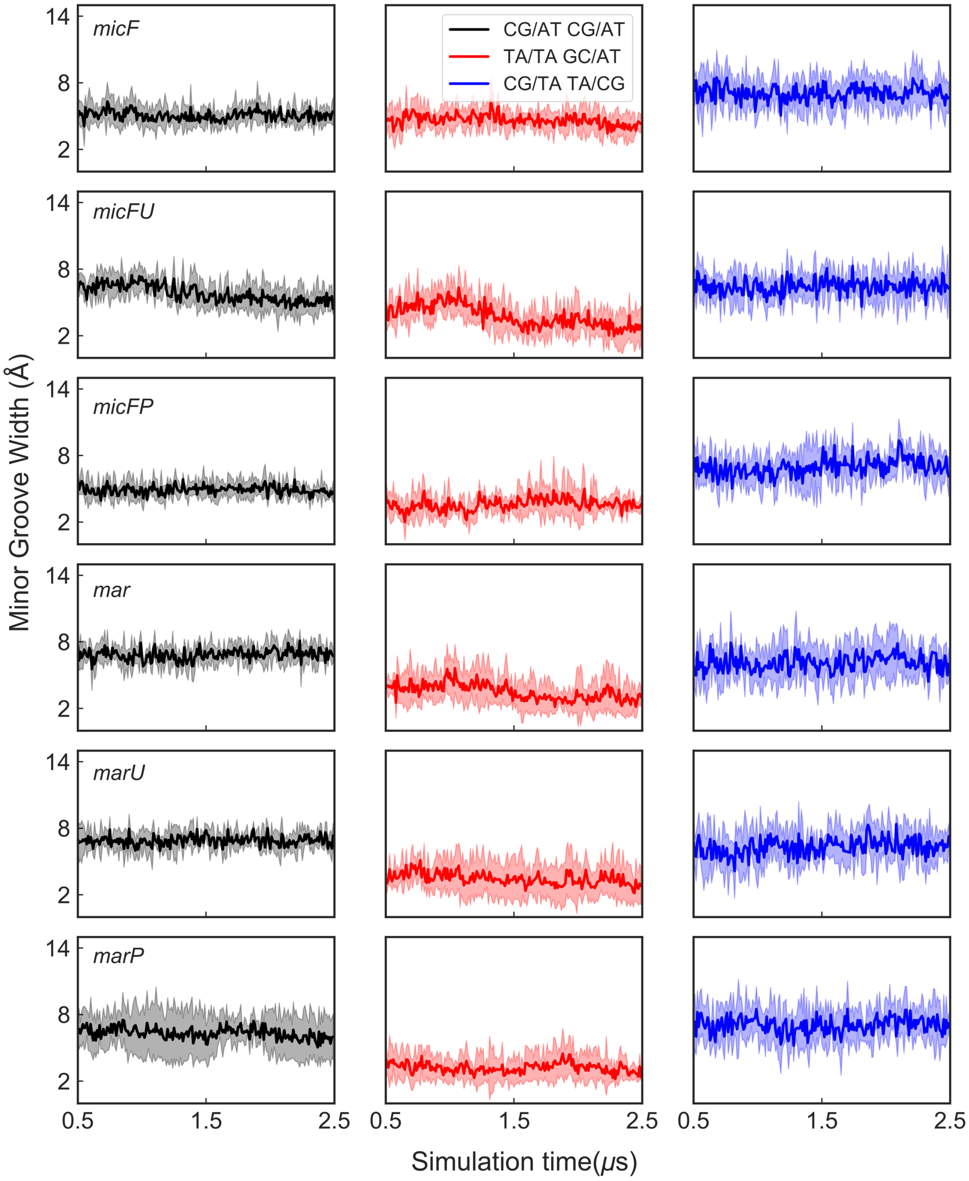

Figure S41. Time evolution of the minor groove width (Å) of three selected base pair steps (CG/AT, TA/TA and CG/TA), displaying major changes in the *micF, micFU, micFP, mar, marU and marP* complexes. The data was extracted from of 5 x 2.5 μs MD simulations of Rob-DNA complexes, with data collected every 100 ps. The solid lines denote average values over all replicas, and the shaded lines show the standard deviations over the different individual replicas.

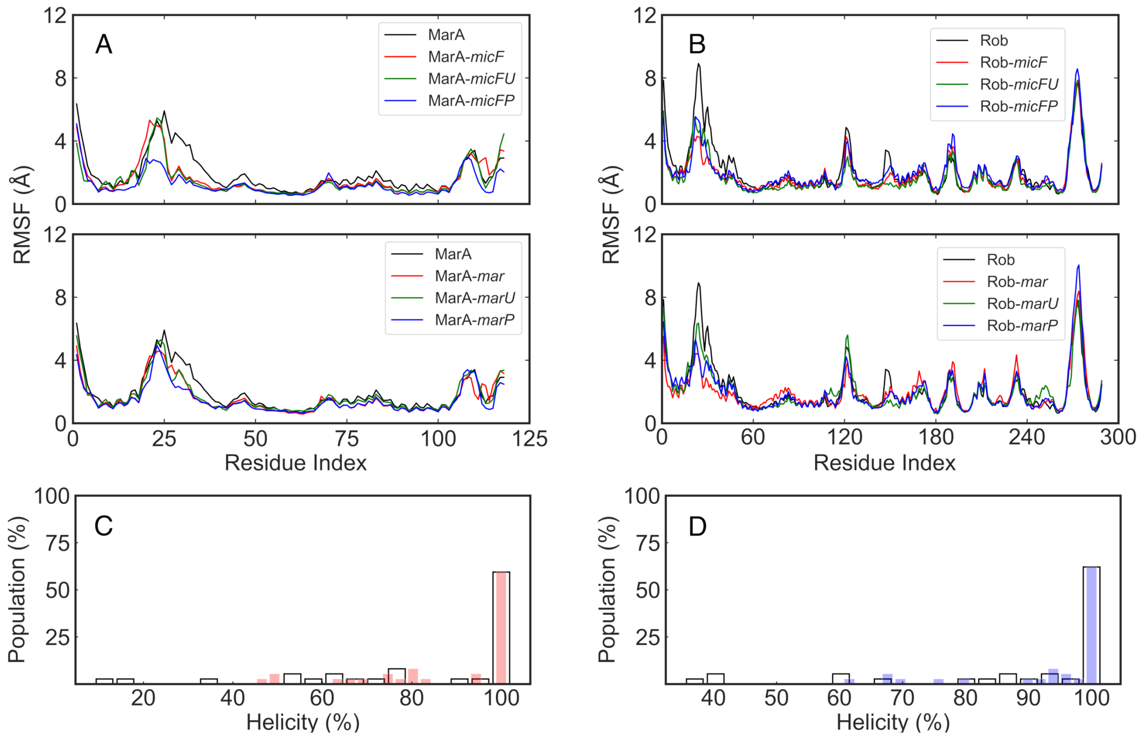

Figure S42. (**A, B**) Comparison of the RMSF (Å) of the C_α atoms of the free MarA and Rob, respectively, with the corresponding mutant promoters *mar, marU, marP, micF, micFU* and *micFP*, calculated over five independent 2.5 μs MD trajectories per system (**Table S3**). Shown here are also the population distributions of the % of helicity of the helices in the HTH motifs, calculated based on the number of residues involved in forming an α-helix or other forms of helices (π- and 3_{10}-helix), over five independent 2.5 μs MD simulations of (**C**) free MarA (black lines) and the native MarA-*mar* promoter complex (red bars), and (**D**) free Rob (black lines) and the native Rob-*micF* promoter complex (blue bars). A helicity of 100% is equivalent to 37 residues (Glu31-Arg36, Lys41-Thr52 in one HTH motif, and Gln91-Phe102, Pro105-Met111 in the second HTH motif) in helical form, as in the crystal structures of MarA and Rob (PDB IDs: 1BL0[3, 4] and 1D5Y[4, 5], respectively). The secondary structure composition was calculated using DSSP[6].

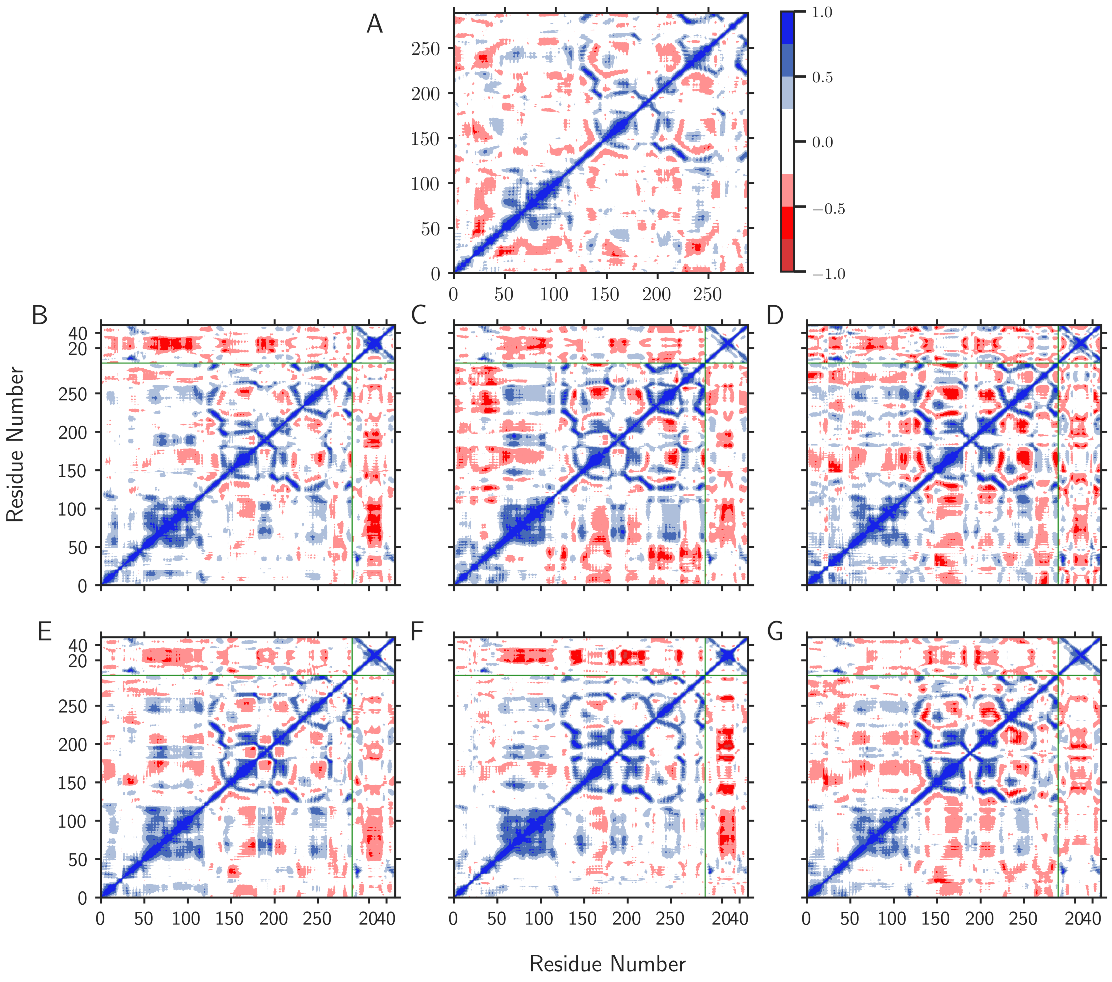

Figure S43. Calculated DCCM plots based on simulations of (**A**) free Rob, as well as Rob in complex with the DNA promoters (**B**) *mar*, (**C**) *marU*, (**D**) *marP*, (**E**) *micF*, (**F**) *micFU*, or (**G**) *micFP*. The plots were calculated with Bio3D[7] by considering only the C_{α}- and P-atoms during 5 x 2.5 μs simulations each of free Rob and the relevant Rob-DNA complexes, respectively.

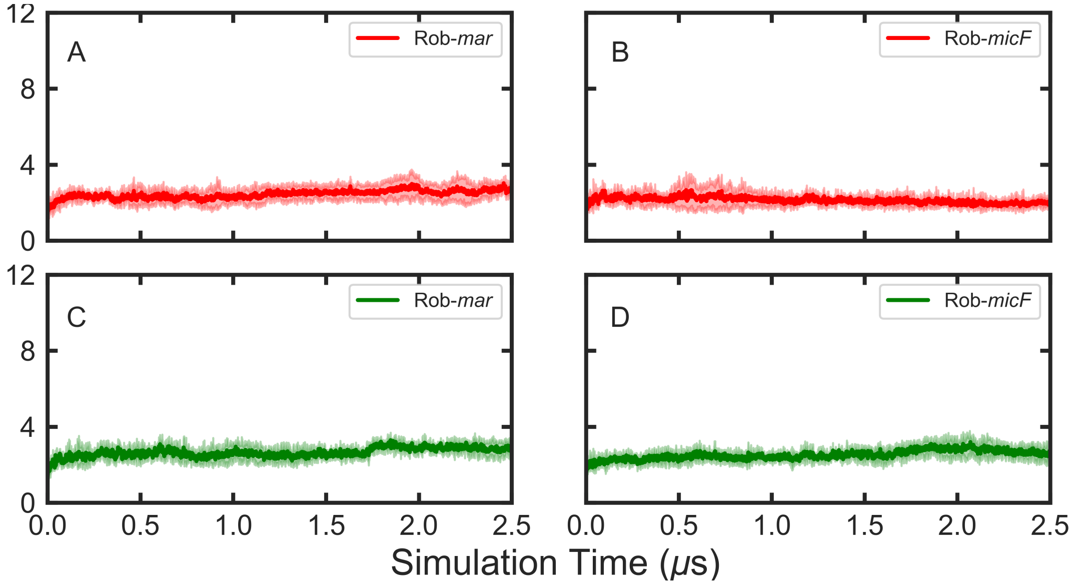

Figure S44. Root mean square deviation (RMSD, Å) of all protein backbone atoms of the Rob C-terminal deleted variant in complex with the (**A**) *mar* and (**B**) *micF* promoters, as well as the loop-deleted variant in complex with the (**C**) *mar* and (**D**) *micF* promoters*,* calculated over 5 independent 2.5 μs molecular dynamics simulations, and relative to the Rob crystal structure. The solid lines denote the average RMSD over all replicas, and the shaded lines show the standard deviations over the different individual replicas.

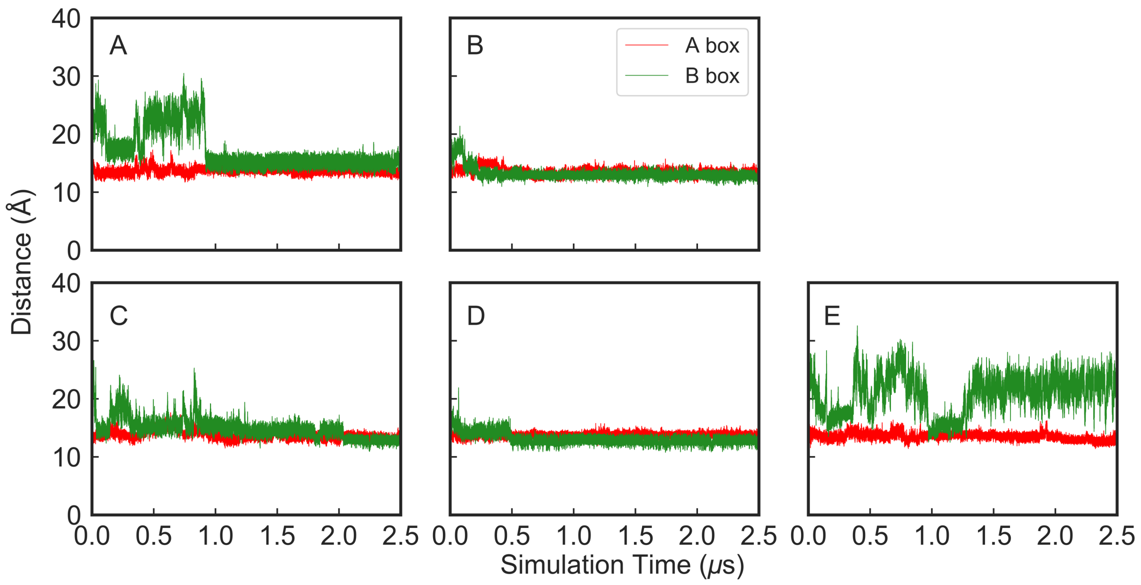

Figure S45. Time evolution of the distances between the helices inserted inside the major groove of the DNA and the base pairs at the A- and B-boxes during 5 x 2.5 μs simulations of the Rob C-terminal domain-deletion variant in complex with the *mar* promoter. The distance analysis was performed using PLUMED v2.5[2] based on snapshots extracted every 10 ps of the simulations.

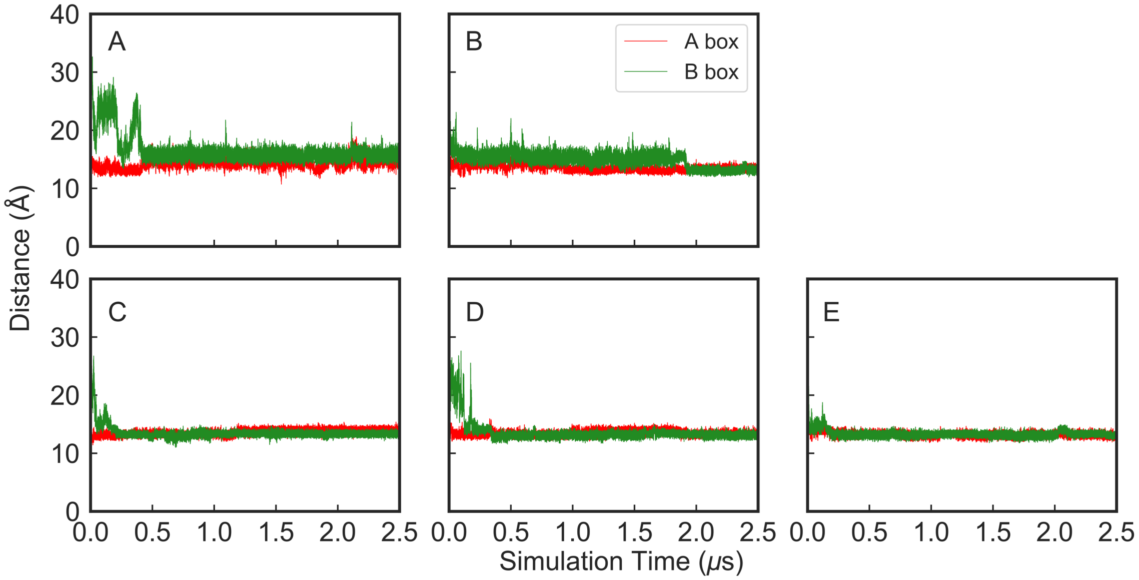

Figure S46. Time evolution of the distances between the helices inserted inside the major groove of the DNA and the base pairs at the A- and B-boxes during 5 x 2.5 μs simulations of the Rob C-terminal domain-deletion variant in complex with the *micF* promoter. The distance analysis was performed using PLUMED v2.5[2] based on snapshots extracted every 10 ps of the simulations.

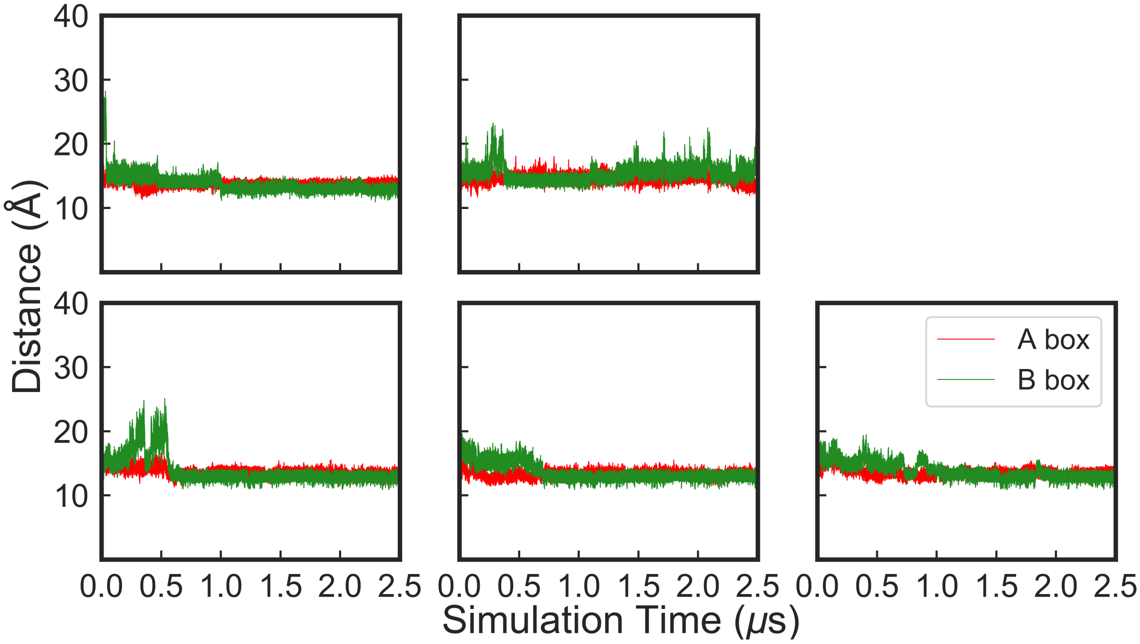

Figure S47. Time evolution of the distances between the helices inserted inside the major groove of the DNA and the base pairs at the A- and B-boxes during 5 x 2.5 μs simulations of the Rob loop-deletion variant in complex with the *mar* promoter. The distance analysis was performed using PLUMED v2.5[2] based on snapshots extracted every 10 ps of the simulations.

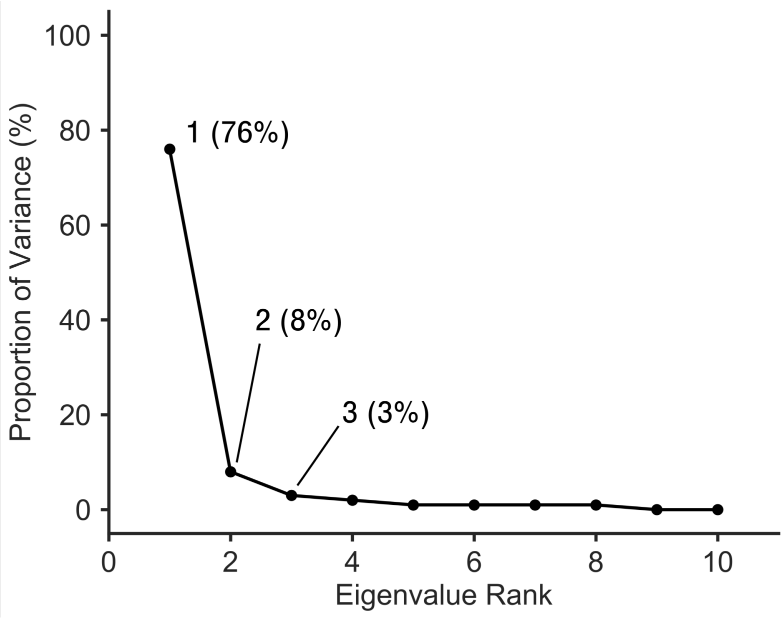

Figure S48. The proportion of the variance described by the first 10 principal components (PC) from PCA performed on the atomic discplacements of the C_α-atoms of residues 1-118 plus the P-atoms of the DNA sequence of all the studied systems (the analysis was performed on all systems simultaneously). The percentage contribution to the variance of the three principal components with the largest eigenvalues are stated on the graph.

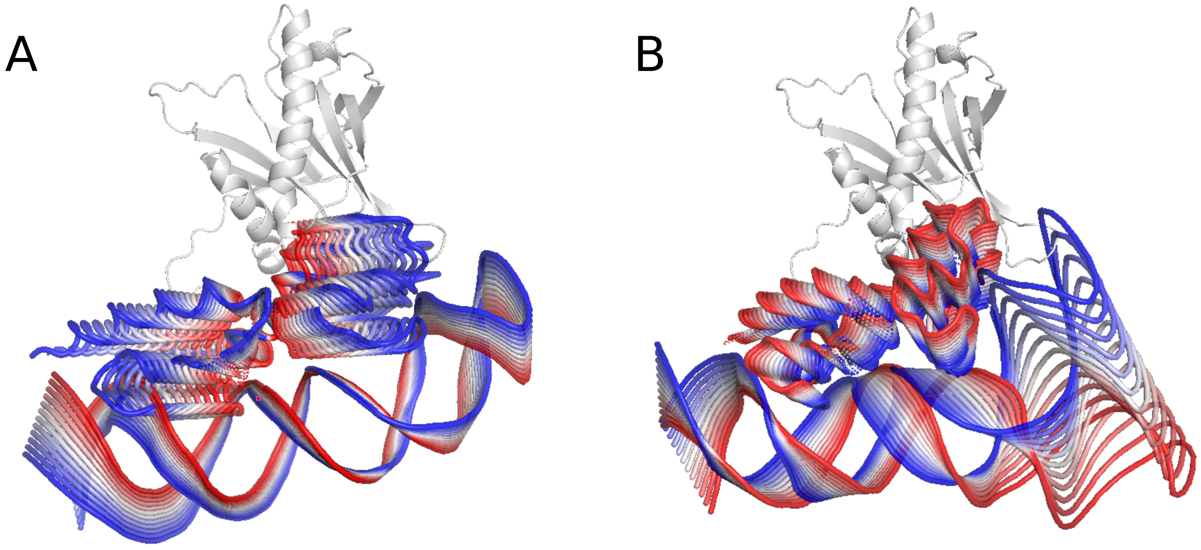

Figure S49. A projection of the first two PCs (PC1 and PC2 in panels **A** and **B**, respectively) from PCA performed on the atomic discplacements of the C_{α}-atoms of residues 1-118 plus the P-atoms of the DNA sequence of all the studied systems (the analysis was performed on all systems simultaneously). The C-terminal domain of Rob is shown in light gray for reference.

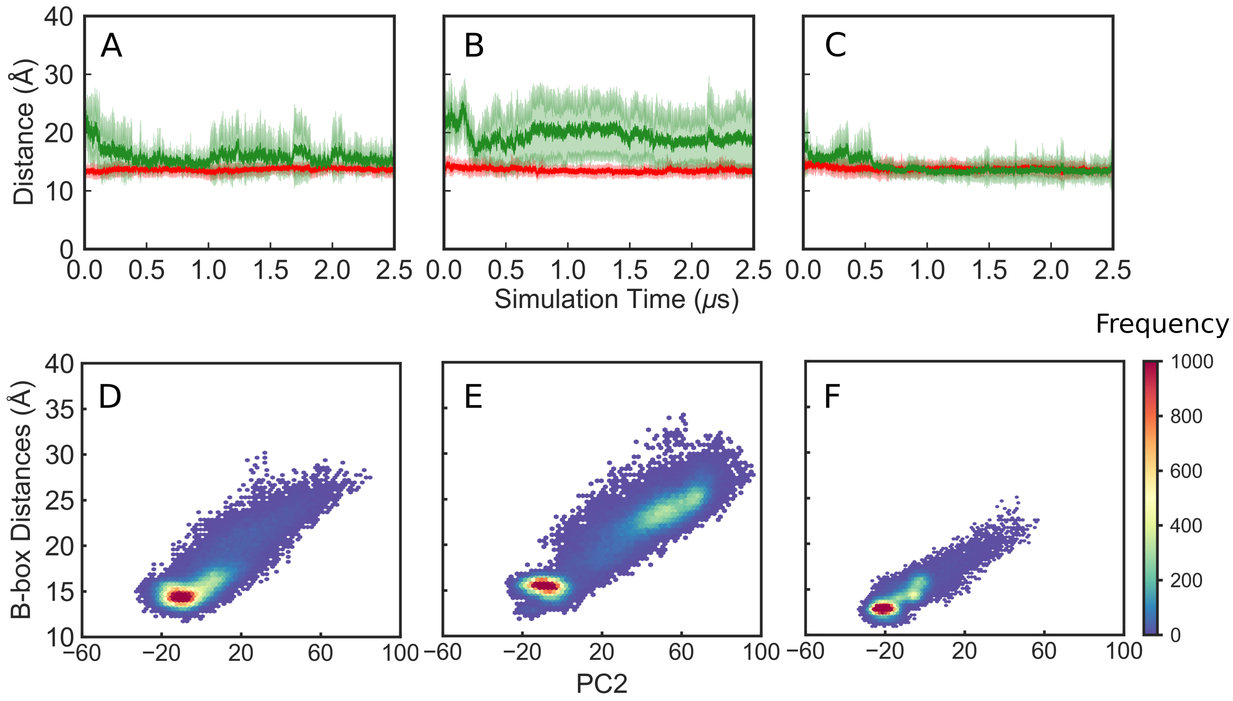

Figure S50. Average distances between the helices inserted inside the major groove and the base pairs at the A- and B-boxes (red and green respectively) during 5 x 2.5 μs simulations of (**A**) MarA, (**B**) Rob and (**C**) the Rob acidic loop-deletion variant in complex with the *mar* promoter. The solid and shaded lines denote the average values and standard deviations over different replicas, respectively. 2D histograms of the B-box distances *vs*. PC2 of the (**D**) MarA, (**E**) Rob and (**F**) the Rob acidic loop-deletion variant in complex with the *mar* promoter.

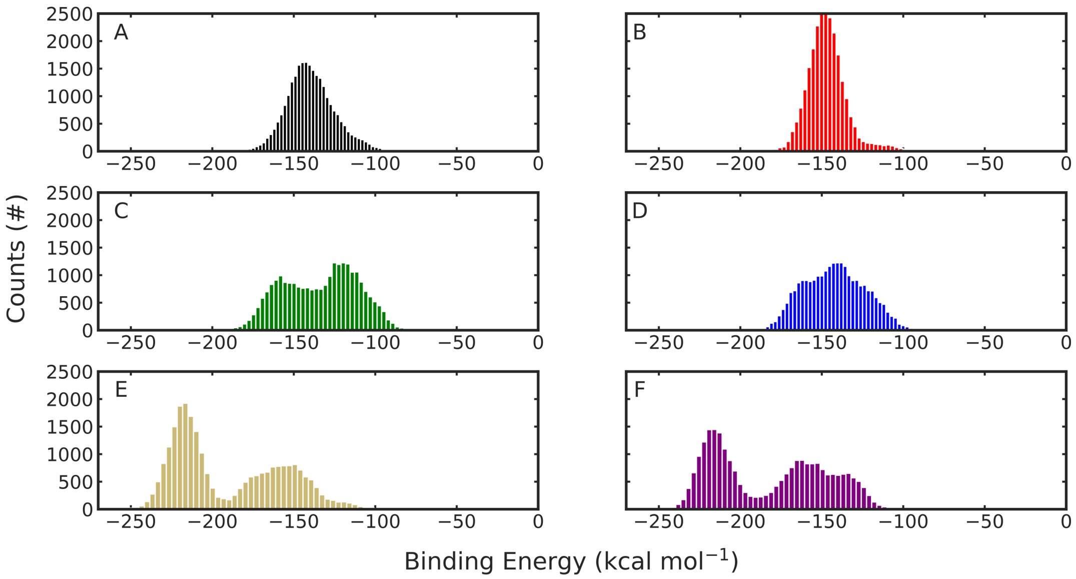

Figure S51. Histogram of estimated relative protein-DNA binding energy estimated for (**A**, **B**) MarA, (**C**, **D**) Rob, and (**E**, **F**) the C-terminal loop deleted Rob variant in complex with the (**A**, **C**, **E**) *mar* and (**B**, **D**, **E**) *micF* promoters, respectively. Energies were calculated using the single-structure MM-PBSA approach[8], as described in the main text, based on data collected every 50 ps of 5 x 2.5 μs molecular dynamics trajectories of each system. Because these calculations do not expliticly include *apo* reference ensembles, conformational changes upon binding may be neglected, and entropic changes may be broadly underestimated. We therefore regard these as qualitative rather than quantitative predictions. However, the C-terminal-loop deletion mutant of Rob displays multiple states, one of which has very similar *micF* binding energies to wild-type Rob. This is consistent with experimental observation.

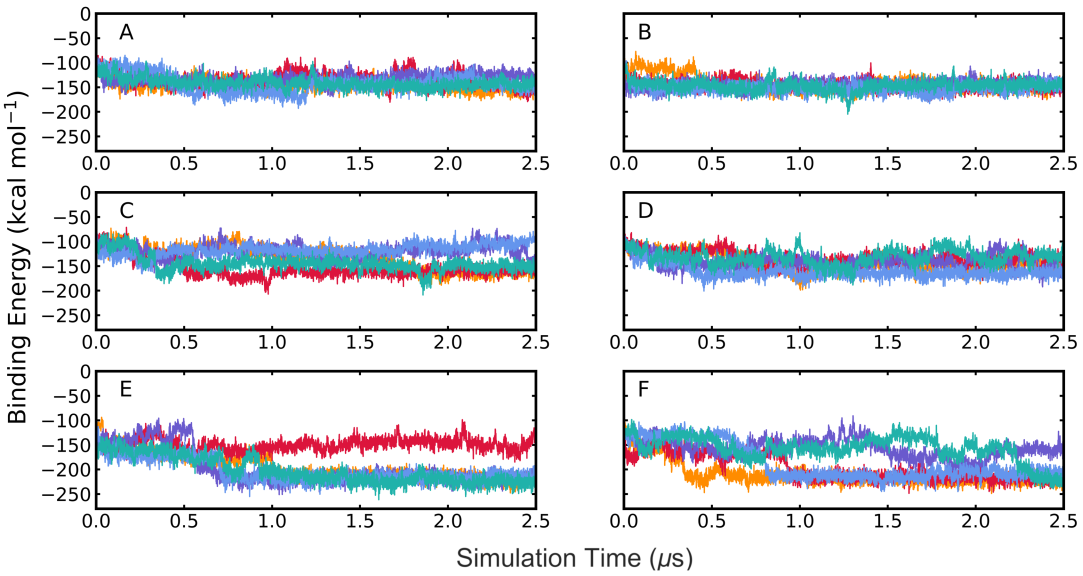

Figure S52. Time evolution of the protein-DNA binding energy estimated for (**A**, **B**) MarA, (**C**, **D**) Rob, and (**E**, **F**) the C-terminal loop deleted Rob variant in complex with the (**A**, **C**, **E**) *mar* and (**B**, **D**, **E**) *micF* promoters, respectively. Estimated energies were calculated using the MM-PBSA approach[8], as described in the main text, based on data collected every 50 ps of 5 x 2.5 μs molecular dynamics trajectories of each system. For the loop-deleted Rob mutant in complex with *micF*, bidirectional transitions were observed between the two binding modes, but the number of transitions sampled was insufficient to estimate equilibrium probabilities of these two modes even with >10 μs of cumulative simulation time.

Supplementary Tables

c. Summary of different DNA sequences studied in this work.[a]

	mar promoter	*micF promoter*
	1 5 10 15 20 25	1 5 10 15 20 25
	5´ GCCGATGCCACGTTTTGCTAAATCGG	5´ GTTGACAGCACTGAATGTCAAAACAC
	3´ CGGCTACGGTGCAAAACGATTTAGCC	3´ CAACTGTCGTGACTTACAGTTTTGTG
	52 48 43 38 33 28	52 48 43 38 33 28

System	DNA Sequence
micF	5′-GTTGACAGCACTGAATGTCAAAACAC-3′<br>3′-CAACTGTCGTGACTTACAGTTTTGTG-5′
micFU	5′-GTTGACAGCACTGAATGTCAAAACAC-3′<br>3′-CAACTGTCGTGACTTACAGUUTTGTG-5′
micFP	5′-GTTGACAGCACTGAATGTCAAAACAC-3′<br>3′-CAACTGTCGTGACTTACAGPTTTGTG-5′
micFA	5′-GTTGACAGGACTGAATGTCAAAACAC-3′<br>3′-CAACTGTCCTGACTTACAGTTTTGTG-5′
mar	5′-GCCGATGCCACGTTTTGCTAAATCGG-3′<br>3′-CGGCTACGGTGCAAAACGATTTAGCC-5′
marU	5′-GCCGATGCCACGTTTTGCTAAATCGG-3′<br>3′-CGGCTACGGTGCAAAACGAUUTAGCC-5′
marP	5′-GCCGATGCCACGTTTTGCTAAATCGG-3′<br>3′-CGGCTACGGTGCAAAACGAPTTAGCC-5′

[a] Shown here is also the base numbering used throughout this work, using the *mar* and *micF* promoters as illustrative examples. Note that P here denotes 5-(1-propynyl)-uracil. Here, A-box residues are shown in red, B-box residues are shown in green, and modified residues are shown in blue. For definitions of the A- and B-boxes, see **Figure 1**.

Table S2. Binding affinities (K_{DNA}, nM) for the binding of modified promoter sequences to MarA and Rob.[a]

System	MarA	Rob
micF	41.0	6.0
micFU	50.5	6.0
micFP	78.0	5.4
mar	43.0	4.4
marU	61.5	5.7
marP	72.0	5.5

[a] This data was originally presented in ref. [5]. For experimental details, see ref. [5]. Note that in the case of the *micFA* sequence, the binding affinity to Rob was measured to be <0.01-fold that of the binding affinity of micF to Rob[9].

Table S3. Summary of all simulations performed in this work.[a]

**Systems**		**Individual Simulations (μs)**	**Total Simulation Time (μs)**
Free DNA	*mar*	3 × 2.5	7.5
	marU	3 × 2.5	7.5
	marP	3 × 2.5	7.5
	micF	3 × 2.5	7.5
	micFU	3 × 2.5	7.5
	micFP	3 × 2.5	7.5
Free MarA		5 × 2.5	12.5
Free Rob		5 × 2.5	12.5
MarA Complexes	*mar*[b]	5 × 2.5	12.5
	mar	5 × 2.5	12.5
	marU	5 × 2.5	12.5
	marP	5 × 2.5	12.5
	micF	5 × 2.5	12.5
	micFU	5 × 2.5	12.5
	micFP	5 × 2.5	12.5
	micFA	5 × 2.5	12.5
Rob Complexes	*mar*	5 × 2.5	12.5
	marU	5 × 2.5	12.5
	marP	5 × 2.5	12.5
	micF	5 × 2.5	12.5
	micFU	5 × 2.5	12.5
	micFP	5 × 2.5	12.5
	micFA	5 × 2.5	12.5
Rob Loop Deletion	*mar*	5 × 2.5	12.5
	micF	5 × 2.5	12.5
Rob C-terminal domain Deletion	*mar*	5 × 2.5	12.5
	micF	5 × 2.5	12.5
		**Total simulation time:**	**307.5**

[a] Summary of the number of individual trajectories studied per system, leading to a cumulative total simulation time of 307.5 μs over all systems. [b] Simulation started from PDB ID: 1BL0[3].

Table S4. Starting complexes for the MD simulations performed in this work.[a]

Systems		Water Molecules[a]	Sodium Ions[a]	Chloride Ions[a]
Free DNA	*mar*	17910	106	56
	marU	17920	106	56
	marP	17913	106	56
	micF	17921	106	56
	micFU	17923	106	56
	micFP	17916	106	56
Free MarA		5687	20	22
Free Rob		11366	41	40
MarA Complexes	*mar*	18528	108	60
	marU	18528	108	60
	marP	18620	108	60
	micF	18526	108	60
	micFU	18528	108	60
	micFP	18525	108	60
	micFA	18528	108	60
	mar[b]		108	60
Rob Complexes	*mar*	17787	112	61
	marU	17774	112	61
	marP	17765	112	61
	micF	17705	112	61
	micFU	17685	112	61
	micFP	17692	112	61
	micFA	17703	112	61
	mar[c]	19262	109	61
	micF[c]	19215	109	61
	mar[d]	18140	109	61
	micF[d]	18094	109	61

[a] Total number of water molecules, sodium ions and chloride ions in the system. [b] Crystallographic starting conformation from PDB ID: 1BL0[3]. [c] C-terminal domain truncated complexes. [d] Acidic loop truncated complex.

Table S5. Partial charges and modified force field parameters for non-standard nucleobases.[a]

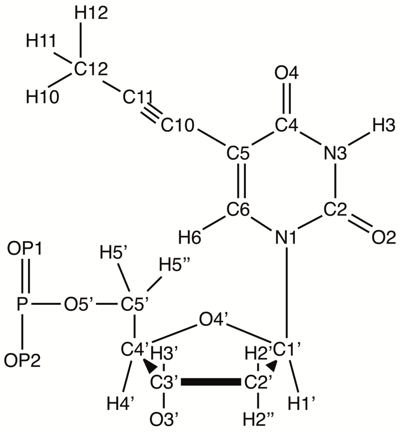			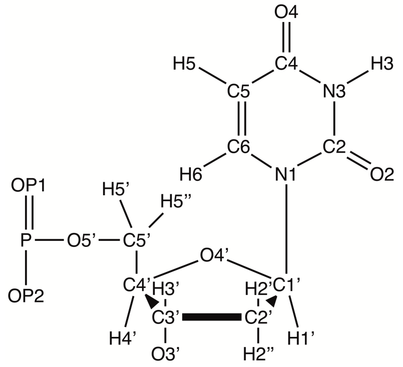		
**5-(1-propynyl-uracil) nucleotide**			**Uracil nucleotide**		
**Name**	**Type**	**Partial Charge**	**Name**	**Type**	**Partial Charge**
P	P	1.1659	P	P	1.1659
OP1	O2	-0.7761	OP1	O2	-0.7761
OP2	O2	-0.7761	OP2	O2	-0.7761
O5’	OS	-0.4954	O5’	OS	-0.4954
C5’	CI	-0.0069	C5’	CI	-0.0069
H5’	H1	0.0754	H5’	H1	0.0754
H5’’	H1	0.0754	H5’’	H1	0.0754
C4’	CT	0.1629	C4’	CT	0.1629
H4’	H1	0.1176	H4’	H1	0.1176
O4’	OS	-0.3691	O4’	OS	-0.3691
C1’	CT	0.0680	C1’	CT	0.0750
H1’	H2	0.1804	H1’	H2	0.1804
N1	N*	-0.0471	N1	N*	0.0418
C6	C2	-0.1357	C6	CS	-0.1126
H6	H4	0.3625	H6	H4	0.2180
C5	C2	0.0122	C5	CS	-0.3635
C4	C	0.6454	H5	HA	0.1811
O4	O	-0.5784	C4	C	0.5952
N3	NA	-0.5792	O4	O	-0.5761
H3	H	0.3519	N3	NA	-0.3549
C2	C	0.6329	H3	H	0.3154
O2	O	-0.6030	C2	C	0.4687
C3’	CE	0.0713	O2	O	-0.5477
H3’	H1	0.0985	C3’	CE	0.0713

C2’	CT	-0.0854	H3’	H1	0.0985
H2’	HC	0.0718	C2’	CT	-0.0854
H2’’	HC	0.0718	H2’	HC	0.0718
O3’	OS	-0.5232	H2’’	HC	0.0718
C10	ch	-0.3187	O3’	OS	-0.5232
C11	c1	0.0239			
C12	c3	-0.0696			
H10	hc	0.0587			
H11	hc	0.0587			
H12	hc	0.0587			

**Bonds**		
Bond type	k_r [b]	r_{eq} [c]
ch-C2	422.6	1.4257

**Angles**		
Angle type	k_θ [b]	θ_{eq} [c]
c1-ch-C2	57.0	178.61
ch-C2-C	65.0	117.88
ch-C2-C2	65.0	125.79

**Dihedrals**			
Dihedral type	n [d]	$V_n/2$ [e]	γ [f]
c1-ch-C2-C	2	0.00	180
c1-ch-C2-C2	2	0.00	180
ch-C2-C-O	4	8.70	180
ch-C2-C-NA	4	8.70	180
ch-C2-C2-H4	4	26.60	180
ch-C2-C2-N*	4	26.60	180

**Impropers**		
Improper type	$V_n/2$ [e]	γ [f]
C-C2-C2-ch	1.10	180

[a] All other force field parameters were directly taken from either the ff14SB[10] or GAFF[11] force fields. Standard AMBER atom types are described using capital letters while GAFF atom types are described using lowercase letters. [b] Force constant for bond or angle in (kcal mol^{-1} Å^{-2}) or (kcal mol^{-1} rad^{-2}). [c] Equilibrium distance or angle in (Å) or (°). [d] Periodicity of the torsion angle. [e] Torsion potential in (kcal mol^{-1}). [f] Phase offset in (°).

Table S6. Stability of A-Box hydrogen-bonding interactions between MarA and all studied DNA sequences over the last 2 μs of 5 independent 2.5 μs MD simulations.[a]

MarA	DNA	*mar*	*marU*	*marP*	*micF*	*micFU*	*micFP*	*micFA*
Ser34 OG	G7 OP	85	95	90	93	95	95	58
Ser34 N	G7 OP	84	86	80	87	87	87	52
Tyr33 OH	T6 OP	68	78	73	69	70	68	50
His37 NE2	T6 OP	58	69	58	53	52	52	39
Ser49 OG	G42 OP	54	52	44	58	58	51	45
Gly51 N	G42 OP	39	28	35	46	46	37	-
Gln52 N	G42 OP	36	35	23	39	46	43	22

[a] The first 500 ns of each trajectory were discarded as equilibration. Values shown as % of simulation time.

Table S7. Stability of B-Box hydrogen-bonding interactions between MarA and all studied DNA sequences over the last 2 μs of 5 independent 2.5 μs MD simulations.[a]

MarA	DNA	*mar*	*marU*	*marP*	*micF*	*micFU*	*micFP*	*micFA*
Thr87 OG1	G17 OP	25	34	18	14	15	18	22
Thr91 OG1	T16 OP	25	21	26	34	46	54	14
Asn94 ND	T16 OP	20	18	18	33	23	44	41
Glu83 N	G17 OP	17	21	9	-	-	-	-
His101 NE2	T32 OP	-	-	-	33	27	36	24

[a] The first 500 ns of each trajectory were discarded as equilibration. Values shown as % of simulation time.

Table S8. Stability of A-Box hydrogen-bonding interactions between MarA/Arg40 and all studied DNA sequences over the last 2 μs of 5 independent 2.5 μs MD simulations.[a]

Arg40	DNA	*mar*	*marU*	*marP*	*micF*	*micFU*	*micFP*	*micFA*
NH1	G7 O6/ G8 O6	28	29	38	43	34	35	-
NH2	G7 O6/ G8 O6	9	9	11	41	44	43	-
NH1	G45 O6	25	26	19	-	-	-	-
NH2	G45 O6	19	25	18	-	-	-	-
NH2	G44 O6	17	21	13	25	18	20	-
NH1	G44 O6	-	-	-	14	16	15	-
NH1	G44 N7				11	14	12	-
NH1	A7 OP							17
NH2	A7 OP							16
NH2	C6 OP							11
NH2	G8 N7							14

[a] The first 500 ns of each trajectory were discarded as equilibration. Values shown as % of simulation time.

Table S9. Stability of B-Box hydrogen-bonding interactions between MarA/Arg90 and all studied DNA sequences over the last 2 μs of 5 independent 2.5 μs MD simulations.[a]

**Arg90**	**DNA**	***mar***	***marU***	***marP***	***micF***	***micFU***	***micFP***	***micFA***
NH2	G17 OP	29	21	31	38	31	66	38
NE	G17 OP	28	27	30	41	33	76	41
NH1	T16 OP	11	-	-	-	-	-	-
NH2	G17 O6	12	11	9	6	7	-	6
NH1	G17 O6	10	14	5		6	-	3
NH1/ NH2	G35 O6/ G34 O6	11	18	6	9	7	-	8

[a] The first 500 ns of each trajectory were discarded as equilibration. Values shown as % of simulation time.

Table S10. Stability of A-Box hydrogen-bonding interactions between Rob and all studied DNA sequences over the last 2 μs of 5 independent 2.5 μs MD simulations.[a]

Rob	DNA	*mar*	*marU*	*marP*	*micF*	*micFU*	*micFP*	*micFA*	*mar*[b]	*micF*[b]	*mar*[c]	*micF*[c]
Ser34 OG	G7 OP	97	90	84	94	93	94	63	96	93	92	92
Ser34 N	G7 OP	85	79	80	88	87	87	61	83	87	85	87
Tyr33 OH	T6 OP	76	73	73	68	69	71	56	76	69	59	68
His37 NE2	T6 OP	65	62	62	49	51	56	46	67	55	65	53
Gln39 NE2	T48 OP	49	34	39	59	29	66	-	45	41	46	40
Gly51 N	G47 OP	-	-	-	39	37	40	-	27	14	32	35

[a] The first 500 ns of each trajectory were discarded as equilibration. Values shown as % of simulation time.

[b] C-terminal domain truncated complexes. [c] Acidic loop truncated complex.

Table S11. Stability of B-Box hydrogen-bonding interactions between Rob and all studied DNA sequences over the last 2 μs of 5 independent 2.5 μs MD simulations.[a]

Rob	DNA	*mar*	*marU*	*marP*	*micF*	*micFU*	*micFP*	*micFA*	*mar*[b]	*micF*[b]	*mar*[c]	*micF*[c]
Thr87 OG1	G17 OP	33	14	23	29	11	66	9	35	52	44	39
Gln86 NE2	T34/ P33 OP	11	-	-	11	-	17	-	5	-	7	4
Gln86 NE2	T34 O4	-	-	-	-	-	-	-	25	45	34	31
Thr99 OG1	T34 OP	-	11	17	26	13	19	-	39	64	59	51
Thr99 OG1	T35 OP	-	-	-	18	-	-	18	19	25	25	14
Lys94 NZ	T15/ A15 OP	27	33	-	27	25	40	27	30	30	30	30
Asp83 N	G17 OP	-	-	-	-	-	21	-	17	18	22	13
Arg90 NE	G17 OP	-	-	-	-	-	-	-	-	23	10	15
Arg104 NH1	T22 OP	-	-	-	-	-	-	-	14	30	-	-
Arg104 NH2	T22 OP	-	-	-	-	-	-	-	25	31	-	14
Arg58 NH2	T16 OP	-	-	-	-	-	-	-	11	19	23	15
Gln85 NE2	T35 OP	-	-	-	-	-	-	-	13	19	23	18
Lys93 NZ	T33 OP	-	-	-	-	-	-	-	23	21	51	42

[a] The first 500 ns of each trajectory were discarded as equilibration. Values shown as % of simulation time.

[b] C-terminal domain truncated complexes. [c] Acidic loop truncated complex.

Table S12. Stability of A-Box hydrogen-bonding interactions between Rob/Arg40 and all studied DNA sequences over the last 2 μs of 5 independent 2.5 μs MD simulations.

**Arg40**	**DNA**	***mar***	***marU***	***marP***	***micF***	***micFU***	***micFP***	***micFA***	***mar*[b]**	***micF*[b]**	***mar*[c]**	***micF*[c]**
NH1	G7 O6/ G8 O6	32	51	32	27	24	50	8	29	30	21	38
NH2	G7 O6/ G8 O6	13	16	16	49	55	41	8	14	43	11	41
NH2	G7 N7/ G8 N7	8	16	7	16	18	8	12	7	28	5	-
NH2	G45 O6	21	10	17	-	-	-	-	21	-	20	-
NH1	G45 O6	16	10	16	-	-	-	-	20	-	19	-
NH2	G44 O6	9	-	10	15	7	25	-	9	19	14	23
NH1	G44 O6	7	-	7	21	20	14	-	6	18	9	13
NH1	G44 N7	-	-	-	-	-	10	-	5	20	5	14

[a] The first 500 ns of each trajectory were discarded as equilibration. Values shown as % of simulation time.

[b] C-terminal domain truncated complexes. [c] Acidic loop truncated complex.

Table S13. Stability of B-Box hydrogen-bonding interactions between Rob/Arg90 and all studied DNA sequences over the last 2 μs of 5 independent 2.5 μs MD simulations.[a]

Arg90	DNA	*mar*	*marU*	*marP*	*micF*	*micFU*	*micFP*	*micFA*	*mar*[b]	*micF*[b]	*mar*[c]	*micF*[c]
NE	G17 OP	-	-	-	27	-	-	24	-	23	10	15
NH2-HH21	G17 OP	-	-	12	22	-	-	16	-	20	12	14
NH2-HH22	G17 OP	20	-	15	-	7	-	-	13	-	5	-
NH1-HH11	G17 OP	-	-	8	-	12	-	-	-	-	-	-
NH1-HH12	G17 OP	7	6	-	-	-	-	-	5	-	5	-
NE	T16 OP/ T18 OP	23	40	15	-	24	-	-	9	-	-	-
NH2-HH21	T16 OP/ T18 OP	23	24	9	-	19	-	-	5	-	-	-
NH2-HH22	T16 OP/ T18 OP	15	-	12	12	-	-	17	9	12	-	5
NH1-HH11	T16 OP/ T18 OP	-	-	-	-	-	-	-	-	-	-	-
NH1-HH12	T16 OP/ T18 OP	-	-	15	-	-	-	6	12	-	-	-
NH1	G17 O6	25	8	15	16	18	21	-	25	31	30	26
NH2	G17 O6	9	-	-	10	-	7	-	11	6	13	10
NH2	G17 N7	11	-	6	8	8	9	-	9	10	12	9
NH1	T18 O4	-	-	-	9	-	34	-	-	-	-	-
NH2	T18 O4	-	-	-	10	-	11	-	-	22	-	11

NH2	G35 O6	-	-	-	-	-	18	-	5	9	6	-
NH1	G35 O6	-	-	-	-	-	-	-	12	15	13	8

[a] The first 500 ns of each trajectory were discarded as equilibration. Values shown as % of simulation time.

[b] C-terminal domain truncated complexes. [c] Acidic loop truncated complex.

Table S14. Average distances between the Trp36 and C8/G8 and C9 ring centroids (R_c), and angles (γ) between normal vectors of each ring plane, defining t-shaped π-π stacking interactions between these residues.[a]

System	Parameters[a]	MarA		Rob	
		**C8/G8**	**C9**	**C8/G8**	**C9**
micF	R_c	5.6 ± 0.4	5.5 ± 0.3	5.7 ± 0.4	5.4 ± 0.4
	γ	68.3 ± 9.9	69.5 ± 11.6	70.6 ± 10.1	72.3 ± 11.6
micFU	R_c	5.6 ± 0.3	5.5 ± 0.3	5.7 ± 0.4	5.4 ± 0.3
	γ	67.9 ± 9.9	68.6 ± 11.4	70.3 ± 10.0	72.1 ± 11.3
micFP	R_c	5.5 ± 0.3	5.5 ± 0.3	5.6 ± 0.4	5.5 ± 0.3
	γ	67.5 ± 9.7	68.2 ± 11.3	69.1 ± 9.9	70.7 ± 11.7
mar	R_c	5.1 ± 0.5	5.4 ± 0.4	5.3 ± 0.7	5.4 ± 0.5
	γ	64.2 ± 11.2	63.4 ± 12.5	70.4 ± 14.6	70.6 ± 14.6
marU	R_c	5.1 ± 0.5	5.4 ± 0.4	5.3 ± 0.6	5.4 ± 0.5
	γ	64.7 ± 12.4	65.5 ± 11.3	70.7 ± 14.9	72.1 ± 15.0
marP	R_c	5.2 ± 0.6	5.5 ± 0.5	5.3 ± 0.5	5.4 ± 0.4
	γ	66.3 ± 12.4	65.8 ± 13.1	67.1 ± 10.9	67.5 ± 12.0
**Average**	R_c	**5.4 ± 0.4**		**5.5 ± 0.5**	
	γ	**66.7 ± 11.4**		**70.3 ± 12.2**	
micFA	R_c	8.0 ± 2.9	9.5 ± 3.0	8.4 ± 3.6	8.9 ± 3.6
	γ	90.8 ± 20.8	90.1 ± 22.8	105.5 ± 23.4	105.5 ± 24.1
mar[b]	R_c	-	-	5.2 ± 0.6	5.5 ± 0.4
	γ	-	-	65.5 ± 11.7	65.4 ± 12.7
micF[b]	R_c	-	-	6.2 ± 0.3	5.5 ± 0.3
	γ	-	-	67.0 ± 9.9	68.9 ± 11.4
mar[c]	R_c	-	-	5.8 ± 0.7	5.6 ± 0.6
	γ	-	-	76.5 ± 17.6	77.2 ± 17.1
micF[c]	R_c	-	-	5.2 ± 0.5	5.4 ± 0.4
	γ	-	-	66.5 ± 11.1	66.0 ± 12.0

[a] A T-shaped π-π stacking interactions between two rings is defined as R_c between 4.5 and 7.5 Å, and γ between 50 and 90° [12-14]. [b] Acidic loop truncated complex. [c] C-terminal domain truncated complexes.

Table S15. MarA-water hydrogen-bonding interactions found in the MarA-*mar* crystal structure, and the stability of the corresponding interactions in our MarA simulated complexes.[a]

MarA	*mar*	*marU*	*marP*	*micF*	*micFU*	*micFP*	*micFA*
Arg90 NH1	74	87	90	115	89	122	125
Lys26 NZ	162	162	150	168	166	156	166
Asn70 ND2	91	82	81	83	80	81	83
Lys43 O	59	59	64	58	57	59	58
Leu22 O	87	99	54	96	85	50	90
Hie116 N	19	15	21	19	17	20	12
Asn107 OD1	112	108	113	116	108	101	105
Arg113 NE	37	44	44	47	41	46	32
Asp16 OD2	138	157	204	137	149	193	184
Thr89 OG1	52	56	53	64	52	57	58
Ser112 OG	131	124	121	131	135	140	134
Lys102 NZ	172	166	174	159	168	159	157
Asn94 ND2	74	82	69	58	67	56	56
Gln86 NE2	74	83	80	82	74	83	83
Ser84 OG	83	79	78	82	84	78	79

[a] Analysis was performed over the last 500 ns of 5 independent 2.5 μs MD simulations, in order to allow the system to have fully equilibrated. All values are shown as a % of the simulation time. Values over 100% correspond to interactions involving more than one water molecule. Note here that as we do not retain the crystallographic water molecules in our simulations, it was not possible to track the original water molecules directly. Therefore, we instead identified the hydrogen-bonding interactions found in the original MarA-*mar* crystal structure between the crystallographic water molecules and either the protein and/or the DNA, and tracked the stability of the corresponding interactions found in our simulated systems. An interaction was considered to correspond to a hydrogen bond if the donor-acceptor distance between any hydrogen-bond donor or acceptor from the protein and/or the DNA and the oxygen of the water molecule was 3.5Å or less.

Table S16. *mar*-water hydrogen-bonding interactions found in the MarA-*mar* crystal structure, and the stability of the corresponding interactions in our MarA-*mar*-like simulated complexes.[a]

MarA crystal	*mar*	*marU*	*marP*
T31 O4	82	82	82
T/U32 OP2	210	208	213
A34 N7	62	74	71
C41 OP1	220	204	176
C18 N4	38	37	38
T19 OP2	244	244	245
T23 O4'	13	14	12

[a] Analysis was performed over the last 500 ns of 5 independent 2.5 μs MD simulations, in order to allow the system to have fully equilibrated. All values are shown as a % of the simulation time. Values over 100% correspond to interactions involving more than one water molecule. Note here that as we do not retain the crystallographic water molecules in our simulations, it was not possible to track the original water molecules directly. Therefore, we instead identified the hydrogen-bonding interactions found in the original MarA-*mar* crystal structure between the crystallographic water molecules and either the protein and/or the DNA, and tracked the stability of the corresponding interactions found in our simulations. An interaction was considered to correspond to a hydrogen bond if the donor-acceptor distance between any hydrogen-bond donor or acceptor from the protein and/or the DNA and the oxygen of the water molecule was 3.5Å or less.

Table S17. MarA-water-*mar* hydrogen-bonding interactions found in MarA-*mar* crystal structure, and the stability of the corresponding interactions in our MarA-*mar* like simulated complexes.[a]

MarA crystal	*mar*	*marU*	*marP*
hr	1		

[a] Analysis was performed over the last 500 ns of 5 independent 2.5 μs MD simulations, in order to allow the system to have fully equilibrated. All values are shown as a % of the simulation time, and as can be seen from this data, this crystallographic interaction is not observed in our simulations. Note here that as we do not retain the crystallographic water molecules in our simulations, it was not possible to track the original water molecules directly. Therefore, we instead identified the hydrogen-bonding interactions found in the original MarA-*mar* crystal structure between the crystallographic water molecules and either the protein and/or the DNA, and tracked the stability of the corresponding interactions found in our simulations. An interaction was considered to correspond to a hydrogen bond if the donor-acceptor distance between any hydrogen-bond donor or acceptor from the protein and/or the DNA and the oxygen of the water molecule was 3.5Å or less.

Table S18. Stability of A-Box water hydrogen-bond bridging interactions between MarA and all studied DNA sequences.[a]

Interaction	***mar***	***marU***	***marP***	***micF***	***micFU***	***micFP***	***micFA***
	60	58		59	52	61	35
Asp3:T/C6	32	38	50	35	31	38	-
Glu25:C/A41	15	11	46	17	-	35	-
Gln39:G42	43	24	28	33	38	23	16
Gln39:T43	33	35	14	50	46	26	20
Gln52:T43	-	21	-	-	-	11	-
Arg55:C/A41	-	21	22	-	-	18	-
Thr2:T/C6	-	-	-	10	-	-	21
Lys43:T43	15	16	16	15	15	17	18
Lys44:A5	17	16	13	12	20	13	14
Lys35:C/A41	19	17	-	27	25	19	12
Arg40:G/C45	10	12	15	-	-	-	-
Arg40:G44	15	12	15	-	14	13	-
Arg40:G/A7	13	12	-	21	23	21	20
Ser34:C/G8	10	14	15	15	14	-	-
Trp36:G42	10	-	-	13	-	11	-
Ser49:T43	-	20	12	-	-	20	-
Hie37:G/A7	-	-	-	17	12	15	-

[a] Analysis was performed over the last 500 ns of 5 independent 2.5 μs MD simulations, in order to allow the system to have fully equilibrated. All values are shown as a % of the simulation time. "-" denotes that the interaction of interest was observed for less than 10% of the simulated time in that system.

Table S19. Stability of B-Box water hydrogen-bond bridging interactions between MarA and all studied DNA sequences.[a]

Interaction	*mar*	*marU*	*marP*	*micF*	*micFU*	*micFP*	*micFA*
	27	36		36	26	-	33
Arg90:G17	19	20	26	-	19	-	-
Arg90:C/T18	22	34	23	-	22	53	48
Thr87:G17	11	31	26	22	12	52	18
Thr91:T16	41	35	23	27	16	55	40
Thr91:G17	-	17	11	-	-	-	12
Arg55:T/A15	31	27	-	31	30	29	25
Asn94:T16	-	24	19	11	14	12	16
Asn94:T/A15	-	-	-	-	-	50	-
Gly81:G17	16	-	-	-	26	-	-
Glu83:G17	24	19	18	15	12	14	13
Glu83:C/T18	17	-	-	-	38	-	-
Lys93:T/U/P33	16	18	18	28	22	29	21
Gln86:T/U32	16	-	12	13	21	-	-
Gln52:T/A15	16	15	12	-	11	-	-

[a] Analysis was performed over the last 500 ns of 5 independent 2.5 μs MD simulations, in order to allow the system to have fully equilibrated. All values are shown as a % of the simulation time. "-" denotes that the interaction of interest was observed less than 10% of the simulated time in that system.

Table S20. Stability of A-Box water hydrogen-bond bridging interactions between Rob and all studied DNA sequences.[a]

Interaction	*mar*	*marU*	*marP*	*micF*	*micFU*	*micFP*	*micFA*	*mar*[b]	*micF*[b]	*mar*[c]	*micF*[c]
	56	59		57	58	61	28	60	59	59	58
Gln39:G42	23	43	39	36	28	55	-	26	58	30	53
Gln39:T43	11	-	16	-	-	14	-	-	-	-	-
Gln39:A41	-	-	-	-	-	-	14	-	-	-	-
Arg55:C/A41	27	20	-	-	28	13	15	37	19	39	-
Gln3:T/C6	23	-	21	-	27	24	15	-	-	-	-
Lys35:C/A41	14	19	21	16	16	22	-	17	23	13	19
Arg40:G/A7	-	-	-	-	24	23	10	-	21	-	19
Arg40:G44	17	10	18	-	-	-	-	15	-	14	-
Arg40:G45	11	15	11	20	20	-	-	12	13	17	-
Hie37:G/A7	-	-	-	21	17	15	-	-	14	-	18
Leu22:C/A41	20	-	-	-	10	11	-	-	-	14	15
Lys43:G44	20	-	14	25	-	-	20	22	12	25	-
Lys43:T43	17	12	16	18	15	-	12	18	10	20	-
Trp36:G42	-	13	16	-	-	15	-	11	12	13	-
Asp25:C/A41	10	10	13	-	-	-	18	-	-	11	29
Gln3:A5	-	12	12	20	-	-	-	26	25	23	23
Ser34:C/G8	-	14	11	13	12	16	-	19	11	-	-

[a] Analysis was performed over the last 500 ns of 5 independent 2.5 μs MD simulations, in order to allow the system to have fully equilibrated. All values are shown as a % of the simulation time. "-" denotes that the interaction of interest was observed less than 10% of the simulated time in that system. [b] C-terminal domain truncated complexes. [c] Acidic loop truncated complex.

Table S21. Stability of B-Box water hydrogen-bond bridging interactions between Rob and all studied DNA sequences.[a]

Interaction	***mar***	***marU***	***marP***	***micF***	***micFU***	***micFP***	***micFA***	***mar*[b]**	***micF*[b]**	***mar*[c]**	***micF*[c]**
	28	20		17	28	29	13	-	23	23	-
	-	-		30	-	-	27	13	-	-	-
Arg90: G/A35	16	-		-	11	-	-	26	-	22	-
Asp83:G17	36	15	27	29	21	31	-	19	43	20	30
Asp83:C/T18	30	21	17	22	26	50	16	48	-	32	46
Arg58:T16	36	15	23	-	30	30	-	37	-	30	35
Arg58:G17	-	-	16	-	-	-	-	30	16	27	15
Lys93:T/U/P33	-	13	21	21	14	30	-	25	16	16	15
Lys93:T/U32	-	-	-	11	-	-	-	16	16	-	-
Thr87:T16	18	-	21	-	15	14	-	-	-	-	-
Thr87:G17	-	-	15	39	-	12	18	13	10	12	19
Asp191:G/C26	-	-	19	-	19	19	-	-	-	-	-
Asp192:T31	29	-	18	14	-	17	-	-	-	-	-
Arg81:G17	48	18	18	-	-	-	-	39	15	25	-
Lys94:T/A15	14	19	17	12	16	15	-	16	19	15	17
Lys94:T16	13	11	15	-	12	13	-	-	-	-	-
Lys94:G17	-	-	-	-	-	-	16	-	-	-	-
Arg55:T/A15	20	17	16	22	37	42	-	49	44	46	43
Gln85:T31	-	21	13	13	24	26	15	46	52	37	49
Gln85:T/U32	13	13	-	15	-	-	-	-	-	-	-
Gln86:G/A35	15	12	-	-	-	-	-	-	-	-	-
Thr89:T/U32	-	12	-	10	16	12	-	29	31	20	27
Ser84:C/T18	-	-	-	21	-	-	12	-	13	-	19
Asp192:A/T30	-	-	14	-	-	-	-	-	-	-	-
Asp192:T/U32	-	11	-	-	-	-	-	-	-	-	-
Glu193:T31	-	-	-	-	-	16	-	-	-	-	-
Thr99:T/U32	-	-	10	15	-	12	-	31	12	20	-
Arg104:T31	-	-	10	-	-	14	-	25	26	-	18

Arg105:T/U32	-	-	-	-	-	-	-	24	14	16	20

[a] Analysis was performed over the last 500 ns of 5 independent 2.5 μs MD simulations, in order to allow the system to have fully equilibrated. All values are shown as a % of the simulation time. "-" denotes that the interaction of interest was observed less than 10% of the simulated time in that system. [b] C-terminal domain truncated complexes. [c] Acidic loop truncated complex.

Table S22. Comparison between the polar contributions to the protein-DNA binding free eneriges obtained using the linear and non-linear versions of the Poisson-Boltmzann equation for the MarA, Rob and the C-terminal loop deleted Rob variant (Rob_{ld}) studied in this work, in complex with the *mar* and *micF* promoters.[a]

	linear	non-linear
MarA-*mar*	308.5 ± 24.0	308.1 ± 24.1
Rob-*mar*	322.7 ± 26.5	322.2 ± 26.6
Rob-*mar*	316.5 ± 25.6	316.1 ± 25.6
Rob-*micF*	322.3 ± 23.9	321.8 ± 24.0
Rob_{ld}-*mar*	481.1 ± 22.9	480.9 ± 23.0
Rob_{ld}-*micF*	472.6 ± 34.5	472.4 ± 34.6

[a] All energies are shown in kcal mol^{-1}, and are average values and standard deviations calculated over 5000 snapshots extracted every 50 ps of 5 x 2.5 μs MD trajectories for all systems, calculated with 80% box filling, an internal dielectric of 10, and, in the case of the non-linear equation, a relaxation parameter of 1.0. Values were calculated using both the linear and non-linear Poisson-Boltzmann equations. Energies were calculated using the Delphi PBE solver[15, 16].

Supplementary References

1. Roe, D. R.; Cheatham 3rd, T. E., PTRAJ and CPPTRAJ: Software for Processing and Analysis of Molecular Dynamics Trajectory Data. *J. Chem. Theory Comput.* **2013,** *9*, 3084-3095.

2. Tribello, G. A.; Bonomi, M.; Branduardi, D.; Camilloni, C.; Bussi, G., PLUMED 2: New Feathers for an Old Bird. *Comput. Phys. Commun.* **2014,** *185*, 604-613.

3. Rhee, S.; Martin, R. G.; Rosner, J. L.; Davies, D. R., A Novel DNA-Binding Motif in MarA: The First Structure for an AraC Family Transcriptional Activator. *Proc. Natl. Acad. Sci. USA* **1998,** *95*, 10413-10418.

4. Berman, H. M.; Westbrook, J.; Feng, Z.; Gilliland, G.; Bhat, T. N.; Weissig, H.; Shindyalov, I. N.; Bourne, P. E., The Protein Data Bank. *Nucleic Acids Res.* **2000,** *28*, 235-242.

5. Kwon, H. J.; Bennik, M. H.; Demple, B.; Ellenberger, T., Crystal Structure of the *Escherichia coli* Rob Transcription Factor in Complex with DNA. *Nat. Struct. Biol.* **2000,** *7*, 424-430.

6. Kabsch, W.; Sander, C., Dictionary of Protein Secondary Structure: Pattern Recognition of Hydrogen-Bonded and Geometrical Features. *Biopolymers* **1983,** *22*, 2577-637.

7. Skjaerven, L.; Yao, X. Q.; Scarabelli, G.; Grant, B. J., Integrating Protein Structural Dynamics and Evolutionary Analysis with Bio3D. *BMC Bionformat.* **2014,** *15*, 399.

8. Kollman, P. A.; Massova, I.; Reyes, C.; Kuhn, B.; Huo, S.; Chong, L.; Lee, M.; Lee, T.; Duan, Y.; Wang, W.; Donini, O.; Cieplak, P.; Srinivasan, J.; Case, D. A.; Cheatham, T. E., Calculating Structures and Free Energies of Complex Molecules: Combining Molecular Mechanics and Continuum Models. *Acc. Chem. Res.* **2000,** *33*, 889-897.

9. Li, Z.; Demple, B., Sequence Specificity for DNA Binding by *Escherichia coli* SoxS and Rob Proteins. *Mol. Microbiol.* **1996,** *20*, 937-945.

10. Maier, J. A.; Martinez, C.; Kasavajhala, K.; Wickstrom, L.; Hauser, K. E.; Simmerling, C., ff14SB: improving the Accuracy of Protein Side Chain and Backbone Parameters from ff99SB. *J. Chem. Theory Comput.* **2015,** *11*, 3696-3713.

11. Wang, J.; Wolf, R. M.; Caldwell, J. W.; Kollman, P. A.; Case, D. A., Development and Testing of a General AMBER Force Field. *J. Comput. Chem.* **2004,** *25*, 1157-1174.

12. McGaughey, G. B.; Gagne, M.; Rappe, A. K., Pi-Stacking Interactions. *J. Biol. Chem.* **1998,** *273*, 15458-15463.

13. Burley, S. K.; Petsko, G. A., Aromatic-Aromatic Interaction: A Mechanism of Protein Structure Stabilization. *Science* **1985,** *229*, 23-28.

14. Zhao, Y.; Li, J.; Gu, H.; Wei, D.; Xu, Y. C.; Fu, W.; Yu, Z., Conformational Preferences of Pi-Pi Stacking Between Ligand and Protein, Analysis Derived from Crystal Structure Data Geometric Preference of Pi-Pi Interaction. *Interdiscip. Sci.* **2015,** *7*, 211-220.

15. Li, L.; Li, C.; Sarkar, S.; Zhang, J.; Witham, S.; Zhang, Z.; Wang, L.; Smith, N. C.; Petukh, M.; Alexov, E., DelPhi: A Comprehensive Suite for DelPhi Software and Associated Resources. *BMC Biophys.* **2012,** *5*, 9.

16. Li, C.; Jia, Z.; Chakravorty, A.; Pahari, S.; Peng, Y.; Basu, S.; Koirala, M.; Panday, S. K.; Petukh, M.; Li, L.; Alexov, E., DelPhi Suite: New Developments and Review of Functionalities. . *J. Comput. Chem.* **2019,** *40*, 2502-2508.
